# Latent Factor Analysis to Discover Pathway-Associated Putative Segmental Aneuploidies in Human Cancers

**DOI:** 10.1371/journal.pcbi.1000920

**Published:** 2010-09-02

**Authors:** Joseph E. Lucas, Hsiu-Ni Kung, Jen-Tsan A. Chi

**Affiliations:** 1 Institute for Genome Sciences and Policy, Duke University, Durham, North Carolina, United States of America; 2 Department of Molecular Genetics and Microbiology, Duke University, Durham, North Carolina, United States of America; Lilly Singapore Centre for Drug Discovery, Singapore

## Abstract

Tumor microenvironmental stresses, such as hypoxia and lactic acidosis, play important roles in tumor progression. Although gene signatures reflecting the influence of these stresses are powerful approaches to link expression with phenotypes, they do not fully reflect the complexity of human cancers. Here, we describe the use of latent factor models to further dissect the stress gene signatures in a breast cancer expression dataset. The genes in these latent factors are coordinately expressed in tumors and depict distinct, interacting components of the biological processes. The genes in several latent factors are highly enriched in chromosomal locations. When these factors are analyzed in independent datasets with gene expression and array CGH data, the expression values of these factors are highly correlated with copy number alterations (CNAs) of the corresponding BAC clones in both the cell lines and tumors. Therefore, variation in the expression of these pathway-associated factors is at least partially caused by variation in gene dosage and CNAs among breast cancers. We have also found the expression of two latent factors without any chromosomal enrichment is highly associated with 12q CNA, likely an instance of “trans”-variations in which CNA leads to the variations in gene expression outside of the CNA region. In addition, we have found that factor 26 (1q CNA) is negatively correlated with HIF-1α protein and hypoxia pathways in breast tumors and cell lines. This agrees with, and for the first time links, known good prognosis associated with both a low hypoxia signature and the presence of CNA in this region. Taken together, these results suggest the possibility that tumor segmental aneuploidy makes significant contributions to variation in the lactic acidosis/hypoxia gene signatures in human cancers and demonstrate that latent factor analysis is a powerful means to uncover such a linkage.

## Author Summary

Gene signatures are a powerful tool to investigate biological processes in human cancer. However, it is clear that these gene signatures do not fully reflect the complexity of human cancer. Here we demonstrate how a latent factor model can improve the in vivo relevance of these pathway-associated gene signatures by dissecting them into co-regulated transcriptional components which better represent the structure in human cancer. We use this approach to analyze hypoxia and lactic acidosis gene signatures to identify latent factors that represent distinct, interacting components of the various biological processes which are in the initial gene signatures but poorly dissected. Some factors are clustered in small chromosomal regions and their expression values are highly correlated with their DNA copy number in both cancer cell lines and human tumors. Therefore, the gene dosage at the DNA levels may explain the differences in gene expression. Several factors contain genes which are known to directly modulate the hypoxia response and allow us to generate testable hypotheses regarding particular copy number changes and hypoxia signatures. Therefore, the use of latent factor analysis is a powerful means to identify pathway-associated changes in the DNA copy number and gene dosage.

## Introduction

### The promise and challenge of cancer genomics

Human cancers are extremely heterogeneous due to multiple mutations in oncogenes and tumor suppressor genes, varying environmental conditions, and a huge range of germline and somatic variations. While the individual effects of a genetic alteration or environmental factors may be quite subtle, their combined effects lead to immense natural heterogeneity in tumor phenotypes, disease outcomes, and response to therapies. The use of microarrays to capture global gene expression patterns in human cancers has lead to an explosion of knowledge regarding the genetic basis of cancer heterogeneity. Experiments that have previously been performed one gene at a time can now be done on the entire complement of transcribed genes. However, this leads to a tremendous challenge of divining meaning behind the vast amounts of biological data and turning it into hypotheses and new understanding of the biology behind tumor heterogeneity.

### Gene signature approaches for cancer gene expression

In relating tumor gene expression data to tumor heterogeneity, one powerful approach is the use of gene signatures to dissecting the complexity of cancer genomic data. These gene signatures represent a set of genes which are coordinately regulated in particular biological processes and specific perturbations, first determined based in cultured cells or other supervised analysis to represent particular biological processes and [1]–[8]. The expression signatures are portable and can be assayed in varied contexts, and so provide the capacity to link otherwise heterologous systems to provide a mechanism to link the defined biological processes with the complex phenotypes of human tumors. These signatures can then be used to recognize similar molecular features in human cancer samples in vivo and interrogate the relevance of particular biological processes and perturbations in human cancer and evaluate their relationship with other clinical and molecular features.

There are many different means to quantitatively define signature activities in human tumor gene expression datasets. One approach involves extracting the genes in a signature defined in an experimental setting and examining their co-variation in other datasets. These genes can then be used to reclassify tumors based on clustering of the expression of the genes in a pathway [2], [3]. Another approach ignores signatures and simply seeks to collect genes that demonstrate high levels of correlation across samples into gene modules. Classification and prediction tasks are then performed on the expression of modules rather than individual genes, leading to re-classification and functional annotation of human tumors [9]. It is also possible to use Bayesian statistics to determine the probability of the pathway activities to avoid the instability of hierarchical clustering [4], [10]. Gene set enrichment analysis (GSEA) seeks to compare observed expression patterns to pre-defined, curated pathways [11]. Finally, the connectivity map [12] uses a similar approach to establish connections with perturbations due to the presence of drugs and other small molecules. We have also used these and similar approaches to show that wound healing [2], vascular injury responses [13] and various oncogenic mutations [4], [10], [14]–[17] can play important roles in tumor progression.

### Poorly dissected complex structures of gene signatures in vivo

Although this projection of various gene signatures onto heterologous gene expression data of human cancer in vivo has been quite successful, there are also significant limitations. The gene signatures which have been defined in vitro using cultured cells simply cannot fully reflect the complexity of variation seen in human cancers. This discrepancy can be due to many reasons. For example, there may be several components of pathway signaling observed *in vitro*, but which are subject to multiple regulatory controls that break down the clear patterns in vivo. Some genes may be better representatives of pathway activity in vivo because they are less likely to be involved in other pathways, or because they react to environmental conditions that are not present *in vitro*; others present in the experimental signature may be unexpressed in vivo. For example, it is known that Ras has at least three major downstream pathways - Ral, Raf and PI3K. The activation and role of each pathway may be different under oncogenic transformation and tumor maintenance [18], [19]. Furthermore, cancer cell genomes have many amplifications, deletions and point mutations (copy number alterations (CNAs) or aneuploidies), any subset of which may modulate the pathway activity of both oncogenic signaling and microenvironmental responses. Since only normal or cloned cell lines are used to generate *in vitro* signatures, the consequences of these complex DNA alterations in tumors are not observed. Compositions of cell types in tumors also reflect the continuous evolution and selection of cells fittest to survive under harsh tumor microenvironmental stresses, possibly over years of development. In addition, human cancers also involve heterogeneous cell types with complex intercellular interactions as well as temporal and spatial variation in expression. These factors cannot be easily modeled *in vitro* using cell culture or captured using microarrays.

### Factor analysis to uncover in vivo complexity of original gene signatures

Our approach to address the limitations of the gene signatures is to apply statistical latent factor models using in vivo cancer data to further dissect the *in vitro* derived “primary signatures” into components which better represent the complexity and structure captured by the global gene expression of human cancers in vivo. Statistical analysis using latent factor models aims to address this by identifying and estimating potentially many factors in the in vivo expression patterns of sets of signature genes defined *in vitro*; these factors or “sub-signatures” retain their relationship to the original signature but represent distinct, interacting components of the biological processes the initial signature. This approach has been successfully applied to generate an elaborated picture of the complexity of patterns of variation shown by the signature gene set, and additional genes apparently related to the gene set, in observational contexts [14], [20]–[26]. Elaborating the factor profile underlying the original signature can improve the in vivo relevance by more fully describing the diversity of in vivo expression patterns, and may enhance prognostic value and provide mechanistic insights into how biological processes affect clinical phenotypes.

### Gene signature of tumor microenvironmental stresses

The tumor microenvironment is characterized by many chemical stresses, such as oxygen depletion (hypoxia), high lactate and extracellular acidosis (lactic acidosis) [27]. Given the importance of these stresses to cancer phenotypes and the recent efforts to develop therapeutic strategies targeting hypoxia pathways, a detailed understanding of the mechanisms and influences of these stresses in tumors will be of significant interest. We have previously used gene signature approaches to estimate the role of hypoxia [3] and lactic acidosis [8], [25] in the heterogeneity and clinical phenotypes of human cancer. The hypoxia signature obtained in cultured cells exposed to hypoxia allows the recognition of the molecular features common to multiple cancer types – in turn permitting the identification of patients with high clinical risks due to strong hypoxia response [3]. Additionally, linking prognostic molecular signatures of human cancers to *ex vivo* experimental cell culture models provides a relevant and controlled system that can be used in mechanistic studies. Patients who are most likely to benefit from targeted therapeutics can then be recognized by the high expression of the hypoxia gene signatures. Therefore, substantial synergy and the potential for novel biological insights can be obtained by reciprocal flow of information between the in vitro and in vivo systems. However, the basis for variation in the hypoxia and lactic acidosis signatures in tumors is entirely unknown.

Due to increased proliferation and defective mechanisms for monitoring genome integrity, one of the hallmarks of cancer is the presence of with alterations of single nucleotides or CNAs with the amplification/deletion of regions of chromosomes of various lengths. These mutations and CNAs are likely to contribute to the variations of the gene signatures of tumor microenvironmental stresses. For example, it is known that HIF transcriptional complexes and hypoxia pathways are constitutively activated in the patients with von Hippel-Lindau disease, a genetic disease in which the *VHL* gene is either inactivated or deleted [28]. In addition, tumor microenvironmental stresses may also select cancer cells with particular CNAs with strong metastasis phenotypes and invasive behaviors [27], [29], [30]. It is therefore interesting to identify CNAs associated with the hypoxia and lactic acidosis pathways in human cancers.

In this study, we seek to use sparse latent factor analysis to identify CNAs associated with the hypoxia and lactic acidosis response in human cancers. The work that we present here is based on the model described in [20], but should be repeatable with any version of factor models. Specifically, we fit a latent factor model of the gene signatures of hypoxia and lactic acidosis in one data set of 251 breast tumors (Miller) from [20] to generate 56 latent factors. These factors then allow for connections to be made between a numbers of different data sets, which can be used to generate biological hypotheses regarding the basis for the variation in the gene expression signatures of hypoxia and lactic acidosis. We have identified variation in the expression of several factors on the RNA level which are highly associated with CNAs in similar or distinct chromosomal regions. Our findings lead to multiple, easily testable hypotheses about some critical genes in relation to the dyregulation of the hypoxia pathway in human cancers. Taken together, these results suggest the possibility that tumor segmental aneuploidy makes a significant contribution to the variation in the hypoxia and lactic acidosis gene signatures seen in human cancers and demonstrates that latent factor analysis is a powerful means to uncover such a linkage.

## Results

### Identify the BAC clones associated with the hypoxia and lactic acidosis gene signatures

**Figure 1:**
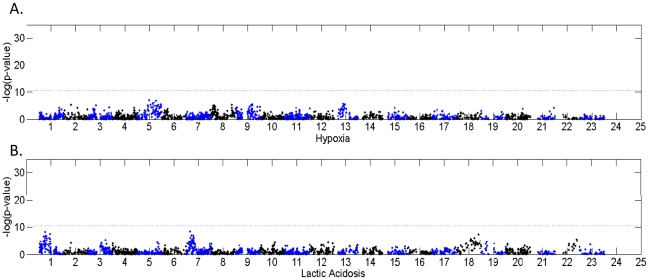
The association of BAC clones with the hypoxia and lactic acidosis gene signatures. The correlation between signature – hypoxia (A) and lactic acidosis (B) - and copy number change in the breast cancer data set from Chin. The x-axis shows the location along the genome of the different CGH clones, and the y-axis shows the level of association (−ln(p-value) of Pearson correlation). Dashed lines are drawn at alpha = .05 after Bonferroni correction for multiple testing (2150 tests, one for each CGH clone). We note that the signatures alone do not show strong correlation with any particular genomic location, which makes it impossible to assess relationships between signatures and CNA without some way to break up the signatures into smaller groups.

Chromosomal aneuploidy and CNAs are known to lead to changes in the RNA expression levels of genes in the corresponding chromosomal regions and contributes to the altered gene expression deregulation of myriad pathways during oncogenesis. Given the potential contribution of the CNAs to variation in gene expression, and the likely survival advantages (to the tumor cells) under selection due to microenvironmental stresses, we seek to identify the CNAs associated with the expression of hypoxia and lactic acidosis gene signatures in tumors. In a previous study, the gene expression of wound signatures was used to identify CNAs in breast cancers as potential regulators of such gene expression program [31]. We used similar approach to identify tumor CNAs associated with the hypoxia/lactic acidosis gene signatures by examining a breast cancer expression dataset (Chin) with both gene expression and CNAs variations based on comparative genomic hybridization (CGH) [32]. We projected the hypoxia and lactic acidosis gene expression onto the Chin data set and calculated the Pearson correlation against the measurements of each of the 2150 BAC clones for these tumors (Figure 1). There are some chromosomal locations that show associations between the hypoxia and lactic acidosis signatures, such as 1p and 7p for lactic acidosis and 1q, 5q and 13p for hypoxia. But we found no statistically significant associations between signatures and CNAs at any locations in the genome after using the strict alpha level of 0.01 after Bonferroni correction.

### Use of human cancers to dissect the gene signatures into multiple latent factors

The modest correlation between the BAC clones with the hypoxia and lactic acidosis signatures may be due to the complex composition of gene signatures in human tumors and other confounding factors. In order to further dissect such complexity, we used a latent factor model to break these signatures down based on coherent expression in tumor tissue and to test for association between components (sub-signatures) of the gene signatures and CNAs. Factor analysis is becoming a standard technique for the analysis of microarray expression data. In general, it is a simple factorization of a matrix of data into the product of two other matricies. In our case, if *X* is a *PxN*-dimensional matrix of expression values, then we write
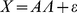
(1)
*A* is called the factor loadings matrix (a matrix of regression coefficients) and *Λ* is a matrix of factor scores (this may also contain design vectors). Finally, *ε* is the *PxN*-dimensional matrix of idiosyncratic errors. We assume *ε_i,j_∼N(0,σ_i_^2^)* which implies that the corresponding PxP covariance matrix, which we label *V*, is diagonal with σ^2^_i_ in the *i^th^* position. While this may seem restrictive, it is exactly the covariance structure in *X* that is being described by the latent factors, and therefore more complex covariance structure in *ε* would be redundant and potentially lead to issues with identifiability. There are multiple versions latent factor models in the published literature, including principal components [33], non-negative matrix approximation [34], sparse latent factor models [20] and partial least squares [35]. We utilized the version from [20] called Bayesian factor regression models (BFRM), but our general approach can be applied using any version of factor modeling. BFRM tries to make choices of *A* and *Λ* such that there are a large number of zeros in the matrix *A*, thereby creating a parsimonious model of the variation seen in *X*.

One of the key features of factor models is the ability to project factors discovered in one data set onto another. This allows the comparison of phenotypes across different data sets, such as hypoxia linked to expression in one data set and CNA linked to expression in another data set. In order to assess the relationship between derived latent factors and interventions/variables from other experiments, we need to be able to estimate *Λ* in a new data set, given a previously discovered loadings matrix, *A*. This is a well known problem of inverse regression and we will utilize the approach described in [36]. We define *Y* to be a new set of expression data, and suppose we wish to estimate the factor scores on this data set, *Λ_y_*. Then if *A* is the matrix of factor loadings (regression coefficients) from model fitting and *V* is a diagonal matrix containing the gene by gene variance estimators, we compute
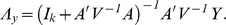
(2)(In this equation, *I_k_* is a *k*-dimensional identity matrix.) This allows us to build a factor model on any data set and project those factors onto any other data set in which we have measurements of all of the relevant probe sets.

**Figure 2:**
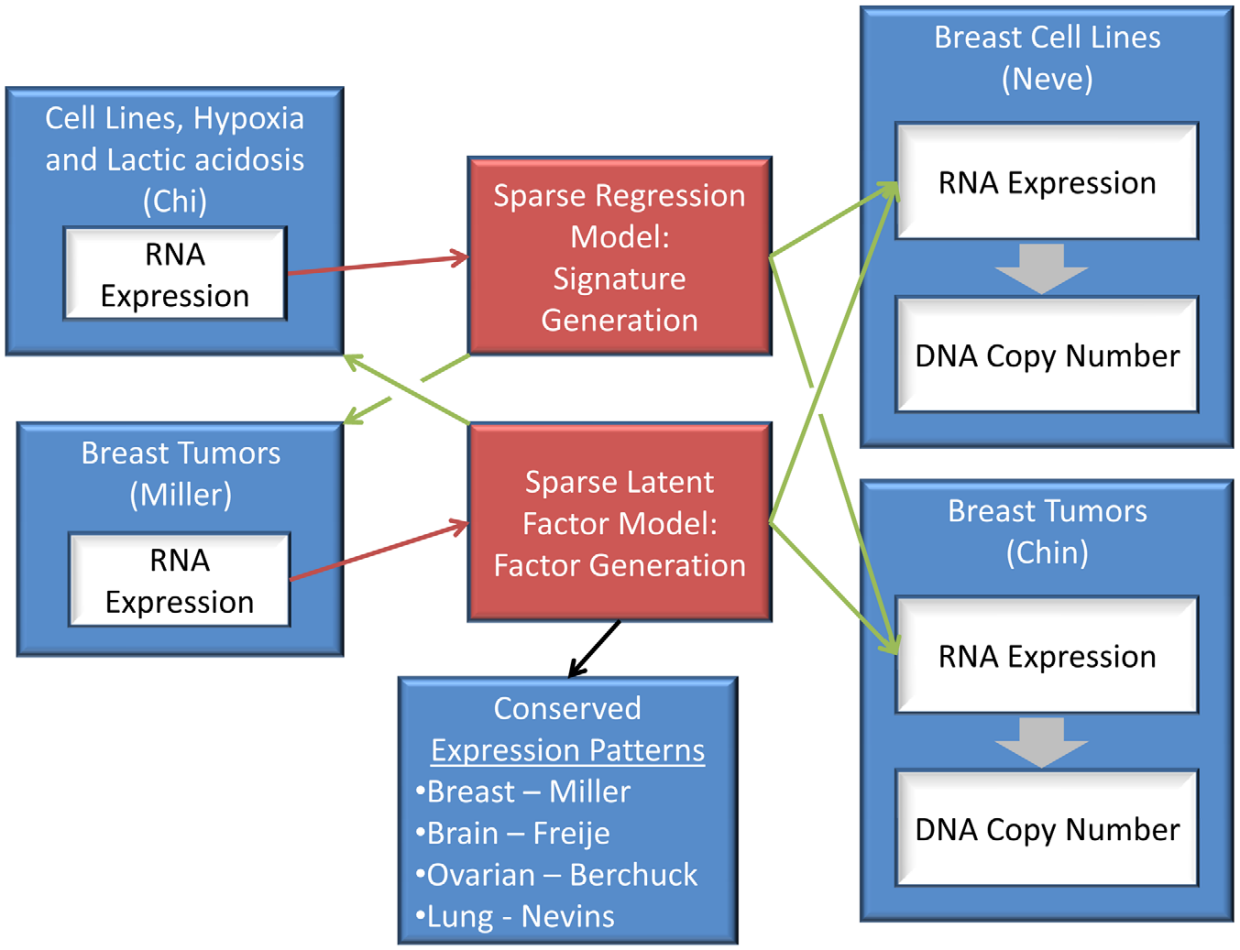
The flow chart of integrative genomic analysis. The flow chart shows the myriad data sets and data types that we integrate to generate the use cases outlined in this section. All case studies utilize a set of 56 factors built with sparse latent factor models from the Miller breast tumor data or the two signatures generated from the Chi data set. The red arrows represent the training of a model with a given data set. Green arrows represent projection of a set of factors or signatures onto a new data set, and the black arrow represents a visualization technique (shown in Figure 2). The fat gray arrows represent Pearson correlation calculations.

**Figure 3:**
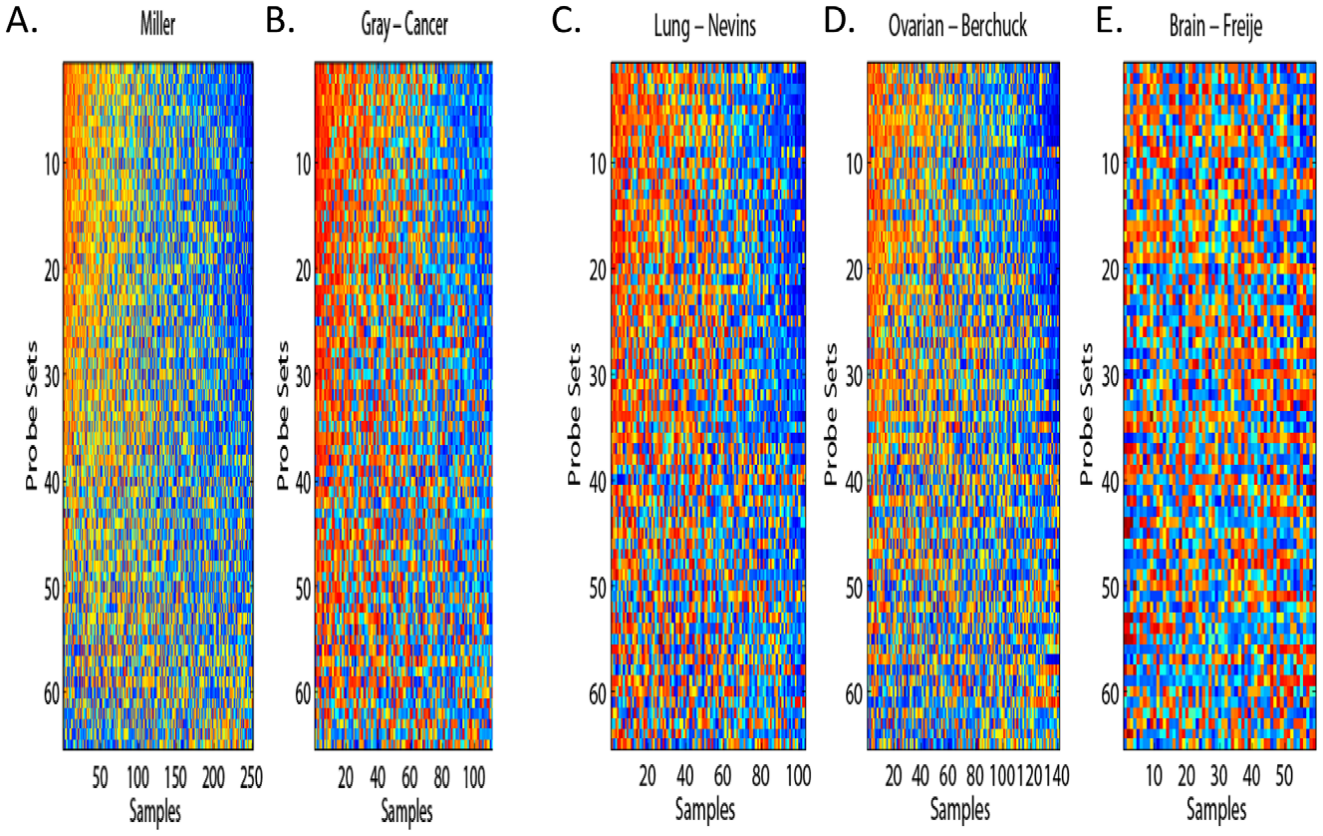
The coordinated gene expression of latent factors in various human tumors. The expression of genes in the factor 26 in the Miller breast cancer datasets from which the factor was first derived (panel A). Similar coordinated expression is also found for expression data of another breast cancers (Gray - panel B), Lung (Nevins - panel C) and Ovarian (Burchurk - panel D) cancers. But such coordinated expression is not found in the brain (Frejie - panel D) cancers. Ordering of the rows (bottom to top) is increasing in the loading of the first principal component in the Miller data set. This row order is retained in all five heatmaps. Ordering of the columns (from left to right) is increasing in the first principal component. This is recalculated for each heat map.

The ability to project factor models onto different data sets allows the possibility of comparing new experimental data sets, such as the hypoxia and lactic acidosis signature experiment, to any of the thousands of publically available data sets with different levels of information, including CNAs from array CGH. The overall analysis scheme of our approach for the rest of the paper is presented in Figure 2. We note that there is an important assumption which is implicit in equation 2. We assume that the factor loadings obtained from the analysis of the first data set are valid for the analysis of the second data set. In the context of sparse factor modeling, we know that genes that share non-zero loadings for a specific factor also share elements of the expression pattern described by that factor. The assumption that the loadings matrix remains unchanged from data set to data set translates into the assumption that genes which show co-expression in one data set will continue to show co-expression in a new data set. It is evident that the extent to which this will hold true is dependent on the character of the two data sets in question. However, Figure 3 and its accompanying analysis demonstrate that this paradigm may hold true in a larger array of data sets than one might expect, such as across different tumor types.

Because we are interested in the relationships between gene expression and the tumor microenvironmental stresses, we restricted our attention to a set of 2984 genes whose expression were found to be affected under conditions of hypoxia and lactic acidosis [25]. The matricies (*A* and *Λ*) that are derived in this analysis, along with the parameter file used by BFRM, are included in the statistical supplement (Text S1). Fitting the latent factor model to these genes in a breast tumor expression dataset (Miller) [37] of 251 tumors, we obtained 56 latent factors (genes in each factors presented in supplementary Text S2). The expression of these factors is largely coordinately expressed across tumors in the Miller dataset, as exemplified for factor 26 in Figure 3A. In addition, the relationship of these genes is mostly conserved in an independent dataset of 118 breast tumors [32] (Figure 3B, labeled as Gray dataset, p = 2.2×10^−15^). The estimation of the statistical significance of coordinate expression in different tumor expression data sets is discussed in the methods section. Although we don't expect coordinate gene expression to persist in other tumor types, we find that coherent expression of the genes in factor 26 is also preserved in lung (p = 7.3×10^−8^) and ovarian cancers (p = 7.6×10^−10^) (Figure 3C, D) [1], [38], but is largely lost in brain cancers (p = .10) (Figure 3F) [39]. We find that this is generally the rule, and not the exception, for most factors. Figures showing the expression of the genes in all 56 latent factors in these five human tumor datasets are included in the Figure S1.

### Biological annotation of the latent factors in human cancers

To further functionally annotate these coordinately expressed genes in the discovered latent factors, we used a web-based statistical tool Gather [40] to test whether the genes in each factor are significantly enriched in Gene Ontology (GO) or chromosomal locations. This analysis found that 30 latent factors are significantly enriched in at least one GO (p<0.001). These factor-enriched GO terms include glycolysis/gluconeogenesis (factor 9), unfolding protein response (factor 10), neoplasm metastasis (factor 15), TGF-β (factor 19) and immune response (factor 6 and 16) (Table S1, Table S2, Table S3). Interestingly, many of these biological processes have been previously shown to be linked with hypoxia and/or lactic acidosis in many studies. For example, hypoxia is known to trigger gene expression pathways related to glycolysis as well as genes in the TGF-β pathway and epithelial-mesenchymal transition [41]–[43]. Additionally, hypoxia and acidosis are also known to trigger tumor metastasis [44], [45] and the “unfolding proteins response” [46]–[48]. The discovery of factors representing these processes is encouraging and indicates that factor analysis has the potential to uncover links between these processes and the influence of hypoxia and lactic acidosis in human cancers.

### Statistics to evaluate chromosomal enrichment of genes in factors – local enrichment ratio

In addition to enrichment for biological processes, gather analysis indicates that 22 latent factors also exhibit significant spatial basis with significant enrichment (p<0.001) in different chromosomal locations (Table S1). Such spatial enrichment suggests that events in these chromosomal regions may contribute to their coordinated expression in tumors. We devised a separate measurement and statistic, the local enrichment ratio (LER), to assess the enrichment of latent factors for location specific chromosomal association. Given a set of genes, the statistic is based on the ratio of two distributions: 1) the distribution along the genome of the genes in the set and 2) the distribution along the genome of the collection of all possible genes that might have been in the list. In our case, the gene subset will consist of genes in a factor and the full list will consist of the 2984 genes from which we built our factor model (detailed in the methods section).

This is a similar approach to that taken in KC-SMART [49] for the analysis of CGH data with two key differences. First, we are applying our approach to specific subsets of genes (each of our 56 factors) and testing the hypothesis that those subsets are enriched for a particular location. This is in contrast to KC-SMART [49], which is a general test designed to identify regions of CNA, and as such is not directly applicable. Second, we are applying our model to genes that have been grouped based on mRNA coexpression, thus our test is performed completely independent of data on copy number variation such as array CGH.

**Figure 4:**
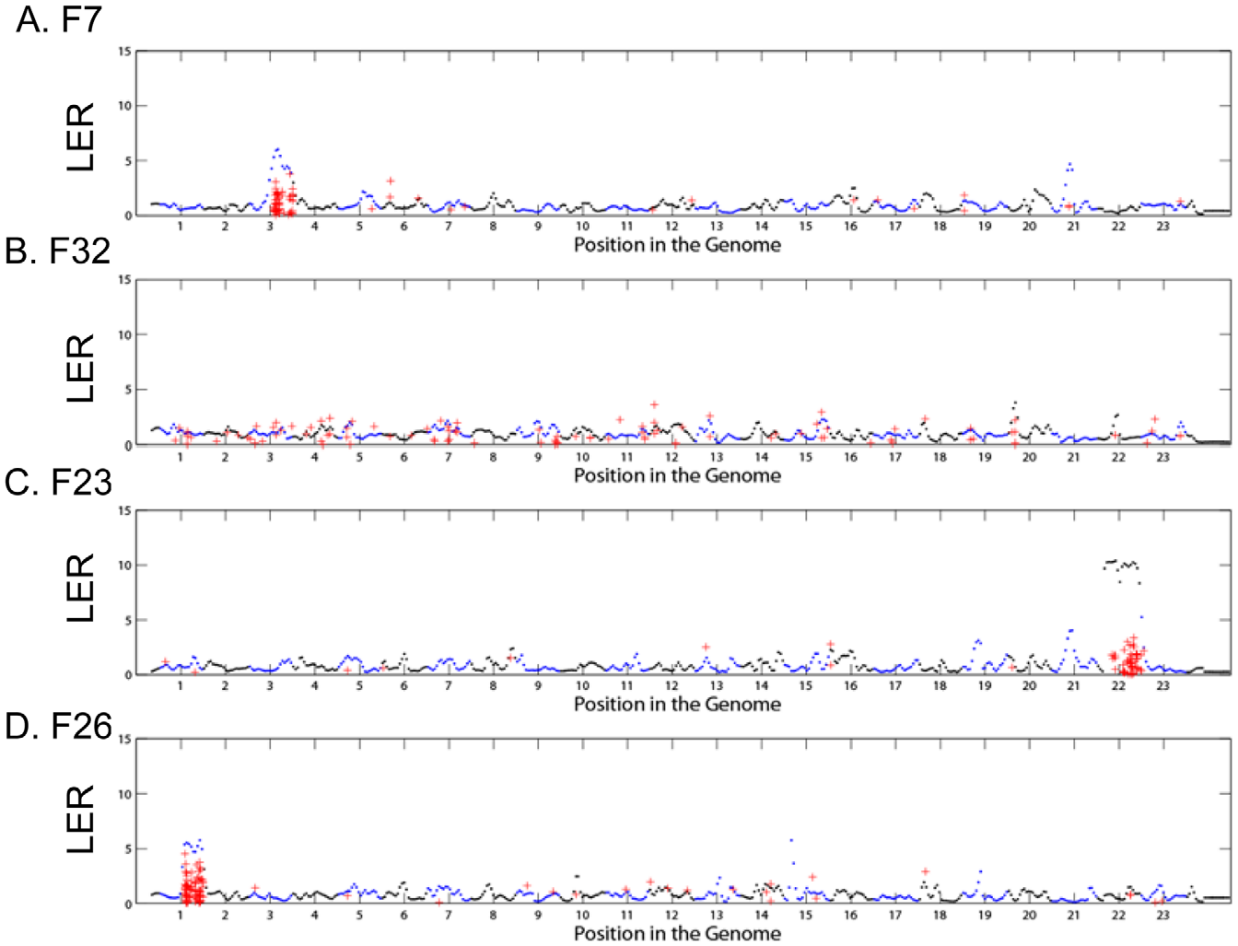
The local enrichment ratios as measures of chromosomal enrichments of latent factors. Local enrichment ratios (y-axis) for the genes in latent factor 7, 32, 23 and 26 (A,B,C and D respectively) along the 23 autosomes (X-axis). Chromosomes are colored alternately blue and black for visualization. The red crosses designate the physical locations of the genes in the respective factor with vertical jitter added to allow visualization. A local enrichment ratio over 5 is considered significant, therefore we conclude that factors 7, 23 and 26 demonstrate significant enrichment for genes in specific chromosomal regions. While 32 demonstrates no local enrichment.

When we plot the calculated LER for the genes in each latent factor along the 23 chromosomes, we observe prominent peaks for several spatially-biased latent factors (Figure 4A, C, D) in one or two chromosomal locations against the background of noise in other locations. In contrast, factors without any significant chromosomal enrichment produce broad, non-specific peaks with low scores (Figure 4B). We find that the genes in 18 out of the 56 discovered factors exhibit local enrichment ratios of over 3 for particular chromosomal locations (Table S1). This independent measurement of chromosomal enrichment shows a high degree of agreement with gather analysis. We do not have a theoretical distribution for random draws of this ratio under conditions of no CNA. However, simulation studies using random draws from the 2984 hypoxia/lactic acidosis genes, using a kernel standard deviation of 5% of the length of the chromosome, suggests that the probability of generating a maximum local enrichment ratio over 3 across the whole genome, given the null hypothesis, is less than 1×10^−4^. Matlab code for computing the local enrichment ratio is included in the statistical supplement (Text S1). Figures showing LER for all 56 latent factors are included in the Figure S2. We have tested various window sizes and have determined that these findings are relatively insensitive to kernel width (Figure S3).

### The association of CNAs with expression of latent factors in tumors

**Figure 5:**
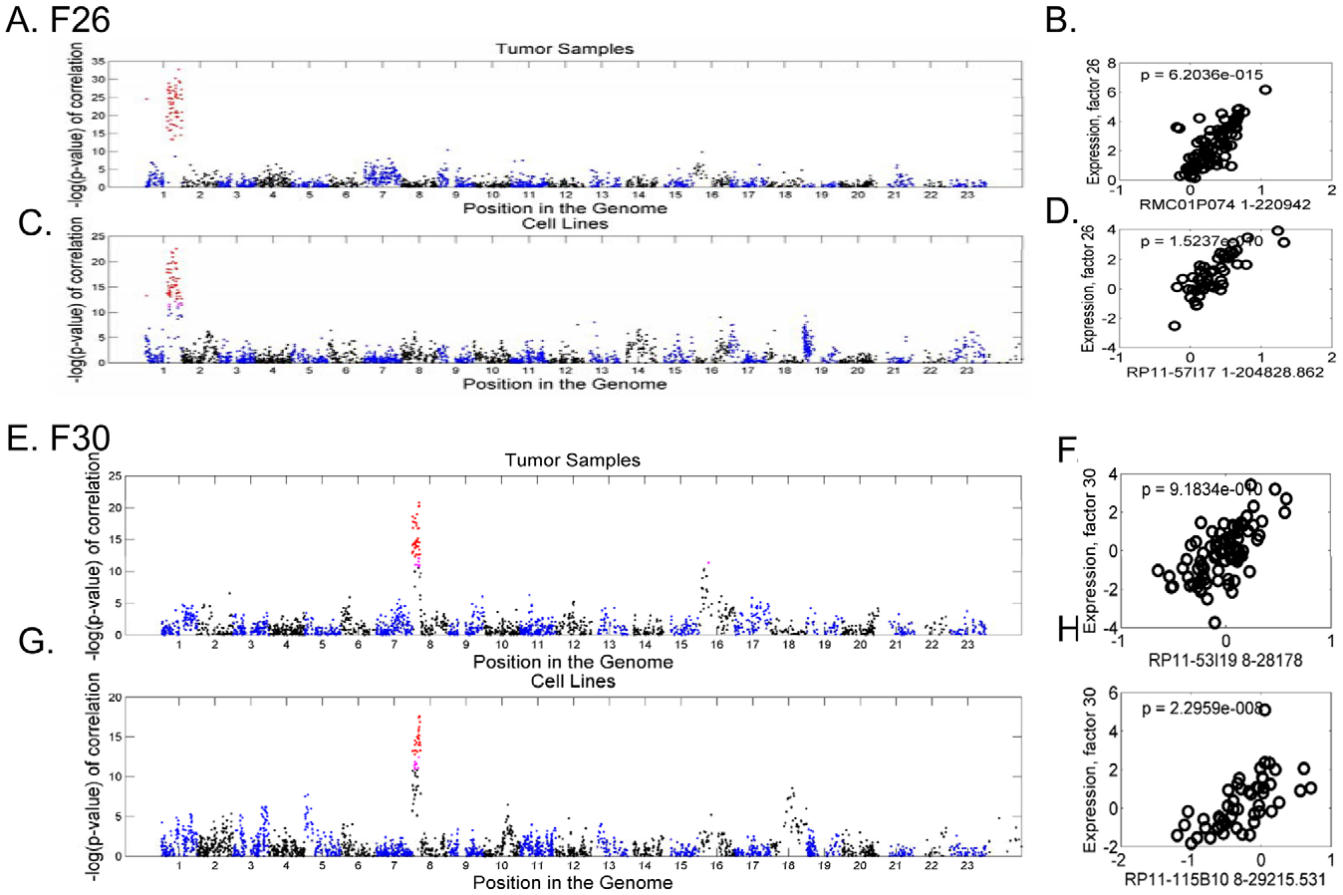
The association of mRNA gene expression and DNA copy numbers of latent factors. The degree of association (−log (p value) of Pearson correlation, Y-axis) between the expression of factor 26 with the BAC clones along the 23 chromosomes (X-axis) in breast tumors, A, and cancer cell lines, C, with the significant association with indicated BAC clones in 1q (panels B and D). Similar analysis has been performed for genes in factor 23 in breast tumors (panels E and F) and cancer cell lines (panels G and H) with indicated BAC clones in 22q.

The association of many factors with particular chromosomal locations suggests that their co-variation in cancer gene expression may be due to gene dosage caused by CNAs or other spatially-biased gene regulations in those chromosomal regions. To test these possibilities, we project our factors into a breast cancer data set (Chin et al) [32] as well as breast tumor cell lines (Neve et al) [50] both with both gene expression and CNA data. The expression scores of the 56 latent factors were assessed on both tumors samples and cancer cell lines. These were then compared with the 2150 CGH clones in the corresponding tumor and cell line samples using Pearson correlation. Plots showing the strength of correlation for two of these factors are shown in Figure 5. Although many factors show no particular association, approximately 1/3 factors do show a significant degree of association between factor expression and BAC clones in small chromosomal regions in both tumors and cell lines. Using a filtering criteria of minimum p-value less than .01 after Bonferroni correction for 2150 hypothesis tests in both tumor and cell line data sets, and requiring the genes defining the factor to show a significant overabundance in the same chromosomal region, we identified 17 factors both statistically and structurally associated with CNA regions of different sizes (Figure 5A, B and Figure S4). This high degree of association in both tumors and cancer cell lines strongly suggests that these 17 factors are indeed related to CNAs such as segmental aneuploidies. For example, factor 26 is shown to be linked to CNA on chromosome 1 in human tumors (Figure 5A) with the strongest association between the factor and BAC clone RMC01P074 (Figure 5B). Similarly, among breast cancer cell lines, the expression of factor 26 is shown to be linked to CNAs on chromosome 1q (Figure 5C), with the highest association with BAC clone RP11-57I17 (Figure 5D). These results are entirely consistent with the results of enrichment in 1q from the analysis using both Gather and LER. Taken together, the variation in the expression of the genes comprising factor 26 are highly associated with the gene dosages and chromosome 1q CNA. Amplification of 1q has been previously noted in breast cancer and is associated with important clinical outcomes [32]. The re-discovery of this CNA associated with factor 26 validates our approach. Another example is that the expression of factor 30 is highly associated with segmental aneuploidies in 8p21–23 in both tumors (Figure 5E, F) and cancer cell lines (Figure 3G, H). It is interesting to note that many genes in the factor 30 are known to be hypoxia-inducible (e.g., clusterin [51] and stanniocalcin 1 [52], [53]) and may represent the CNAs of HIF-1α target genes. Among the CNAs associated with the 17 latent factors (included in Text S3), many have been previously recognized as high copy amplification regions in the original study of array CGH (Table S1). It is interesting that three of these CNA-associated factors – 8q21–24 (factor 35), 17q21–25 (factor 5, 17), 20q13 (factor 46)—are also reported to be linked with poor prognosis in another independent breast cancer study using a spatial enrichment of gene expression [54].

The incorporation of the coordinated gene expression pattern in latent factors may allow the recognition of CNAs with a higher confidence due to their impact on gene expression than from the mere use of BAC data or the examination of the spatial bias of gene expression. Such an approach is especially of value in the instances of low level of amplification and may explain the confident identification of several new putative CNAs (Table S1). Of these, 3q21–25 (factor 7) has been reported to be linked with poor prognosis in breast cancers [54].

Several factors found to be enriched in chromosomal locations based on the Gather and LER analysis did not show similar significant correlation with CNAs in the tumors and cancer cell datasets. This discrepancy may be due to the inconsistency between different datasets or tumors from the cell lines or clustered chromosomal locations in the same biological pathways show clustered in chromosomal location.

### Latent factors reflect potential trans-relationships between CNAs and expression

**Figure 6:**
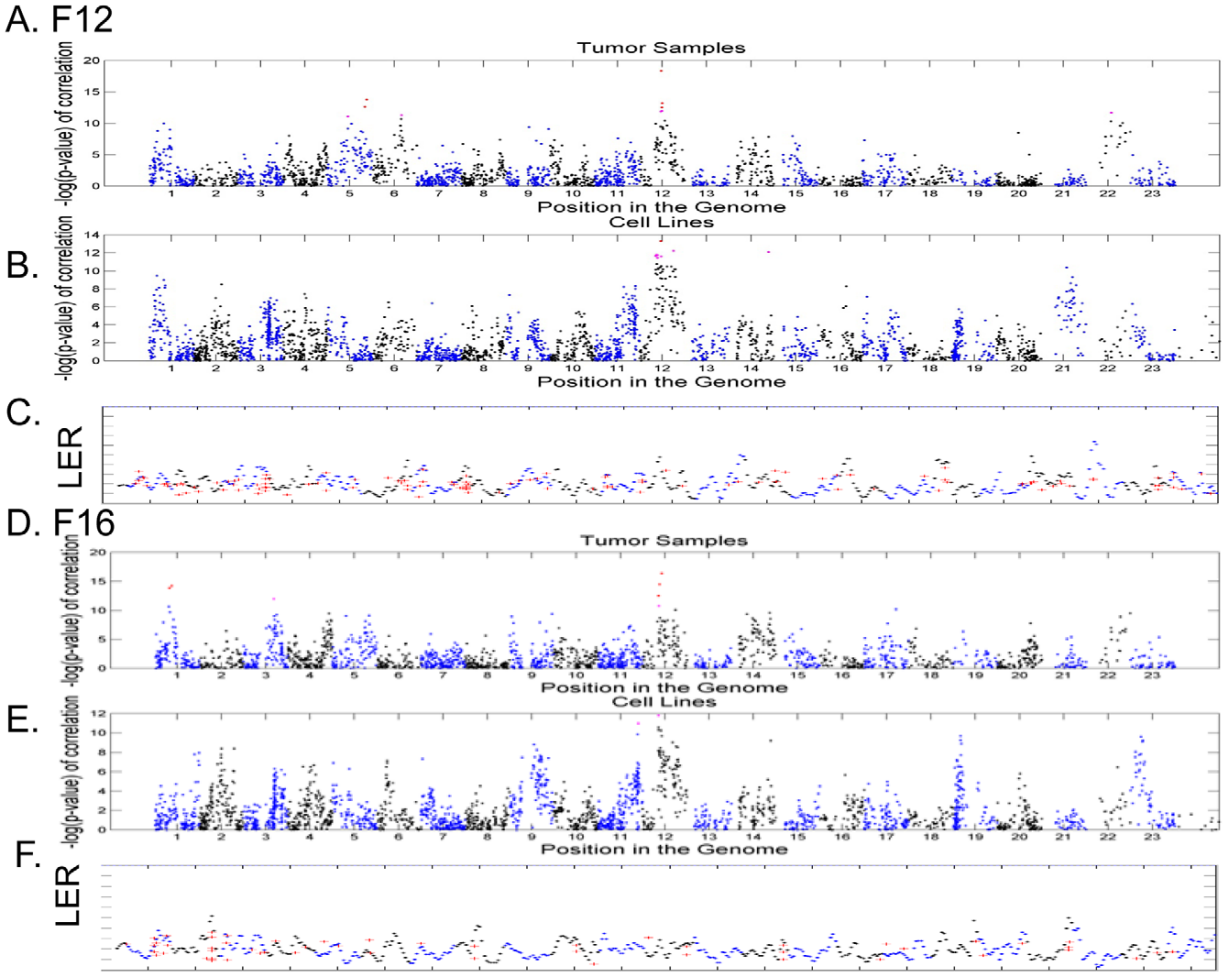
The association distant DNA copy numbers with the gene expression two latent factors. The degree of association in p value (Y-axis) is plotted between the expression of factor 12 with the BAC clones along the 23 autosomes (X-axis) in the breast tumors, A, and cancer cell lines, B, with the significant association with indicated BAC clones in 12q. Similar figures for factor 16 are shown in D and E. The red crosses in C and F designate the physical locations of the genes in latent factor 12 along the 23 autosomes (X-axis) with the degree of spatial enrichment (LDR) shown as a density ratio (Y-axis).

Most of the factors are associated with the CNAs in the chromosomal regions where the genes in the factors reside and suggest that such variations in “cis” are due to the variations in gene dosage caused by CNAs. We also detect a strong relationship between the expression of both factor 12 and 16 with CNA in a small region in the center of chromosome 12q in both tumors (Figure 6A, D) and cancer cell lines (Figure 6B, E). In contrast to the significant LER enrichments for other “cis-”association factors (Figure 4), the genes in these two factors show no spatial bias and enrichment by either GATHER or LER (Figure 6C, F). Instead, the genes in these two factors are scattered along various chromosomes without significant clustering (Figure 6C, F). It is not clear how the CNAs in particular chromosomal regions can lead to the coordinated expression of genes in these two factors without any chromosomal spatial bias. One possible explanation is that factors 12 and 16 may represent instances of “trans”-regulation in which segmental aneuploidies leads to transcriptional responses of these genes in the factors, instead of gene dosage effects in most other factors. It is also relevant to point out that factor 12-asscoated CNAs in 12q14–q15 has been noted to be prominent in other tumors, such as liposarcomona [55], glioma [56] and rhabdomyosarcoma[57]. This region contains HMGA2, a well known factor involved in the transcriptional regulation of oncogenesis [58].In addition, two closely located genes, MDM2, YEAST4, are critical to the p53 pathway. Although this is a possible explanation, this possibility remains to be experimentally tested.

### Factor 26 as a predictor of low hypoxia response

In the sections below, we will present several examples of biological hypotheses resulting from our analysis. It is important to note that these hypotheses will still need to be validated. However, it is the ability to generate hypotheses by bringing together disparate data sets that represents the power and novelty of our approach.

**Figure 7:**
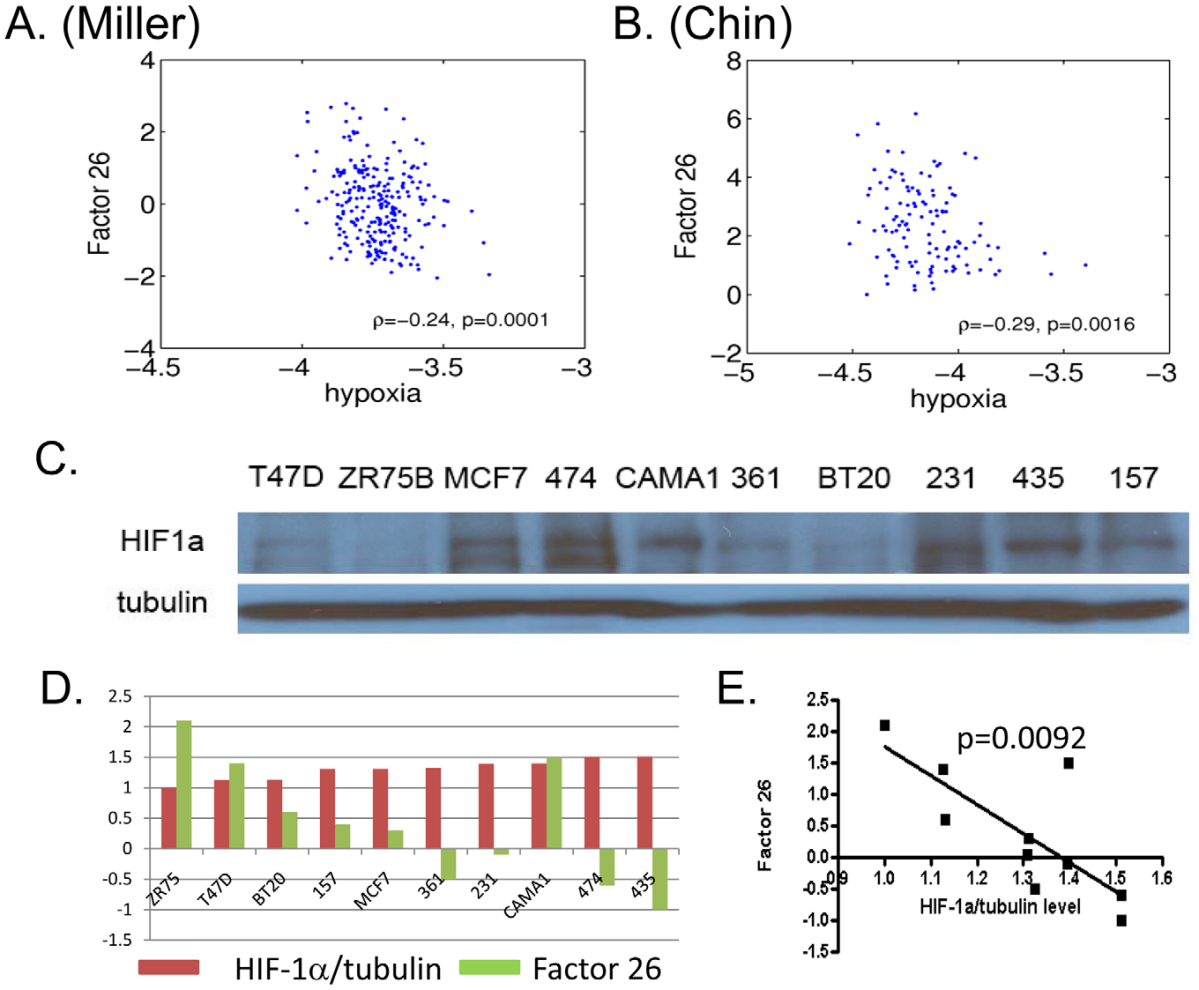
The inverse relationship of factor 26 with the hypoxia response and HIF-1α protein levels. The degree of the association between factor 26 (Y-axis) and hypoxia gene signature (X-axis) is shown for the Miller, A, and Chin, B, breast cancer dataset. Panel C shows the level of HIF-1α proteins under ambient air of various indicated breast cancer cell lines are determined by Western blots. These cells are shorted based on the normalized level of HIF-1α protein and correlate with the expression level of factor 26 (panels D and E).

Given that these factors are identified from the hypoxia gene signature, we examined the relationship between these factors and the hypoxia gene signature. Such analysis identifies a significantly negative relationship between factor 26 and the predicted hypoxia pathways in both Miller and Chin breast cancer (Figure 7A, B) [32], [50]. These results suggest that a high level of expression of factor 26 is associated with a lower level of hypoxia response. It is interesting to note that the breast tumors labeled as 1q/16q CNA based on cluster analysis of array CGH data in the Chin data have a more favorable clinical outcome [32]. Given that a strong hypoxia signature is associated with poor clinical outcome [3], [25], the link between 1q CNA and low hypoxia response is also consistent with better clinical outcomes. Therefore, we postulate that a high expression level of factor 26 in human cancer is probably associated with 1q CNA and significantly negatively associated with hypoxia pathways in human breast cancer (Figure 7A, B).

Among the genes contained in the factor 26, there is one known negative regulators of HIF-1α proteins – Egl Nine Homolog 1 (EGLN1 or PHD2). The high levels of EGLN1 are known to suppress HIF-1α transcriptional activity [59] since it encodes a protein that catalyzes the post-translational formation of 4-hydroxyproline of the proline residues of HIF-1α and targets it for degradation via the von Hippel-Lindau ubiquitylation complex. Additionally, fumarate hydratase (FH) encodes a protein that is an enzymatic component of the tricarboxylic acid (TCA) cycle and its deactivating mutations lead to high level of HIF-1α and tumor formation [60]. Thus, the high expression level of EGLN1 (and maybe FH) in factor 26 due to local segmental aneuploidy may contribute to lower HIF-1α activities. When the HIF-1α protein level in the several breast cancer cell lines was cultured under ambient air, we found that variation in HIF-1α protein levels were inversely correlated with factor 26 expression (p = 0.0092) in a group of breast cancer cell lines [50] (Figure 7C–E). This result is consistent with a previous study [61] showing that MDA-MB-435S and MDA-MB-231 (with low level of factor 26) having a high glycolytic phenotype with elevated HIF-1α protein and glucose uptake. In contrast, MCF-7 has a relatively high factor 26 activity and is found to be lacking glycolytic phenotypes [61]. Taken together; these data suggest that CNAs in 1q leads to the variation in the expression of factor 26, thus modulating the hypoxia pathway and aerobic glycolytic phenotypes. These results highlight the potential of our approach to elucidate the mechanisms underlying the hypoxia pathway and the aerobic glycolytic phenotypes of human cancers.

## Discussion

### The role of DNA copy number alterations in the gene expression

Cancer genome aneuploidies, characterized by mutations, duplications and deletions, play an important role in oncogenesis. Various microarray technologies have been used to perform genome-wide investigations of copy-number changes through array CGH [62], [63] to identify many putative oncogenes and tumor suppressor genes. These changes in DNA copy number or gene dosage often lead directly to changes in expression levels of the RNAs for the relevant genes (*cis*-covariations) or indirectly other genes outside of DNA CNAs (potential *trans*-covariations). These changes in gene expression may contribute to transformation and progression along with oncogenic processes. Therefore, correlation between chromosomal abnormalities and gene expression levels may provide a powerful means to identify tumor aneuploidies.

Although array CGH is a powerful tool for documenting DNA copy number on the genomic landscape, the routine application of array CGH to every tumor tissue used in gene expression studies is not feasible. Theoretically, candidate segmental aneuploidy in human cancers can be detected based on the spatial bias of gene expression without the need for the actual array CGH microarray data. For example, a previous study using gene expression to identify segmental aneuploidy had identified MTDH as candidate oncogene in 8q22 amplified (factor 35) in a small region in breast cancers [54]. In addition, it is still challenging to identify functionally relevant aneuploidy with confidence as well as the insight into the biological mechanism and clinical relevance of CNAs. In addition, these methods may identify only the *cis*-covariations in which the variations in gene expression are physically located on the chromosomal regions with segmental aneuploidy. It will be difficult to identify *trans*-covariations as illustrated in the involvement of CNAs in the gene expression of wound signature [31].

Our study, presented in this manuscript, presents a novel approach – the use of sparse factor analysis to identify CNAs in human cancers which are associated with biological processes as captured in the *in vitro* derived gene signatures. We reason that the CNAs may lead to the coordinated expression of genes within these (*cis*-covariations) or other distant chromosomal regions (*trans*-covariations), and thus identifiable as factor components across different human cancer datasets. Since this method is initiated by pathway-specific gene signatures, which can later be linked to latent factors and then CNAs in specific chromosomal regions with high confidence, the discovered association can better enable us to gain biological insights into these pathway-associated CNAs to enable better development of hypotheses in terms of the causes and consequences. Finally, the overall approach provides a generic framework for the joint analysis of disparate data sets and for the generation of hypotheses based on data collected at many different experimental systems and in various biological contexts.

### The advantages and limitations of factor analysis to discover CNAs in human cancers

In the effort to identify the CNAs from array CGH data, it is sometimes difficult to draw the most appropriate filtering criteria. This issue is especially exacerbated by the issue of variations in the degree of DNA aneuploidies among different tumors and cancer cells, the contaminating normal cells and the differing spatial resolution for the microarray formats for array CGH. For example, the large sizes of the BAC clones make it especially challenging to distinguish authentic signals from noise. Our alternative approach, starting from gene expression data, is likely to identify those CNAs which are functionally relevant in terms of significant variation in gene expression. For example, many segmental DNA aneuploidies found in our analysis may not have been previously recognized with confidence due to their modest and inconsistent deviation from the array CGH. But their variations have led to significant and consistent changes in the expression of the genes which allows their confident recognition using our approach. Thus, latent factors may present an alternative and complimentary approach to identifying DNA aneuploidies which are associated with particular biological processes.

Since this approach focuses on gene expression, the identified segmental aneuploidies may agree or disagree with the quantitative analysis from the measurement of DNA copy number. For example, our approach has identified many high copy amplification regions found in the original study of array CGH in breast cancers. However, in addition we have identified several additional CNAs with confidence. The identification of these factors from the latent factor model indicate that our approach may help to incorporate coordinated gene expression patterns which leads to a higher confidence in the interpretation of CNA than that derived from the use of BAC data alone or the incorporation of gene expression data from single genes. This is especially of value in instances of low level amplification. There are also several CNAs which we did not discover through our factor analysis, such as the recurrent CNA in 11q13, 21q22 and 17q12. This may be due to the fact some of these CNAs do not lead to significant gene expression in the factors or their effects on the gene expression are not significantly correlated with the hypoxia or lactic acidosis.

### Examples for the improved understanding from the factor analysis of gene signatures

The use of factor analysis to dissect the original gene signatures has shown its utility in several biological contexts. The combination of signature-derived latent factors has been shown to improve the prognostic value and predictive power of the original hypoxia and lactic acidosis signatures [26]. A recent analysis of the prognostic factors in breast cancer has highlighted the importance of a glycolytic enzyme PGK1 and tumor glycolysis in the prognostic value of stress signatures [64]. In addition, factor analysis has allowed the molecular dissection of the Ras pathways into individual components [14]. In this study, we have further identified the potential role of 1q CNA in the degree of hypoxia response in the breast cancers. This finding has suggested a role of the gene dosage of two HIF-1α negative regulators (ELGN1 and FH) as determinant of tumor hypoxia responses as discussed below.

### The link of CNAs to the variations in hypoxia/lactic acidosis response in human cancers

There are three reasonable possibilities; variation in the hypoxia response program might reflect: (1) actual variations in oxygen tension in the tumors; (2) cell type-specific variations in the magnitude of, or threshold for, the response to bona fide hypoxia, similar to those seen in our analysis of different normal cells; or (3) inappropriate activation of the hypoxia response resulting from genetic and/or epigenetic alterations in cancers. Although the hypoxia response in tumors is usually thought to be caused by the first mechanism, evidence suggests contributions from the second and third mechanisms as well. For example, activation of the hypoxia response program in clear-cell RCC is almost certainly caused by loss of VHL [4] rather than by low oxygen tension. In breast cancer, the over-representation of p53 loss-of-function mutations in the tumors with elevated hypoxia responses suggests that the loss of the p53's role in inhibiting HIF-1α protein stability and hypoxia-induced cell death [4],[55]–[57] may be a factor in these tumors. Other oncogenic alterations in regulatory systems [3]–[7] might also play a role in triggering or modifying the hypoxia response in human cancers; a dissection of the contributions of tumor oxygen levels and disordered regulation of the hypoxia response in individual tumors will, therefore, be important in developing therapeutic strategies based on exploitation or inhibition of this program. Our results clearly demonstrate that a significant amount of tumor CNAs are associated with the hypoxia and lactic acidosis responses in human cancers and these CNAs may contribute to variations in the hypoxia and lactic acidosis gene sigantures. For example, the amplification of 1q (factor 26) is often observed in subsets of breast cancers and is associated with a group of breast cancers with more favorable clinical outcomes [32]. The identification of negative correlation between factor 26 (1q CNA) and the hypoxia response in breast tumor and cell lines may help to explain such observations. Our analysis has pointed out additional associated relationship between particular CNAs and the hypoxia/lactic acidosis signatures to allow the development of hypothesis on the mechanistic basis for such association for experimental validation.

### Tumor microenvironmental stresses as selection pressure for CNAs

It is possible that the hypoxia, lactic acidosis and other tumor microenvironmental stresses play a direct role in selecting for the cancer cells with particular CNA. Such concepts have been suggested by many reviews on the ability of hypoxia and acidosis to select for tumor cells with strong metastasis phenotypes and invasive behaviors [27], [29], [30]. Many of the breast tumor CNAs observed in the Chin studies are also found in the cultured cells undergoing telomere crisis and immortalization [32]. These possibilities are also suggested by the CNAs we have identified to be associated with hypoxia and lactic acidosis. For example we have identified a strong positive correlation between the CNA in 22q11–13 associated with factor 23. Among the genes in this CNA region is ATF4, a master regulator of transcriptional response of unfolding protein response (UPR). It is known that hypoxia stresses lead to the UPR and that the proper transcriptional module of UPR is essential for cellular survival under hypoxia [65], [66]. We hypothesize that amplification of ATF4 and adjacent regions leads to constitutive and strong UPR, conferring a survival advantage for cells under hypoxia stresses.

### Future direction and perspective

The strategy presented in this paper will be developed for multiple sets of factors generated across the range of biological pathways resulting from other microenvironmental stresses and oncogenic signaling events. Further, the availability of both sequencing data and gene expression data for sets of human cancers from the Cancer Genomics Atlas Project will allow further exploration of genetic changes identified as linked to dysregulated expression of these gene signatures in human cancers. In one sense, the dysregulated gene expression can be used as a biological phenotype to help interpret and decipher the consequences of genetic changes of different sizes, either on the amplification/deletions of chromosomal regions of varying sizes or single base mutations in human cancers. Such an integrative genomic approach is likely to help unravel the enormous complexity of human cancers and allow more precise and personalized therapeutic strategies.

## Methods

### Statistical model specification

In general, for experiments performed on clones under strictly controlled conditions, such as that, our models utilize multivariate regression and analysis of variance with a fixed known design to describe observed expression patterns. Suppose that we have *p* isotope groups and *n* samples. We define *X* to be the *p*×*n* matrix of log-intensity values with elements *x_g,i_*. Let *H* be the *r*×*n* matrix, with elements *h_j,I_*, whose columns consist of the *r* known design vectors. We model the measured expression values as:
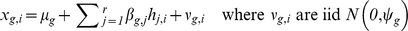
or, in matrix form,
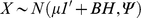
where *1'* is the *n*-dimensional column vector of 1's and *Ψ = diag(ψ_1∶g_)*.

Depending on the experimental context, the rows of *H* may include entries reflecting treatment effects, environmental interventions, or clinical variables for which we want to control.

### Sparsity priors

Because we are dealing with very high dimensional genomic assays, we expect that most genes (probe sets) will not show differential expression in relation to a particular design vector. Mathematically, this statement is equivalent to the assumption that the matrix *B* will be sparse (for most *i* and *g* we will have *β_g,i_ = 0*). We reflect this assumption with the standard point mass mixture distribution on the coefficients *β_g,i_*:
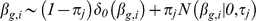
Our assumption is that the probability of any particular probe associating with a given design vector is quite low, so we assign *π* a low mean Beta prior. This structure leads to models for intensity values that are as parsimonious as possible while still identifying isotope groups that are related to the design vectors.

### Latent factors

We have previously performed genomic analysis of Human Mammary Epithelial Cells (HMEC) which have been exposed to lactate, acidosis and combined lactic acidosis for 24 hours (GEO accession number GSE9649) [25]. We fit the sparse regression model described above to this data, and from this analysis, we selected a set of 2984 genes based on the posterior probability of differential expression – the posterior distribution for π – for any of experimental groups being greater than .99.

The latent factors model as described thus far assumes that all sources of variation in intensity are known and represented by a fixed design. An important feature of the modeling formulation is the ability to account for unknown sources of variation such as the activity of various biological pathways. These sources would be reflected in common intensity patterns across multiple subsets of isotope groups, and will be described by the identification of various factors.

Extending the model to include factors can be expressed as
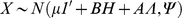
Where the *k×n* matrix *Λ* represents the realized values of *k* latent factors across the *n* samples, having elements λ_j,i_ for factor j = 1∶k on sample i = 1∶n. *Λ* is an analogue to the known matrix *H* (though it contains unknown vectors to be learned). The columns of the *p×k* factor loadings matrix *A = α_g,j_* are the coefficients of isotope groups on factors (these are analogous to regression coefficients in *B*.

We again make use of sparsity priors for the elements of *A* (the same as those used for *β*). The elements of *Λ* may be given standard normal prior distributions. The properties of this model have been fully laid out fully in [3] along with details of implementing the Markov Chain Monte Carlo (MCMC) algorithm for fitting the parameters. Details on the use of this model in the setting of predicting factor scores and pathway activity are laid out in [1]. We make use of software which implements the MCMC algorithm of [3] and is freely available to the public. The details of using this software are described in [2].

We fit this factor model to the 251 samples from the Miller data set and the 2984 genes from [25], resulting in the 56 factors that we have described herein.

### Enrichment in gene ontology and chromosomal locations among the factors genes

Gather is a fully developed software product described [40]. To assess enrichment for specific lists of probe sets, we simply drop the list into the web based interface and all associations are tested and reported.

### Local enrichment ratio

We note that one of the features of the Bayesian sparse latent factor model that we use is the estimation of posterior probabilities for each gene and each factor, π*_g,j_. This parameter is interpreted to be the posterior probability that gene *g* influences factor *j* or alternatively the posterior probability that factor *j* is important for describing the variation observed across samples for gene *g*. Now, given a list of posterior probabilities for factor *j* we are faced with the challenge of assessing the extent to which the genes with high posterior probability cluster in a specific region of the genome. We treat each factor independently, therefore for the purposes of reducing notational complexity, we will drop the factor index, *j*.

Define *x_g_* to be the start location of a gene observed to be in the factor and let *κ_g_(t)* be a kernel function associated with genome location *x_g_*. Because we know that CNA can occur across regions of the genome that are relatively large compared to the length of a gene, we approximate gene location with a point mass at its start site. Using this approximation, we define *κ_h_(t)* to be a discretized normal distribution with mean *h* and variance *τ^2^*, truncated at the edge of the chromosome on which *h* resides and properly scaled (to add to 1). This function serves to distribute a gene observed to be in the factor across a large set of locations across the genome.

We define the local enrichment ratio at a point *t* along the genome as
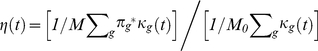
Where *M* and *M_0_* are integration constants, such that M = ∑_g_∑_t_π_g_^*^κ_g_(t) and M_0_ = ∑_g_∑_t_κ_g_(t). This is a ratio of kernel smoothed empirical distributions similar to that developed in [49] and many other places. Kernel smoothers have a long history, including their use in likelihood ratio tests. Some theory on the properties of the ratios of kernel smoothed empirical distributions has been worked out in [67] and their use in the context of partial linear regression is described in [68]. However, literature on their use in the context of gene expression data is minimal and their application to posterior latent factor inclusion probabilities is novel. For the purposes of this analysis, we are analyzing a set of 2984 genes, and this will limit our resolution to regions that are significantly larger than the length of a gene. In general, the appropriate kernel width (τ^2^) will depend on the number of genes in the gene lists (with a longer list of possible genes allowing for a smaller sandard deviation in the kernel). For this paper, we utilize a standard deviation of approximately 5% of the length of a chromosome. However, testing across a wide range of sizes demonstrates a relative insensitivity to this parameter (Figure S3).

In order to estimate an appropriate significance level for the local enrichment ratio for a particular factor, we simulate from the null distribution by permuting the posterior probabilities. Thus, if *σ(g)* is a permutation, then a sample LER from the null hypothesis is generated as follows:
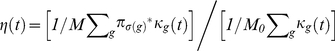
Repeated simulations, using multiple different permutations across all factors demonstrates that a significant LER varies according to the window size of the kernel. However, at a kernel width that is 5% the size of a chromosome, the probability of a maximum LER>3 across the entire genome is less than 1/10,000. Similar significance levels for kernel widths of .5%, 2%, 8% and 11% are 2.3, 2.6, 4.5 and 6.

### The projections of factors into other datasets to investigate relationship with CNA

In order to assess the relationship between derived latent factors and interventions/variables from other experiments, we need to be able to estimate *Λ*, given *A*, in new data sets. That is, we have a new data set, *Y*, and we wish to estimate the factor scores on this data set, *Λ_y_*. This is a well known problem of inverse regression; however, because we are in the situation of very high dimensional data, we know that *A'A* will not be invertible. This means that we cannot simply apply the least squares estimator, *Λ_y_ = (A'V^−1^A)^−1^A'V Y*. We address this issue by utilizing a Bayesian framework for the estimation of *Λ_y_*. In particular, we use the prior distribution
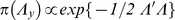
This is a simplified version of a more general prior described in [36]. Then if *A* is the matrix of factor loadings (regression coefficients) from model fitting and *V* is a diagonal matrix containing variances for each gene, we estimate
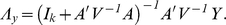
(2)(In this equation, *I_k_* is a *k*-dimensional identity matrix.) The derivation of this posterior from the prior and likelihood are discussed in both [36] and [69]. Goldstein et al. also discusses the relationship of this estimator to ridge regression estimators [69].

The use of this solution to the inverse regression problem allows us to build a factor model on any data set and project those factors onto any other data set in which we have measurements of all of the relevant probe sets. To test association between factors and CGH, we project the factors discovered in [37] onto the data sets from [32] and [50]. We then compare the 56 factor vectors to the BAC clones from these data sets using Pearson correlation. This is the same approach taken to project our factors onto the cell line data from [25].

### Estimation of the statistical significance of conserved coordinate expression

We are interested in estimating whether a particular collection of genes, showing coexpression in one data set, shows the same type of coexpression patterns in a new data set. In essence, we want to quantify the level of similarity between Figure 3A and Figures 3B, C, D and E. It is first important to understand how these figures were generated.

Note that, in this context, neither samples nor genes have a canonical order, thus we may reorder both the rows and columns of these heatmaps in order to make coherent expression more clear. Thus, for visualization, we compute the first principal component, *u_1_*, of the data matrix. Define *ρ_j_* to be the correlation between *u_1_* and row *j* of the data matrix and ***ρ*** to be the vector of those correlations. Define |*ρ*|^+^ to be the number of rows, *j*, in the data matrix for which *ρ_j_>0* and |*ρ*|^−^ to be the number of rows for which *ρ_j_<0*. We choose the sign of *u_1_* so that |*ρ*|^+^>|*ρ*|^−^. The columns are then sorted so that *u_1_* is decreasing and the rows are sorted so that ***ρ*** is decreasing.

One of the challenges inherent in assessing the extent to which a particular expression pattern is conserved is due to the fact that the samples are completely different. We note that the procedure outlined in the previous paragraph can be used to sort a collection of genes based on coexpression, and that it is entirely internal to the data matrix. Thus, given a fixed set of genes and two completely independent samples, we may use each data set to perform this sorting. The rows of the five data matricies depicted Figure 3 are all sorted according ***ρ_miller_*** in order to enhance interpretability.

We test the significance of conserved coordinate expression by computing the Kendall correlation of two different methods of sorting. Thus, from Figure 3, the Kendall correlation of ***ρ*** *_miller_* and ***ρ*** *_gray_* is .67, and the p-value associated with this correlation is 2.2×10^−15^. Note that, by default, the computation of the Kendall correlation statistic involves first replacing the elements of ***ρ*** *_miller_* and ***ρ*** *_gray_* with their ranks. Thus, this is a non-parametric, rank based correlation. Scatterplots of the ranks, comparing Miller to each of Gray, Nevins, Berchuck and Freije are shown in Figure S5.

### The relationship between the factor expression and hypoxia and lactic acidosis signatures

This equation described above is used to project both signatures and factors – using *B* instead of *A* in the case of signatures. Thus to assess both signature and factor expression levels on an *n*-dimensional data set, one first projects the relevant factors and signatures, then compares the obtained *n*-dimensional vectors to each other with whatever test is relevant. In this paper, we use Pearson correlation to assess significance of association. The procedure for comparing signatures to array CGH is, thus, exactly analogous to that used for comparing factors to array CGH.

### HIF-1α proteins and association with factor 26

The indicated breast cancer cell lines were cultured under ambient air, lysed and supplemented with protease inhibitor cocktail (Roche Applied Science) followed by sonication and cold centrifugation. Equal volumes of sample buffer were added to 20 ug of proteins, boiled, resolved on a 10% Tris-HCl gel (Bio-Rad), and transferred onto polyvinylidene fluoride (PVDF) membrane (Hybond-P, GE Healthcare). HIF-1α protein was detected using anti-HIF-1α monoclonal antibody (cell signaling) followed by horseradish peroxidase (HRP)-conjugated goat anti-rabbit IgG antibody (Abcam). The western blot was then visualized by enhanced chemoluminoscence (Western Lightning-ECL Plus, PerkinElmer) and exposure to film. Images were digitized by scanner. To control for protein loading, the membranes were stripped (Restore Plus, Thermo Scientific) and reprobed with goat anti-rabbit antibody to beta-tubulin (Abcam). Densitometric measurements were performed with ImageJ software (http://rsb.info.nih.gov/ij/). The correlation between the normalized HIF-1α with tubulin signals with factor 26 was calculated by GraphPAD Prisms.

## Supporting Information

Figure S1 The coordinated expression of the latent factors in the five indicated cancer datasets of breast, lung, ovarian and brain cancers.
(5.76 MB ZIP)

Figure S2 The local enrichment ratios (LER) for chromosomal enrichment of the latent factors.
(0.86 MB ZIP)

Figure S3 The local enrichment ratios (LER) for chromosomal enrichment of the latent factor 26 using different amount of Kernel widths.
(0.25 MB TIF)

Figure S4 The association of the BAC clones with the expression of CNA-associated factors.
(2.15 MB ZIP)

Figure S5 Scatterplots comparing the ranking of genes in factor 26 as computed with each of the 5 different data sets. High levels of correlation indicate a factor that is conserved between the two data sets.
(0.09 MB TIF)

Table S1 The gather analysis of the GO enrichments and enrichment for all in their chromosomal locations based on gather, local enrichment ratio (LER) and association with BAC clones for the 56 latent factors.
(0.03 MB XLS)

Table S2 Full gather analysis of chromosome location for each factor.
(0.08 MB ZIP)

Table S3 Full gather analysis of gene ontology for each factor.
(0.43 MB ZIP)

Text S1 Statistical supplement - matlab code and parameter files for duplicating results.
(10.99 MB ZIP)

Text S2 The lists of the probsets in each of the latent factors.
(0.02 MB ZIP)

Text S3 The lists of the BAC clones associated with expression of CNA-associated factors.
(0.01 MB ZIP)
