# Supplementary figures and images for: Latent Factor Analysis to Discover Pathway-Associated Putative Segmental Aneuploidies in Human Cancers (part 1 of 2)
Source: PLoS Comput Biol. 2010 Sep 2;6(9):e1000920. doi: 10.1371/journal.pcbi.1000920 (PMC2932681; doi:10.1371/journal.pcbi.1000920)

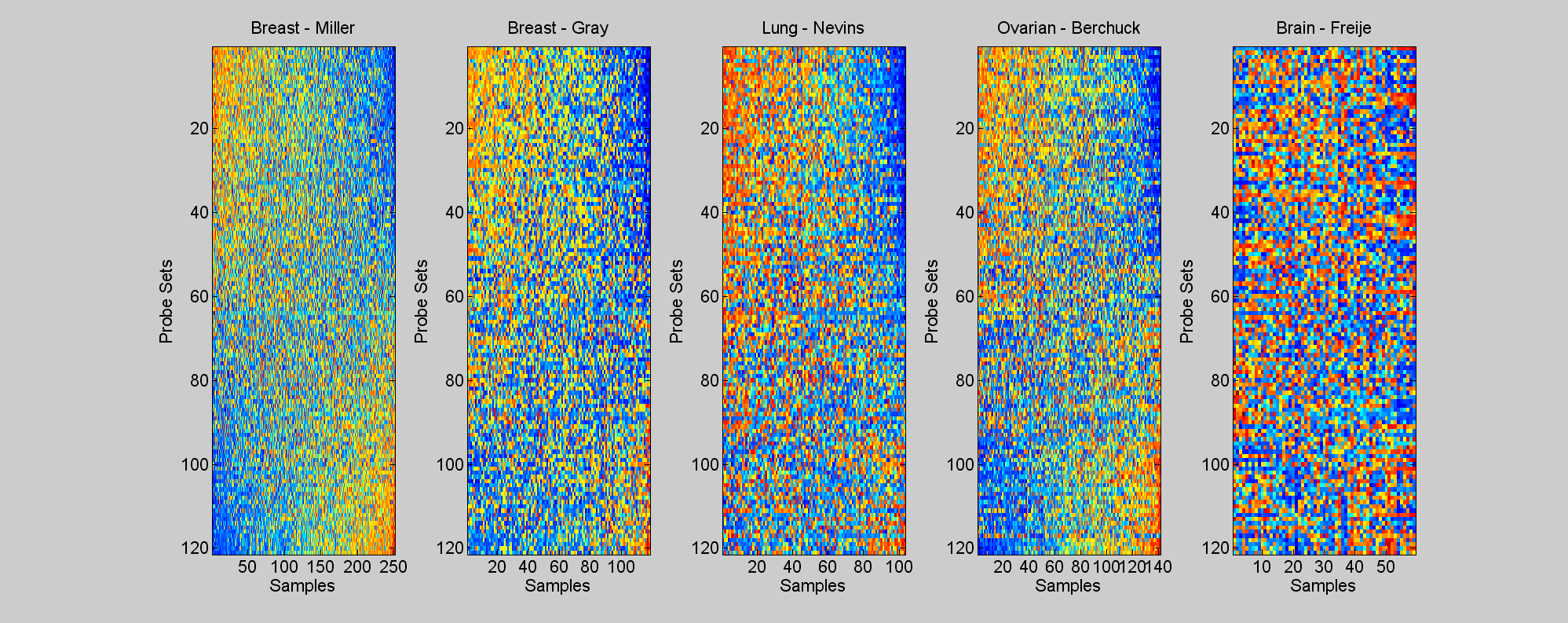

Supplement: Figure S1 — The coordinated expression of the latent factors in the five indicated cancer datasets of breast, lung, ovarian and brain cancers. (5.76 MB ZIP) [file pcbi.1000920.s001.zip › fac19.png]

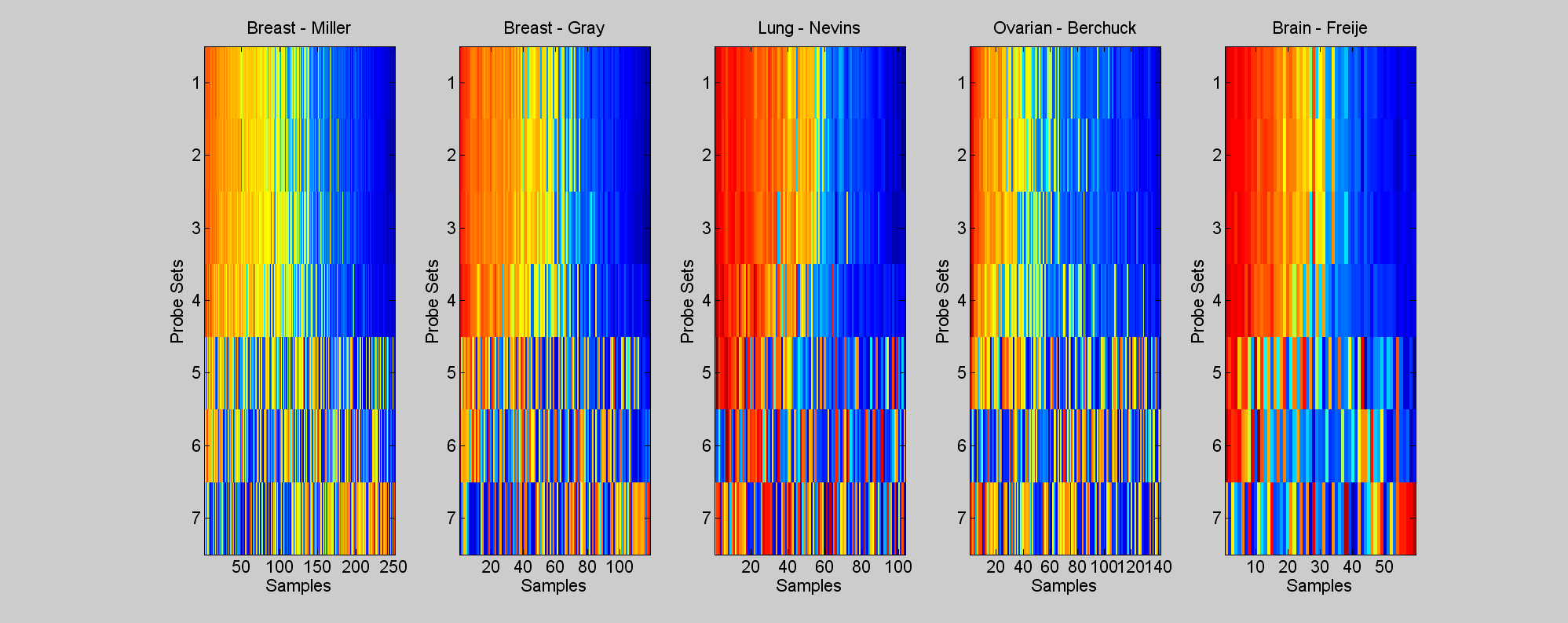

Supplement: Figure S1 — The coordinated expression of the latent factors in the five indicated cancer datasets of breast, lung, ovarian and brain cancers. (5.76 MB ZIP) [file pcbi.1000920.s001.zip › fac20.png]

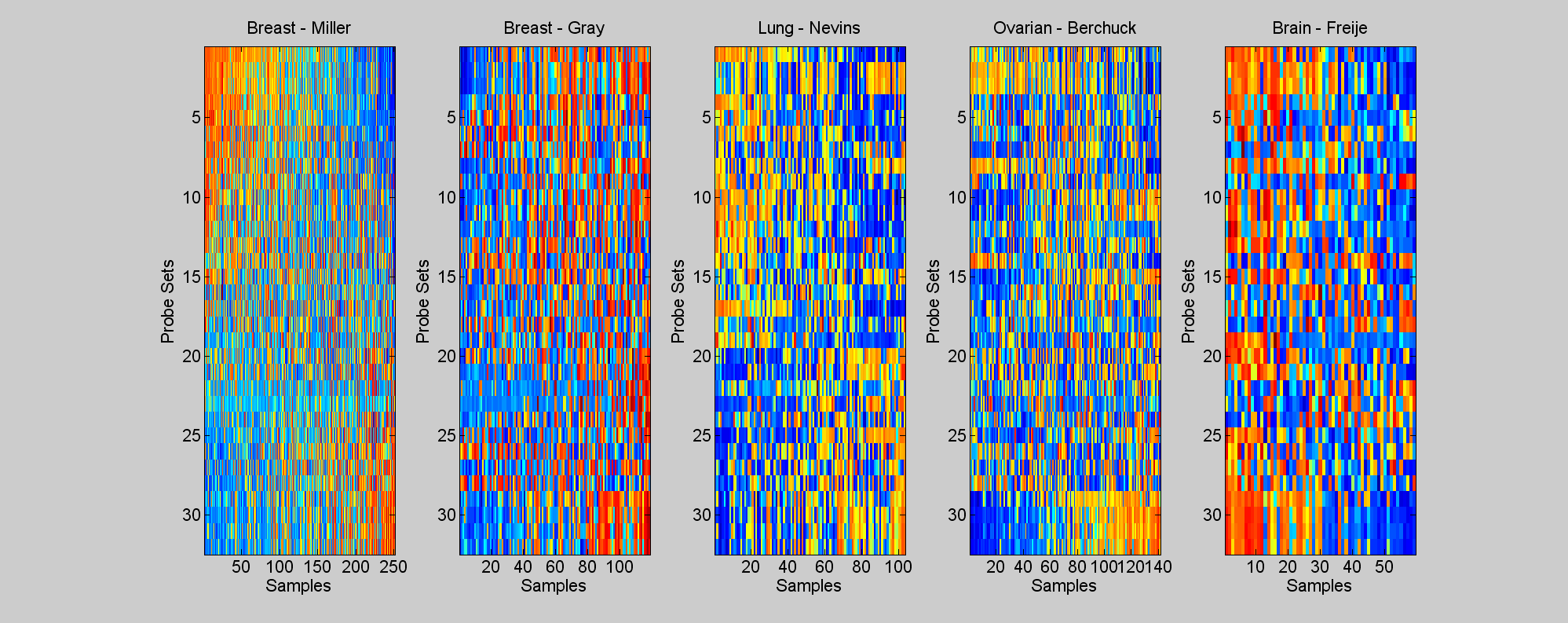

Supplement: Figure S1 — The coordinated expression of the latent factors in the five indicated cancer datasets of breast, lung, ovarian and brain cancers. (5.76 MB ZIP) [file pcbi.1000920.s001.zip › fac21.png]

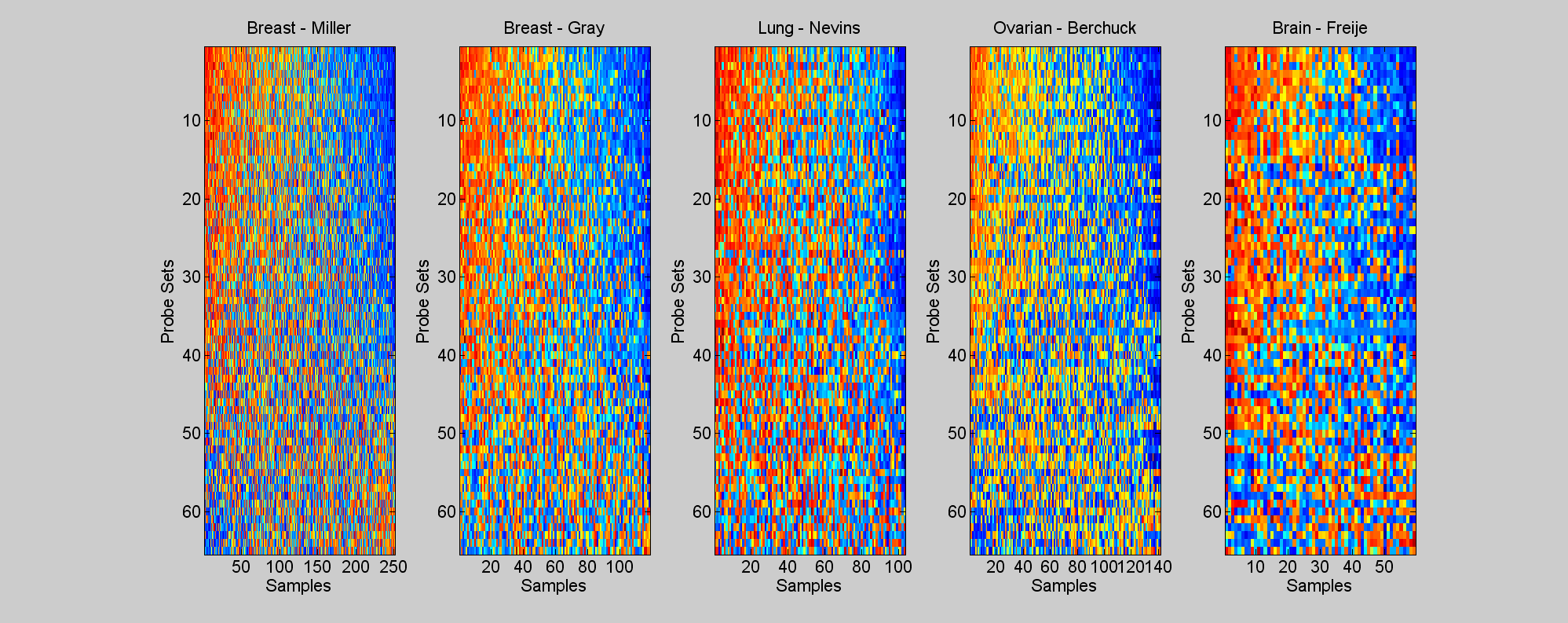

Supplement: Figure S1 — The coordinated expression of the latent factors in the five indicated cancer datasets of breast, lung, ovarian and brain cancers. (5.76 MB ZIP) [file pcbi.1000920.s001.zip › fac22.png]

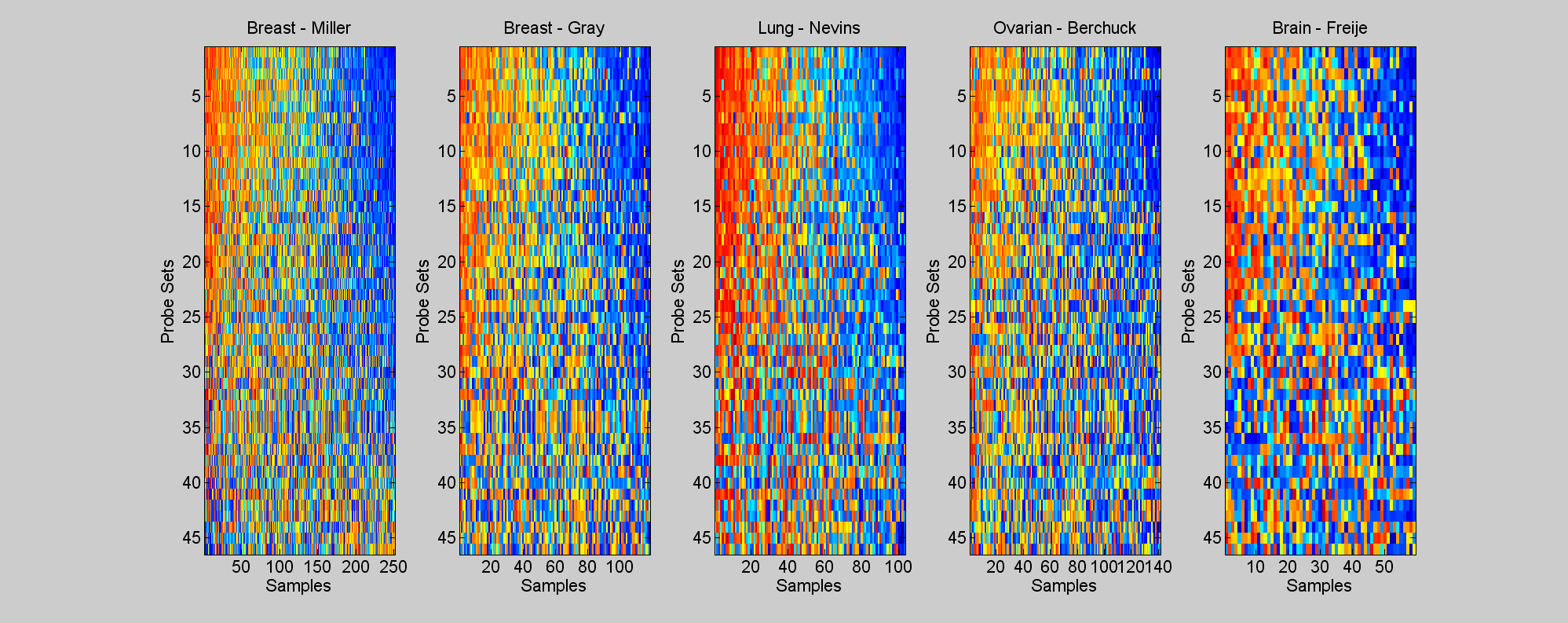

Supplement: Figure S1 — The coordinated expression of the latent factors in the five indicated cancer datasets of breast, lung, ovarian and brain cancers. (5.76 MB ZIP) [file pcbi.1000920.s001.zip › fac23.png]

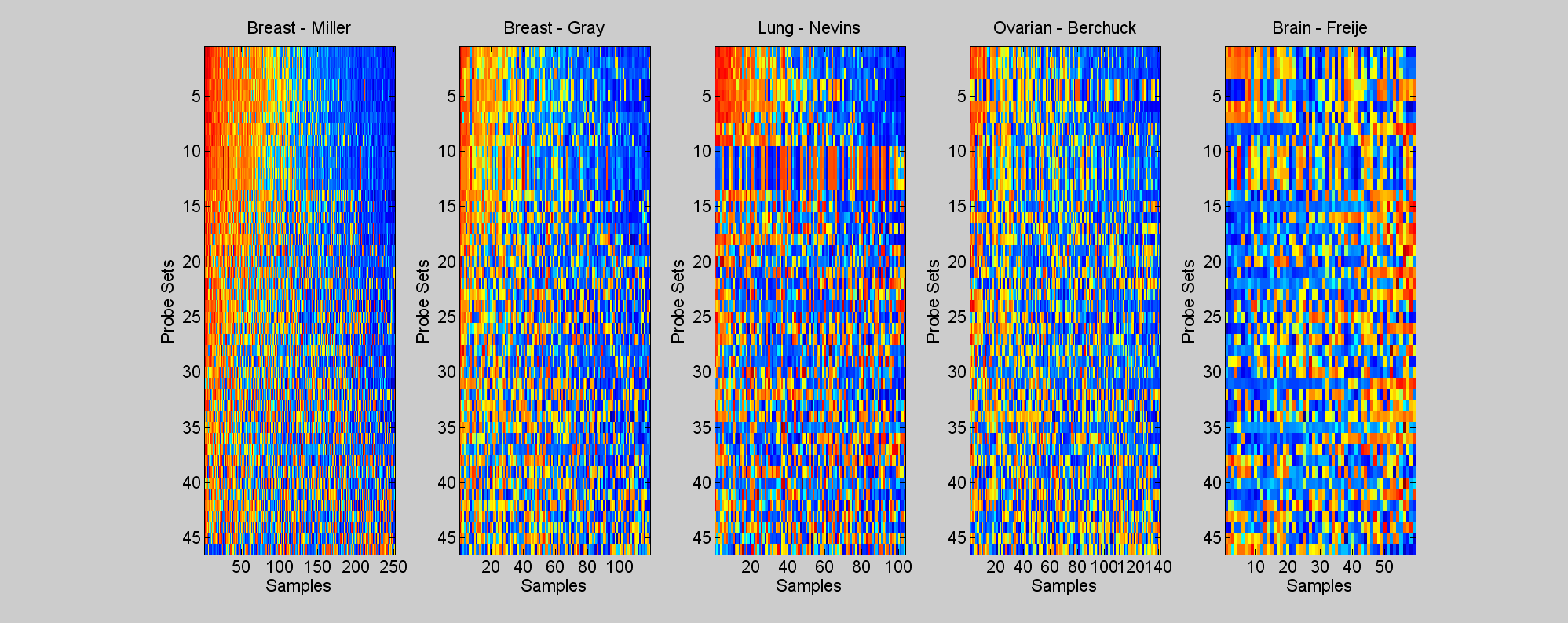

Supplement: Figure S1 — The coordinated expression of the latent factors in the five indicated cancer datasets of breast, lung, ovarian and brain cancers. (5.76 MB ZIP) [file pcbi.1000920.s001.zip › fac24.png]

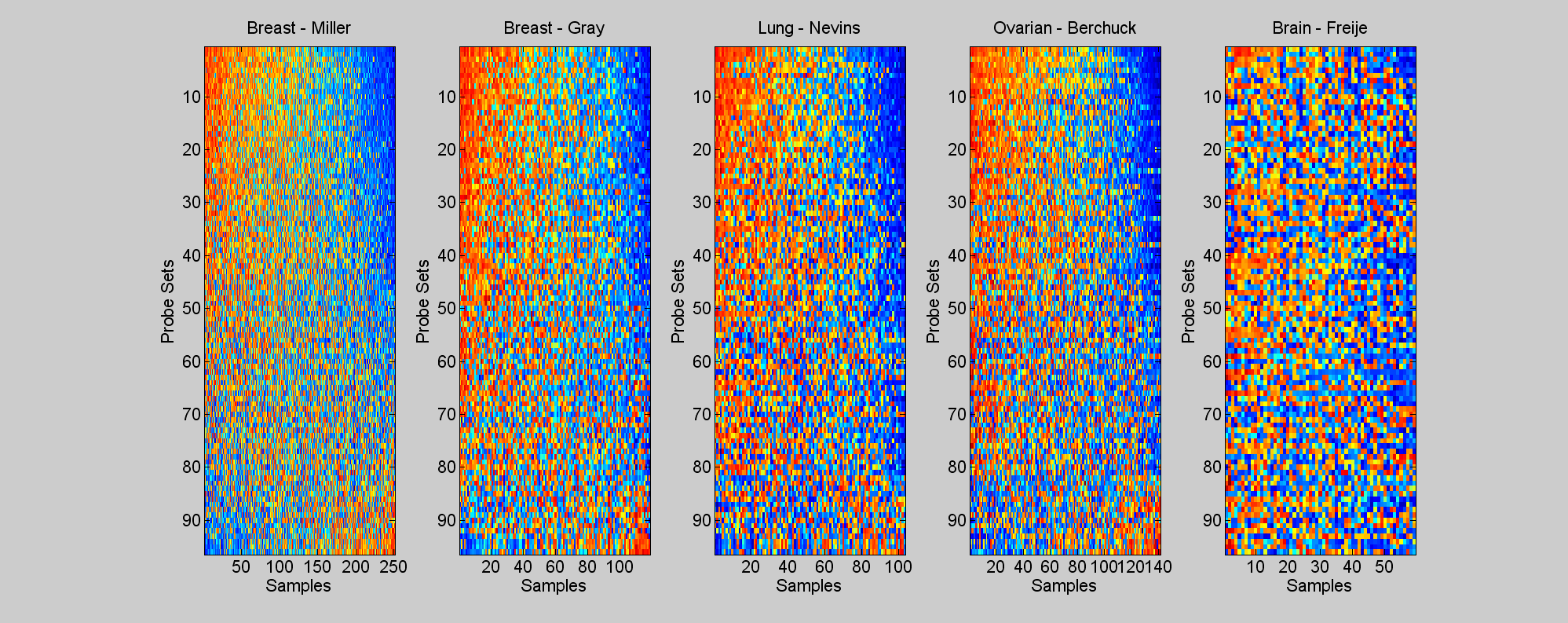

Supplement: Figure S1 — The coordinated expression of the latent factors in the five indicated cancer datasets of breast, lung, ovarian and brain cancers. (5.76 MB ZIP) [file pcbi.1000920.s001.zip › fac25.png]

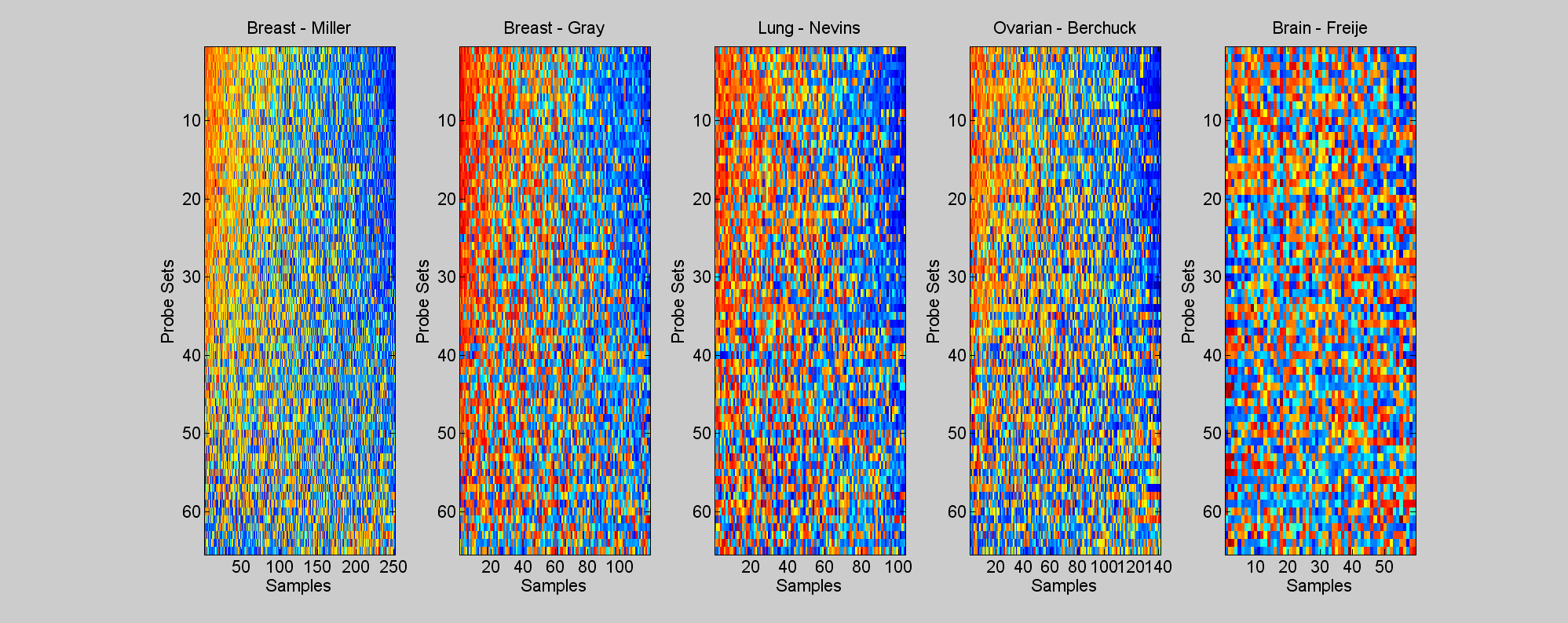

Supplement: Figure S1 — The coordinated expression of the latent factors in the five indicated cancer datasets of breast, lung, ovarian and brain cancers. (5.76 MB ZIP) [file pcbi.1000920.s001.zip › fac26.png]

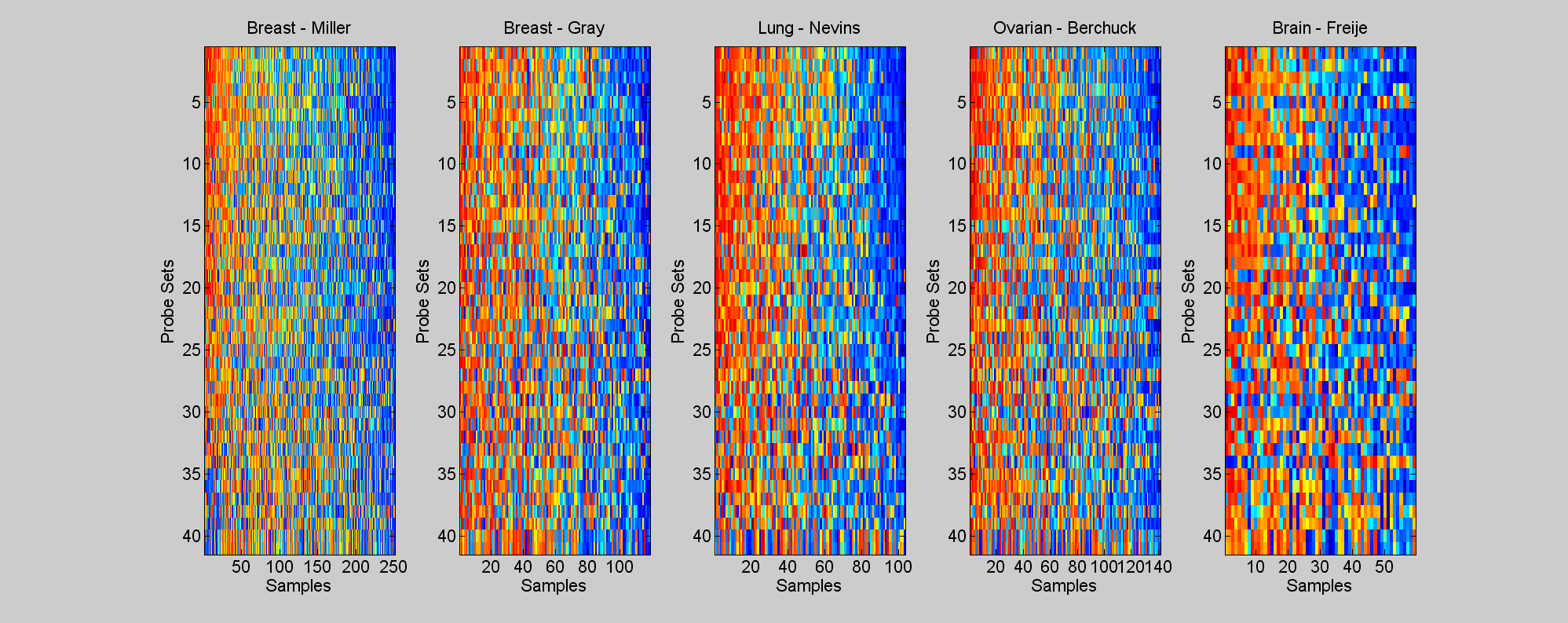

Supplement: Figure S1 — The coordinated expression of the latent factors in the five indicated cancer datasets of breast, lung, ovarian and brain cancers. (5.76 MB ZIP) [file pcbi.1000920.s001.zip › fac27.png]

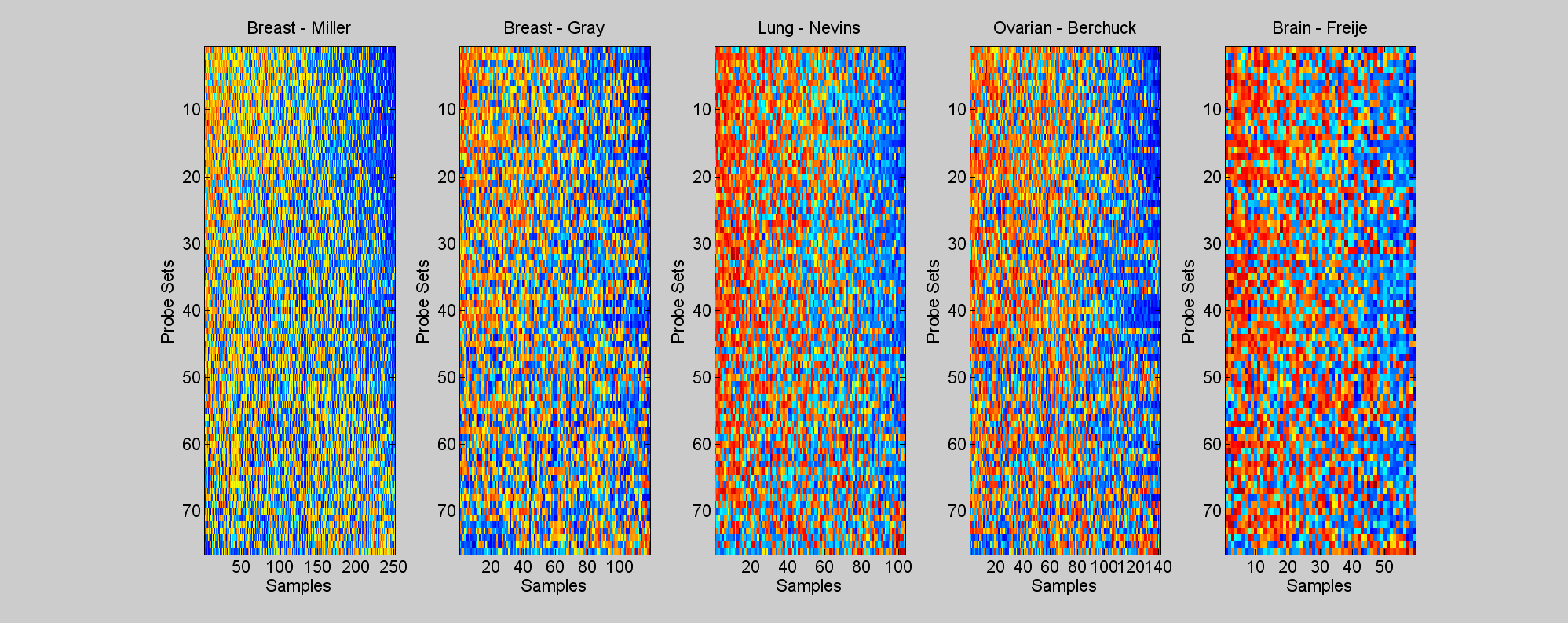

Supplement: Figure S1 — The coordinated expression of the latent factors in the five indicated cancer datasets of breast, lung, ovarian and brain cancers. (5.76 MB ZIP) [file pcbi.1000920.s001.zip › fac28.png]

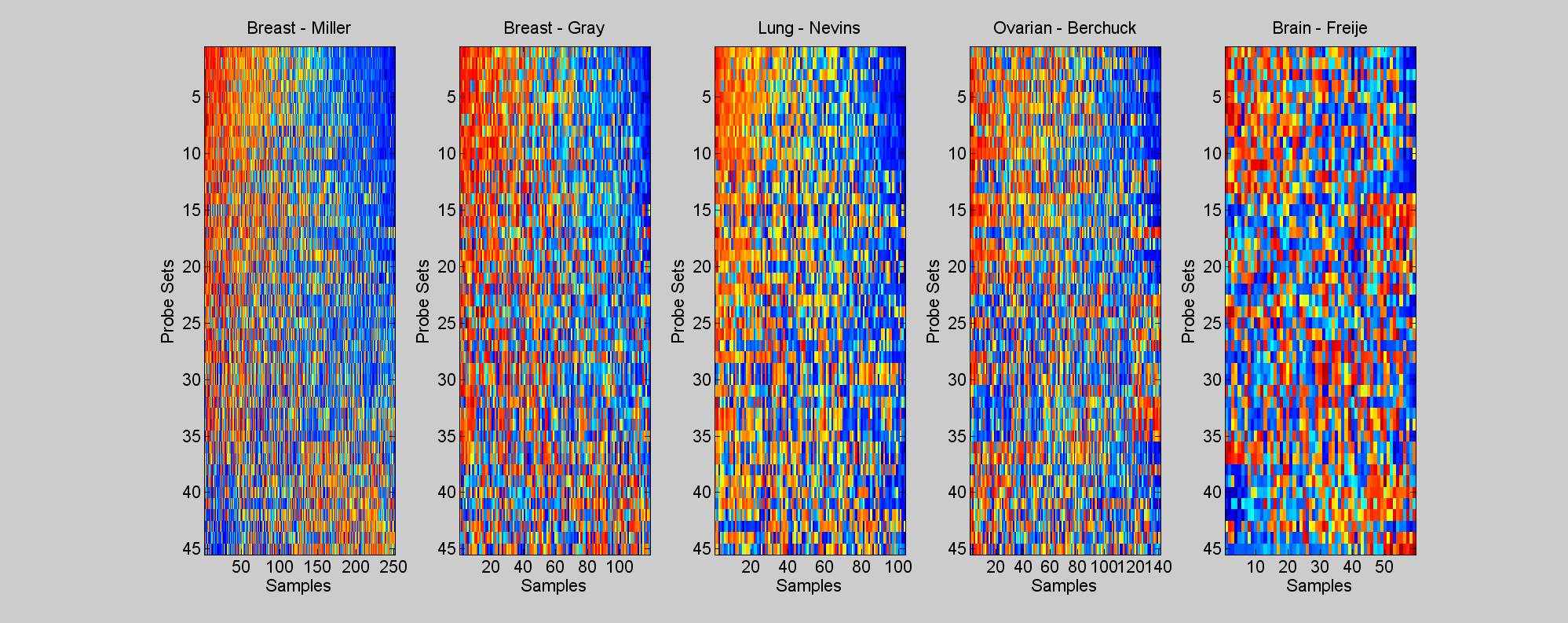

Supplement: Figure S1 — The coordinated expression of the latent factors in the five indicated cancer datasets of breast, lung, ovarian and brain cancers. (5.76 MB ZIP) [file pcbi.1000920.s001.zip › fac29.png]

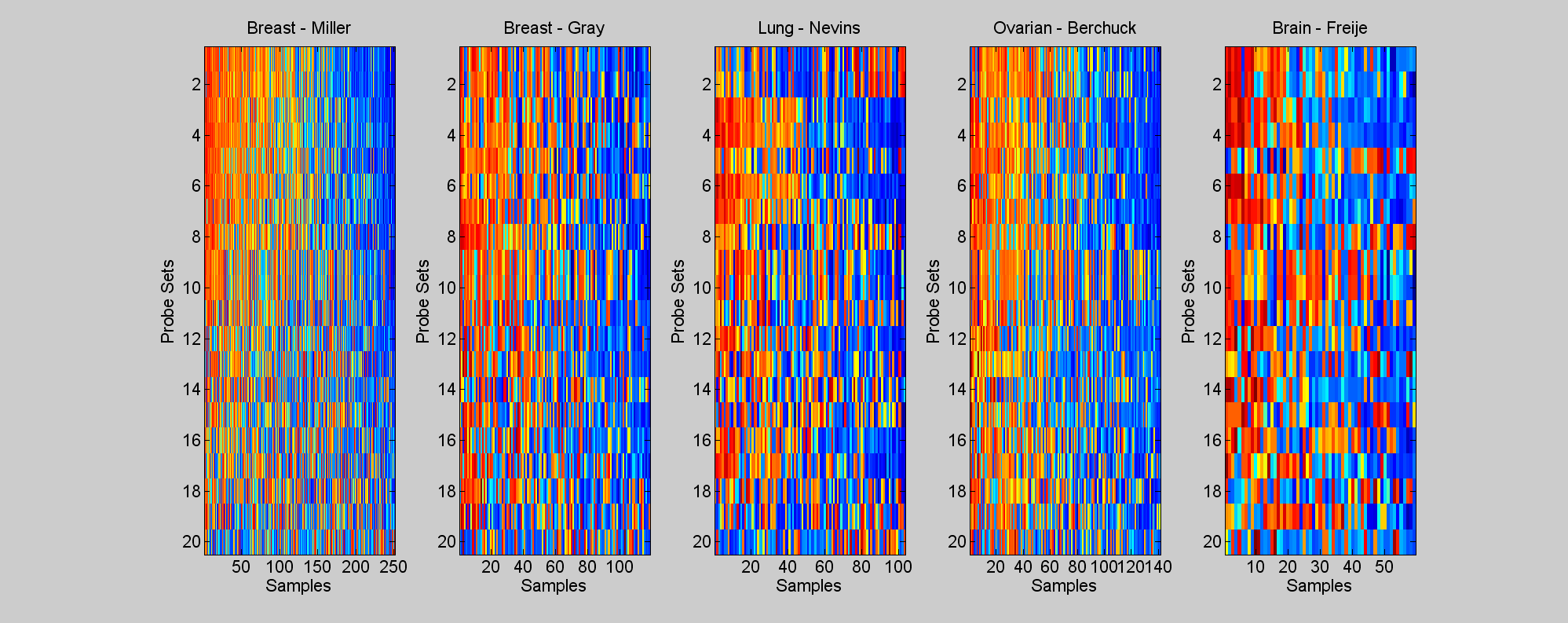

Supplement: Figure S1 — The coordinated expression of the latent factors in the five indicated cancer datasets of breast, lung, ovarian and brain cancers. (5.76 MB ZIP) [file pcbi.1000920.s001.zip › fac30.png]

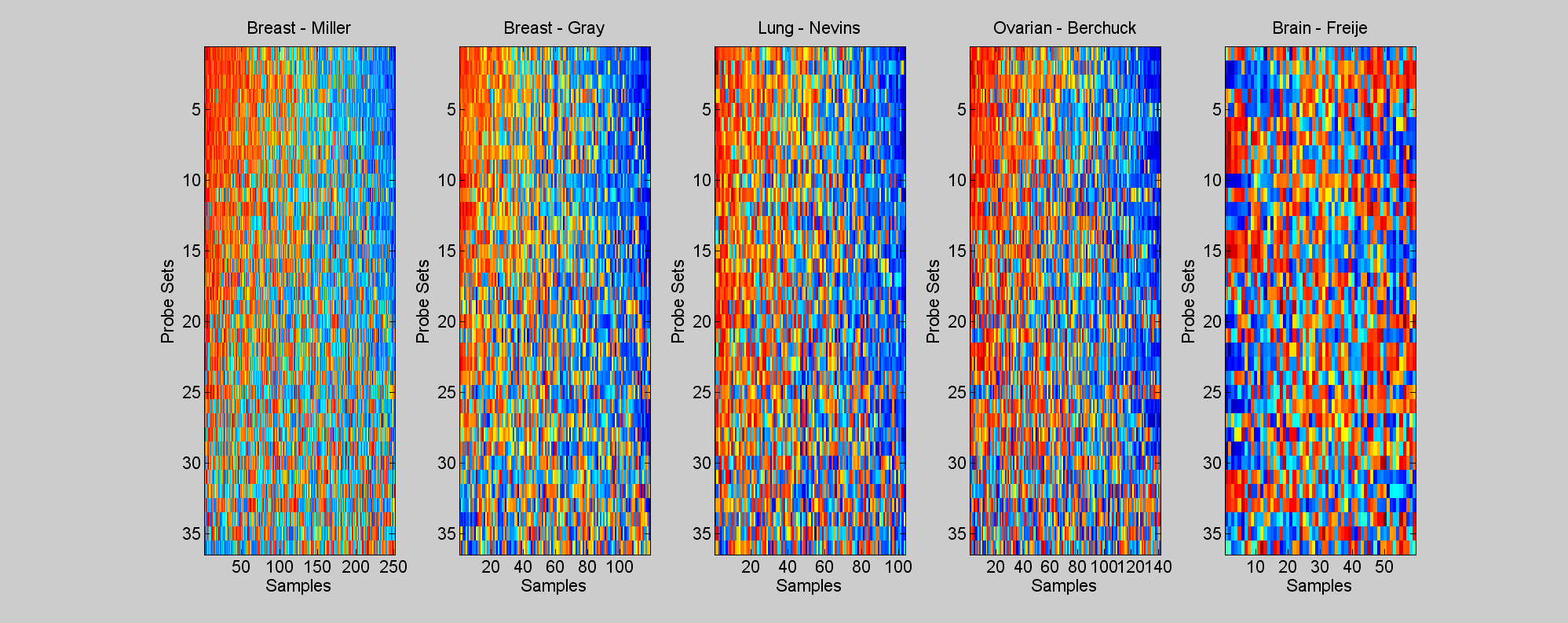

Supplement: Figure S1 — The coordinated expression of the latent factors in the five indicated cancer datasets of breast, lung, ovarian and brain cancers. (5.76 MB ZIP) [file pcbi.1000920.s001.zip › fac31.png]

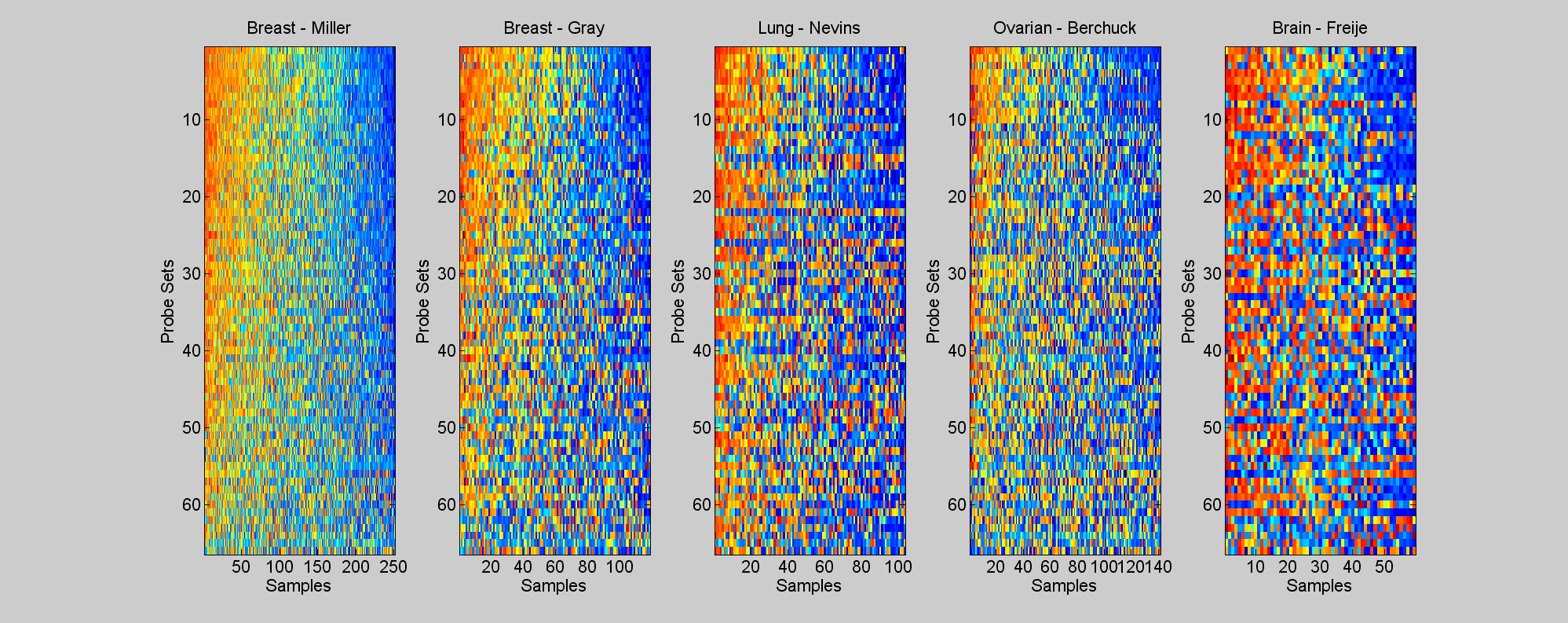

Supplement: Figure S1 — The coordinated expression of the latent factors in the five indicated cancer datasets of breast, lung, ovarian and brain cancers. (5.76 MB ZIP) [file pcbi.1000920.s001.zip › fac32.png]

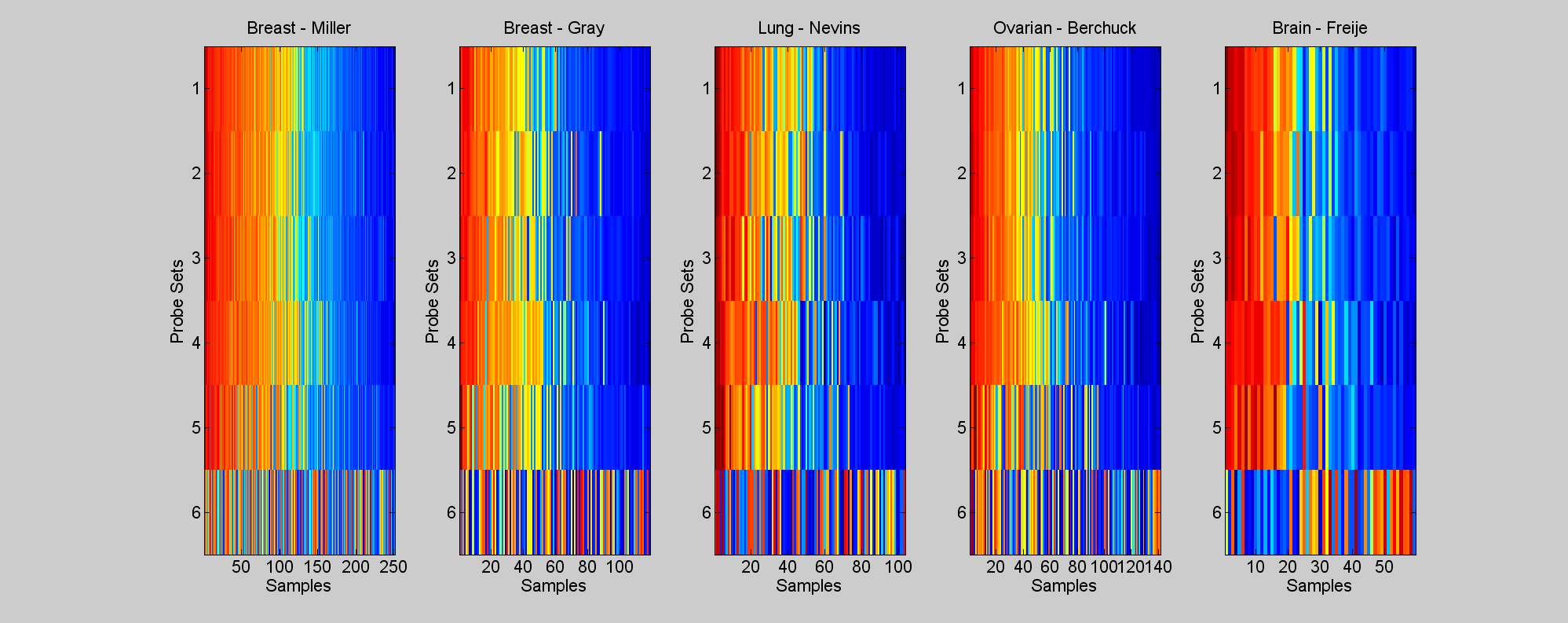

Supplement: Figure S1 — The coordinated expression of the latent factors in the five indicated cancer datasets of breast, lung, ovarian and brain cancers. (5.76 MB ZIP) [file pcbi.1000920.s001.zip › fac33.png]

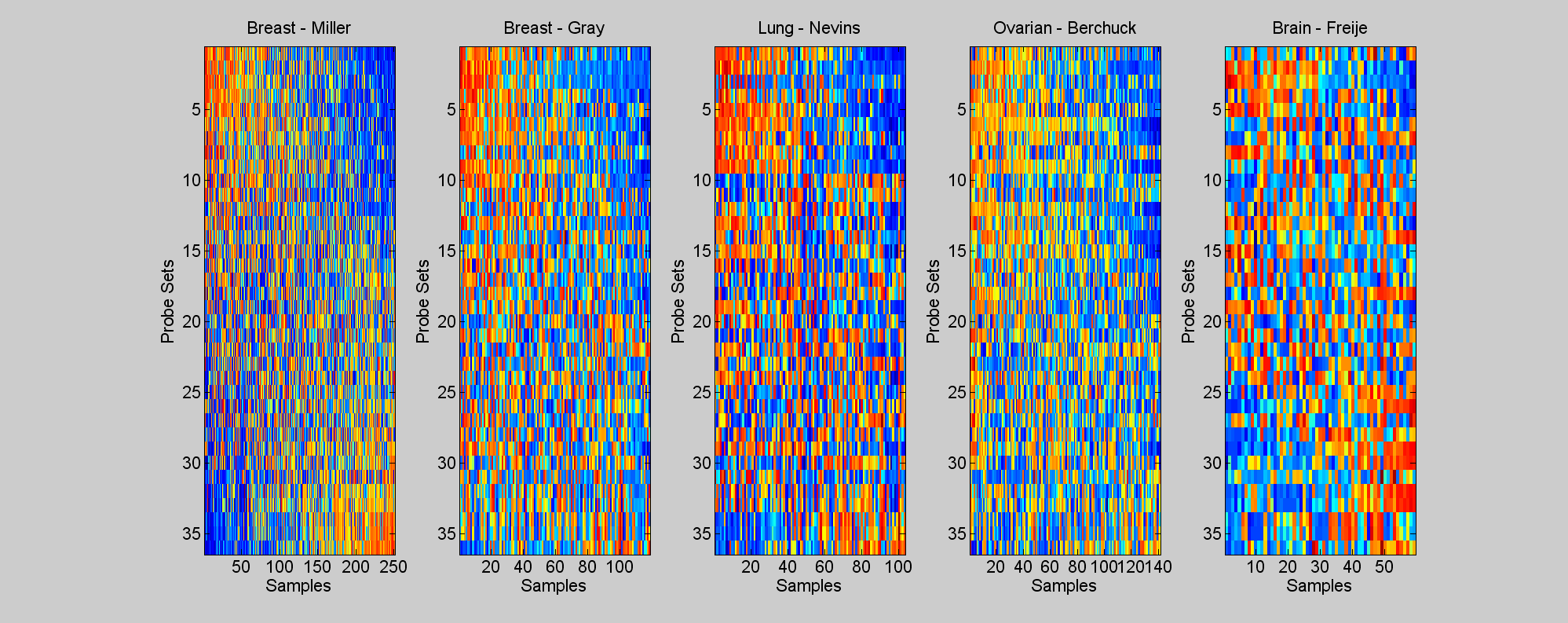

Supplement: Figure S1 — The coordinated expression of the latent factors in the five indicated cancer datasets of breast, lung, ovarian and brain cancers. (5.76 MB ZIP) [file pcbi.1000920.s001.zip › fac34.png]

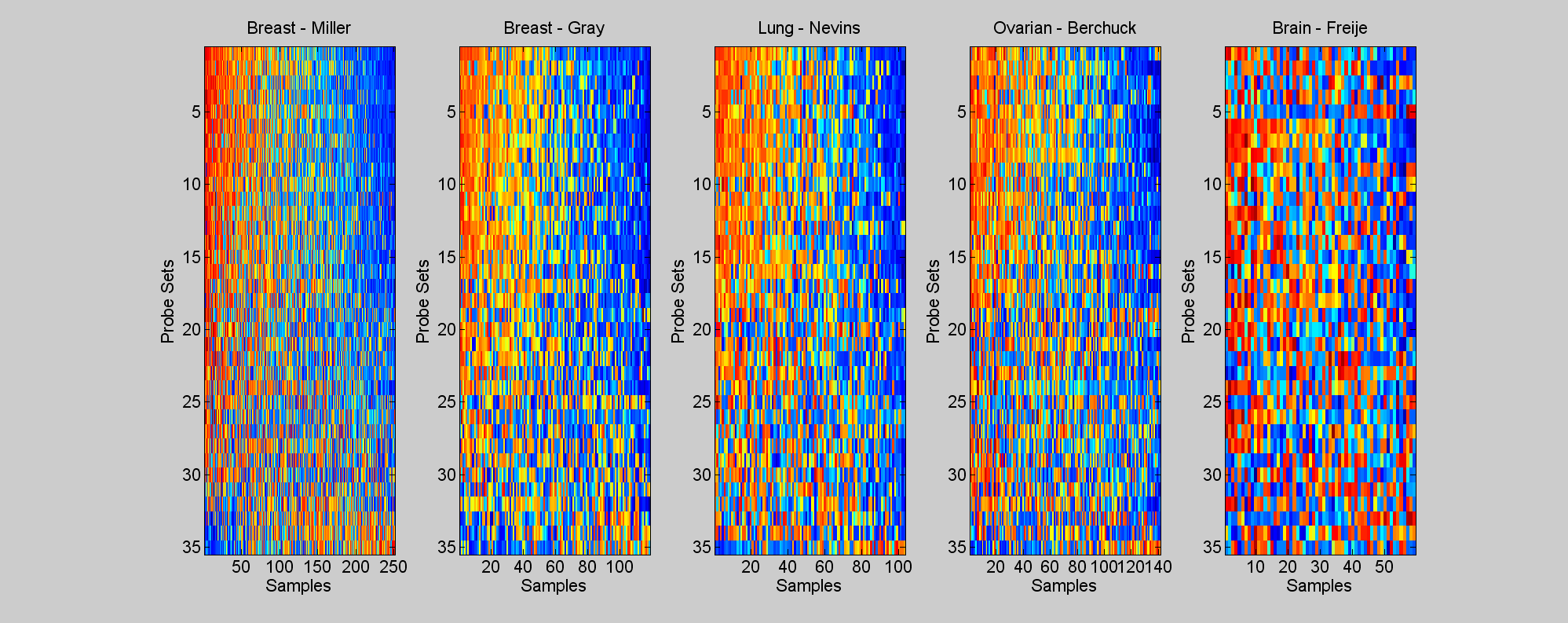

Supplement: Figure S1 — The coordinated expression of the latent factors in the five indicated cancer datasets of breast, lung, ovarian and brain cancers. (5.76 MB ZIP) [file pcbi.1000920.s001.zip › fac35.png]

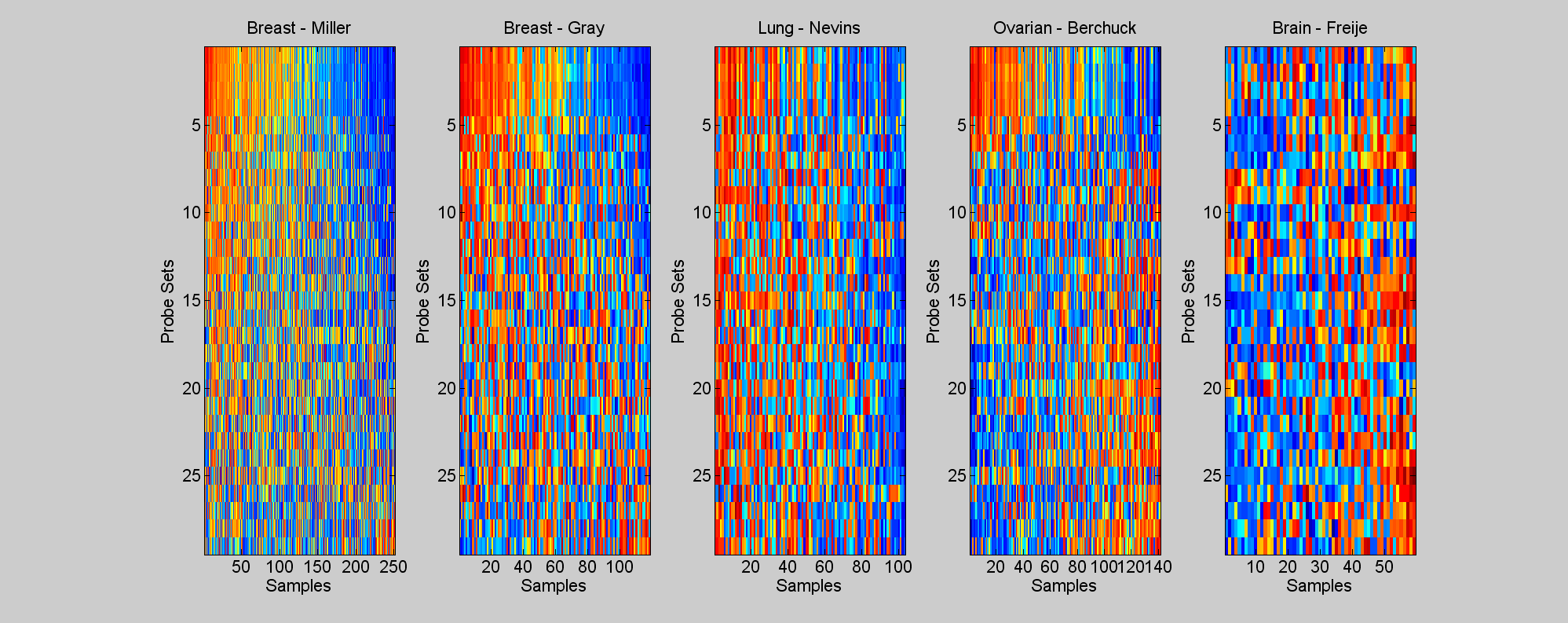

Supplement: Figure S1 — The coordinated expression of the latent factors in the five indicated cancer datasets of breast, lung, ovarian and brain cancers. (5.76 MB ZIP) [file pcbi.1000920.s001.zip › fac36.png]

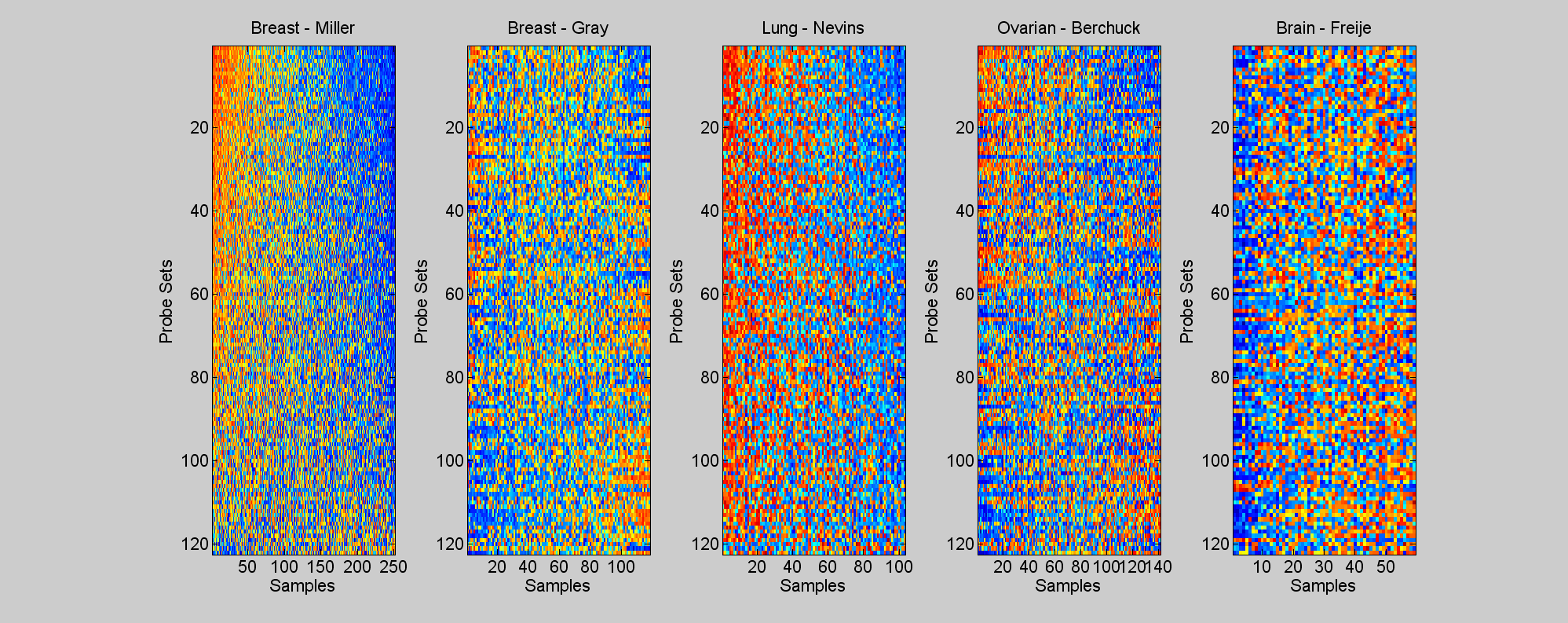

Supplement: Figure S1 — The coordinated expression of the latent factors in the five indicated cancer datasets of breast, lung, ovarian and brain cancers. (5.76 MB ZIP) [file pcbi.1000920.s001.zip › fac37.png]

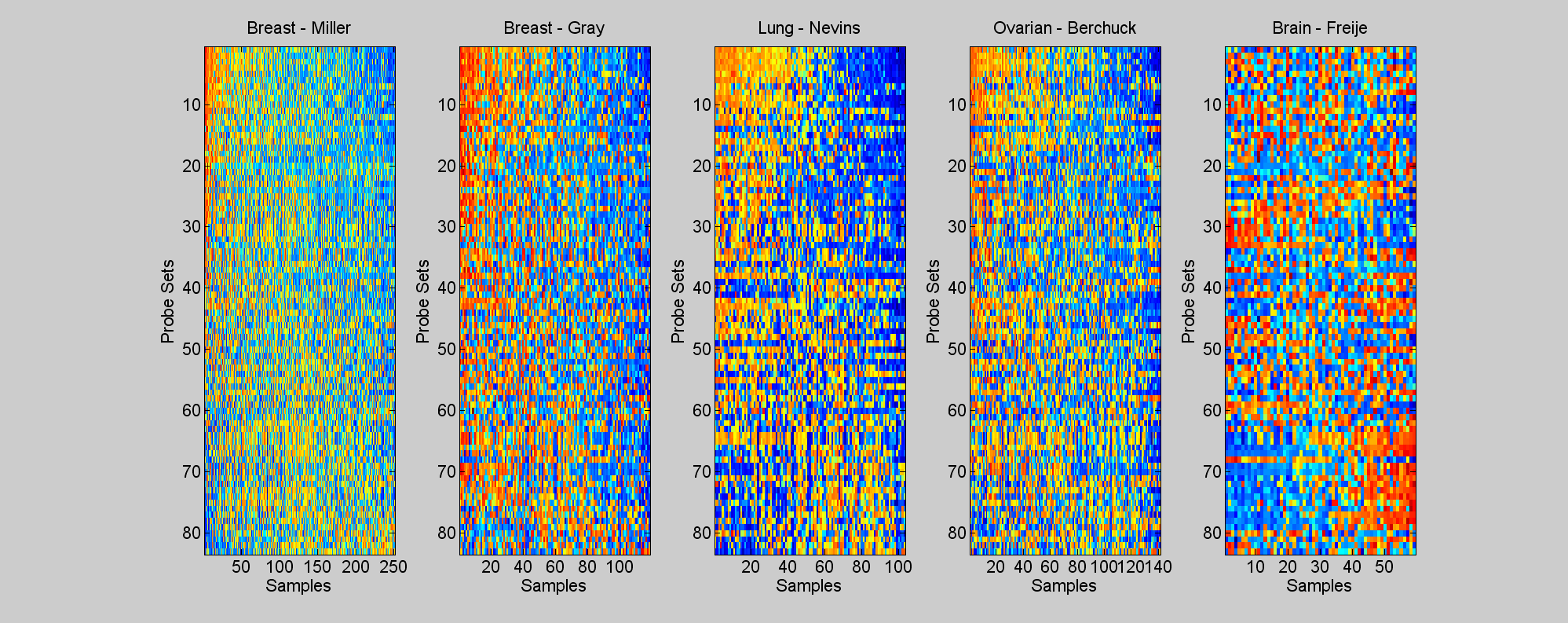

Supplement: Figure S1 — The coordinated expression of the latent factors in the five indicated cancer datasets of breast, lung, ovarian and brain cancers. (5.76 MB ZIP) [file pcbi.1000920.s001.zip › fac38.png]

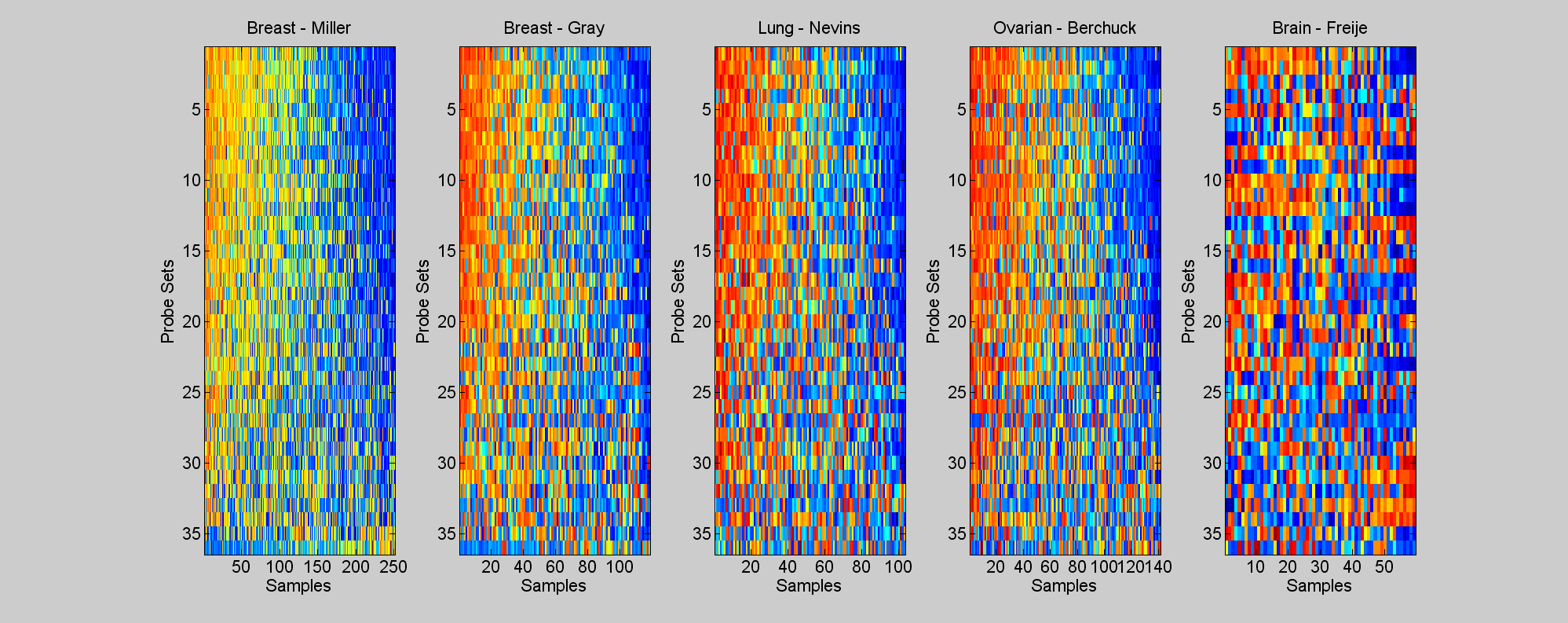

Supplement: Figure S1 — The coordinated expression of the latent factors in the five indicated cancer datasets of breast, lung, ovarian and brain cancers. (5.76 MB ZIP) [file pcbi.1000920.s001.zip › fac39.png]

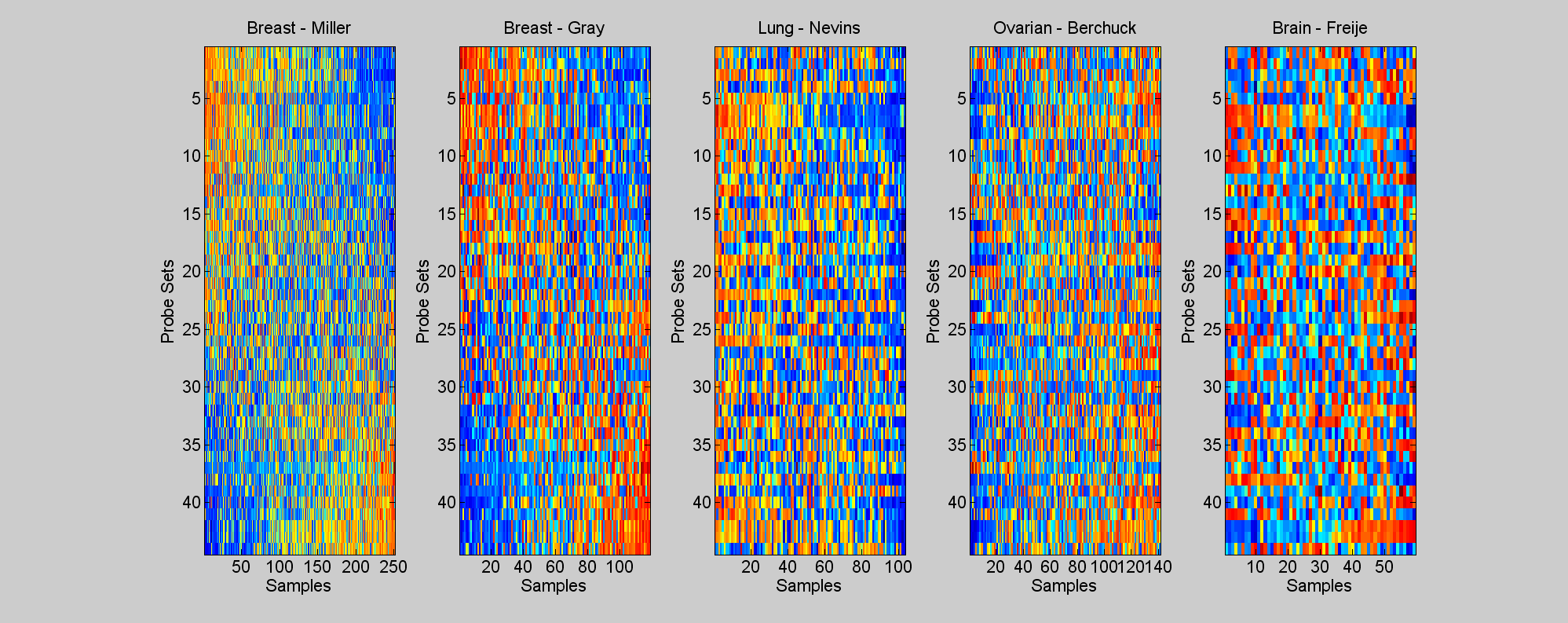

Supplement: Figure S1 — The coordinated expression of the latent factors in the five indicated cancer datasets of breast, lung, ovarian and brain cancers. (5.76 MB ZIP) [file pcbi.1000920.s001.zip › fac40.png]

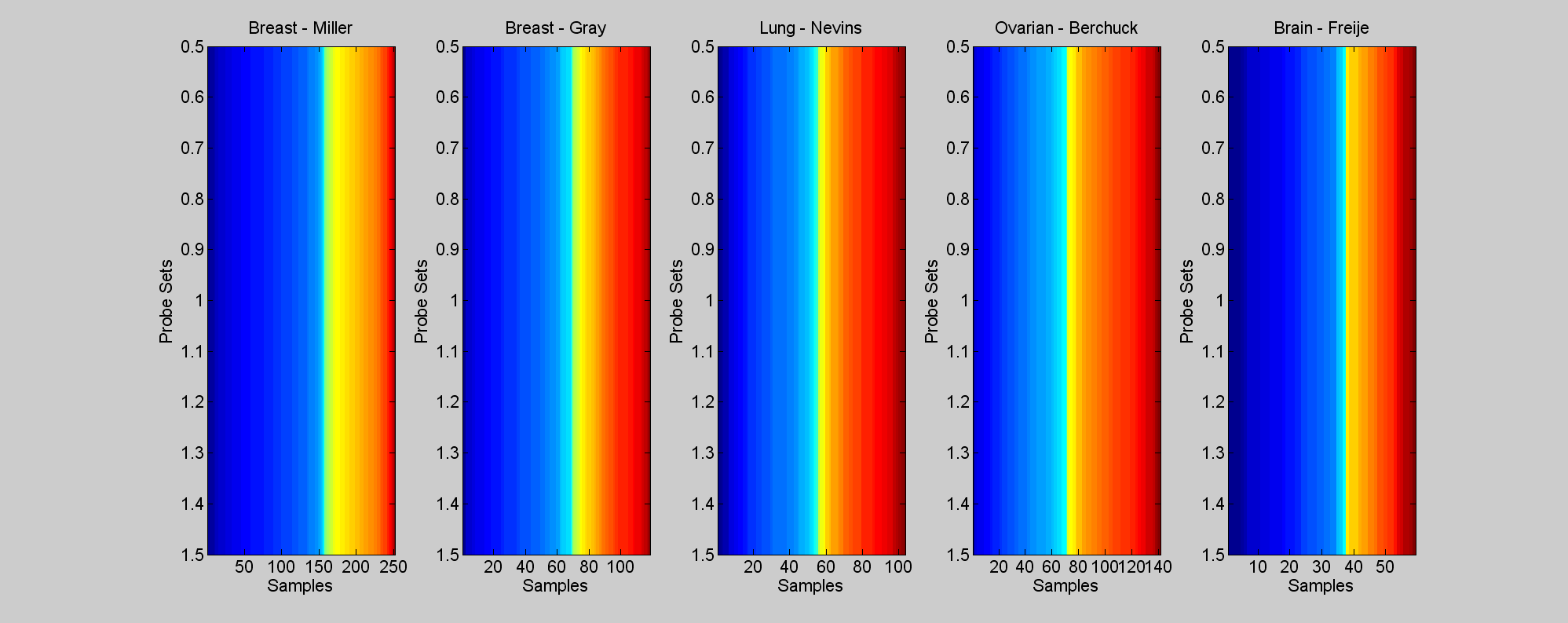

Supplement: Figure S1 — The coordinated expression of the latent factors in the five indicated cancer datasets of breast, lung, ovarian and brain cancers. (5.76 MB ZIP) [file pcbi.1000920.s001.zip › fac41.png]

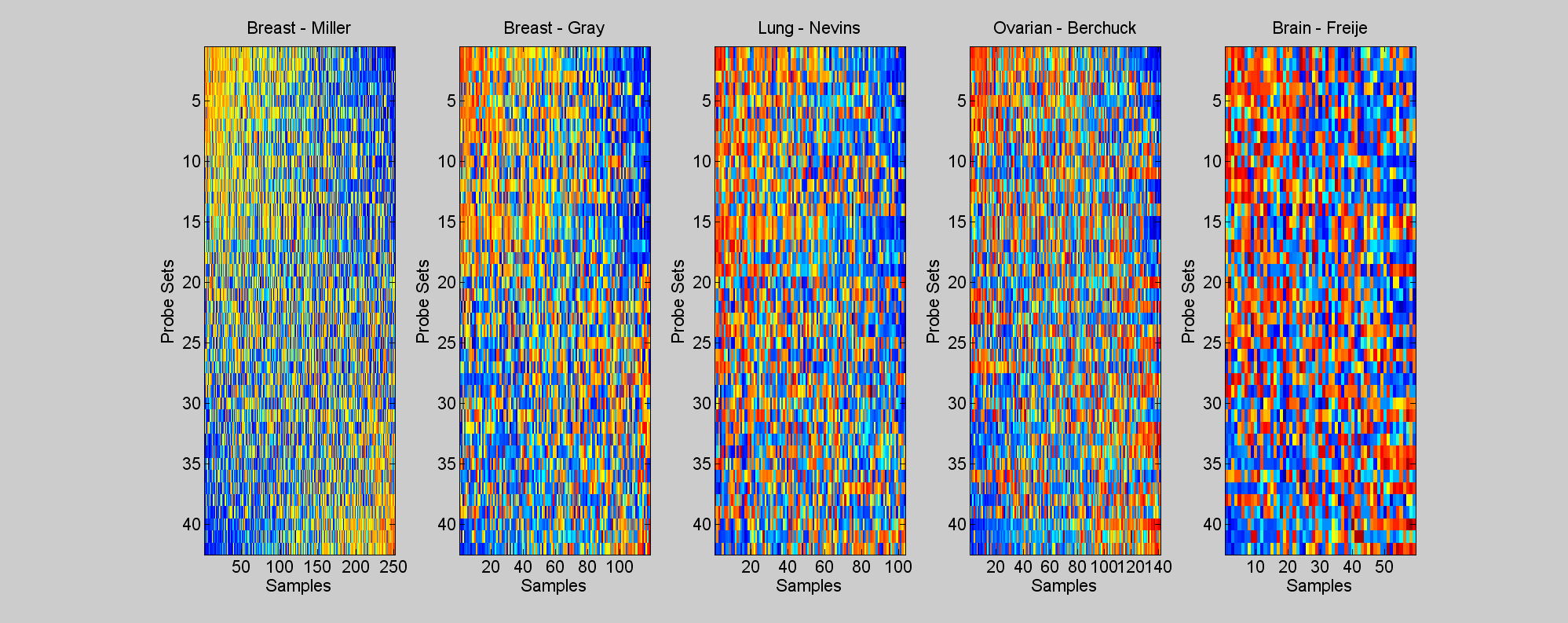

Supplement: Figure S1 — The coordinated expression of the latent factors in the five indicated cancer datasets of breast, lung, ovarian and brain cancers. (5.76 MB ZIP) [file pcbi.1000920.s001.zip › fac42.png]

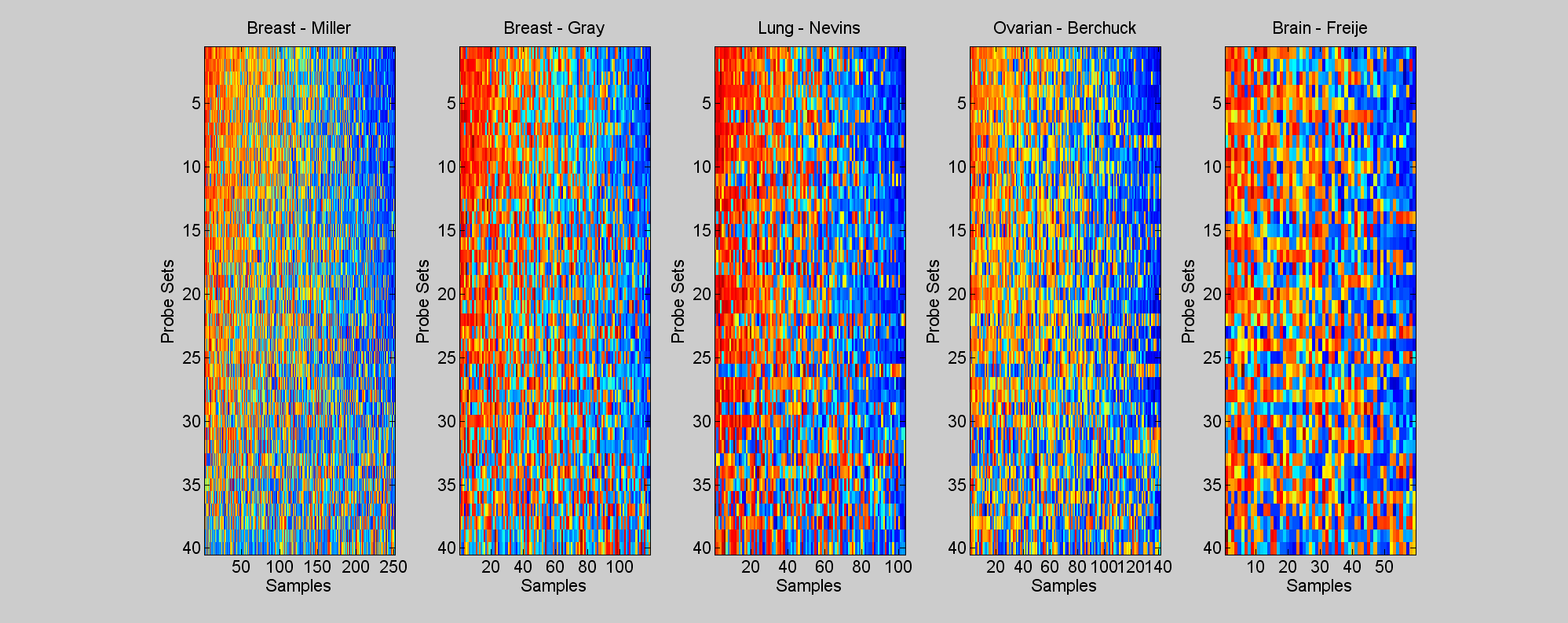

Supplement: Figure S1 — The coordinated expression of the latent factors in the five indicated cancer datasets of breast, lung, ovarian and brain cancers. (5.76 MB ZIP) [file pcbi.1000920.s001.zip › fac43.png]

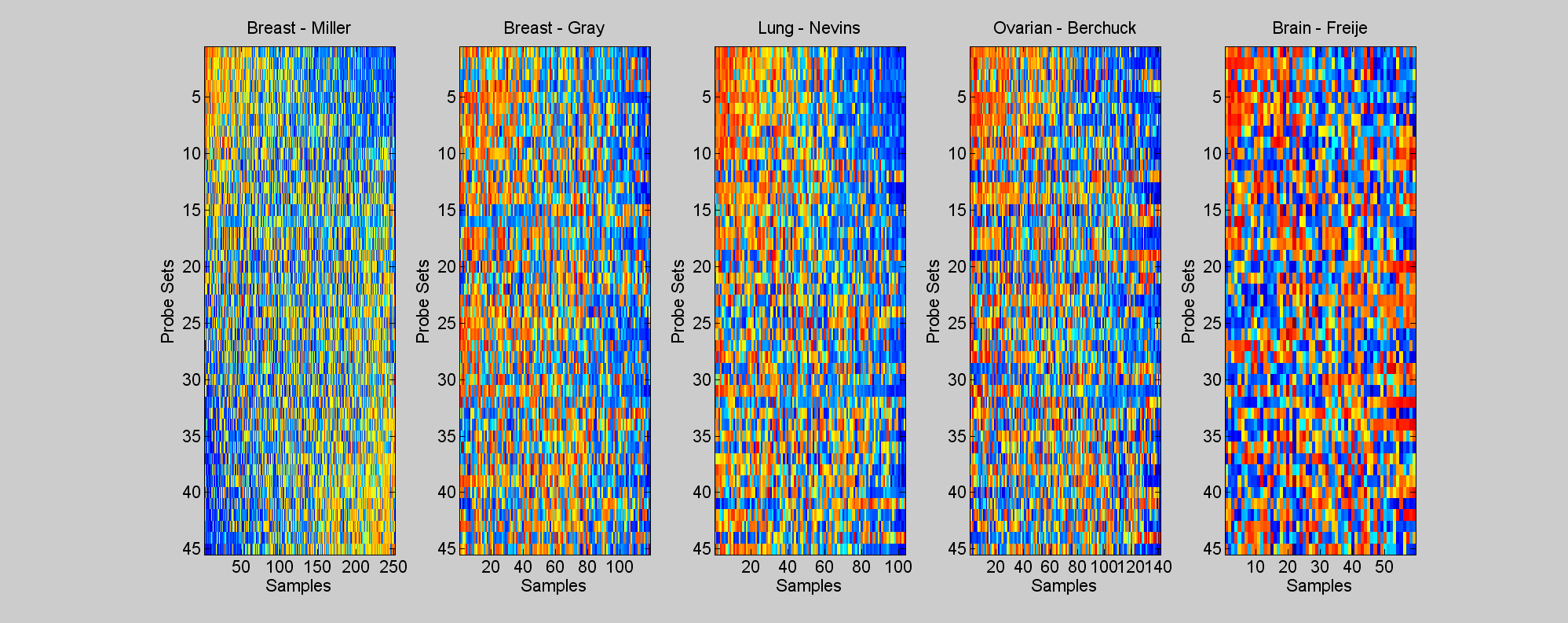

Supplement: Figure S1 — The coordinated expression of the latent factors in the five indicated cancer datasets of breast, lung, ovarian and brain cancers. (5.76 MB ZIP) [file pcbi.1000920.s001.zip › fac44.png]

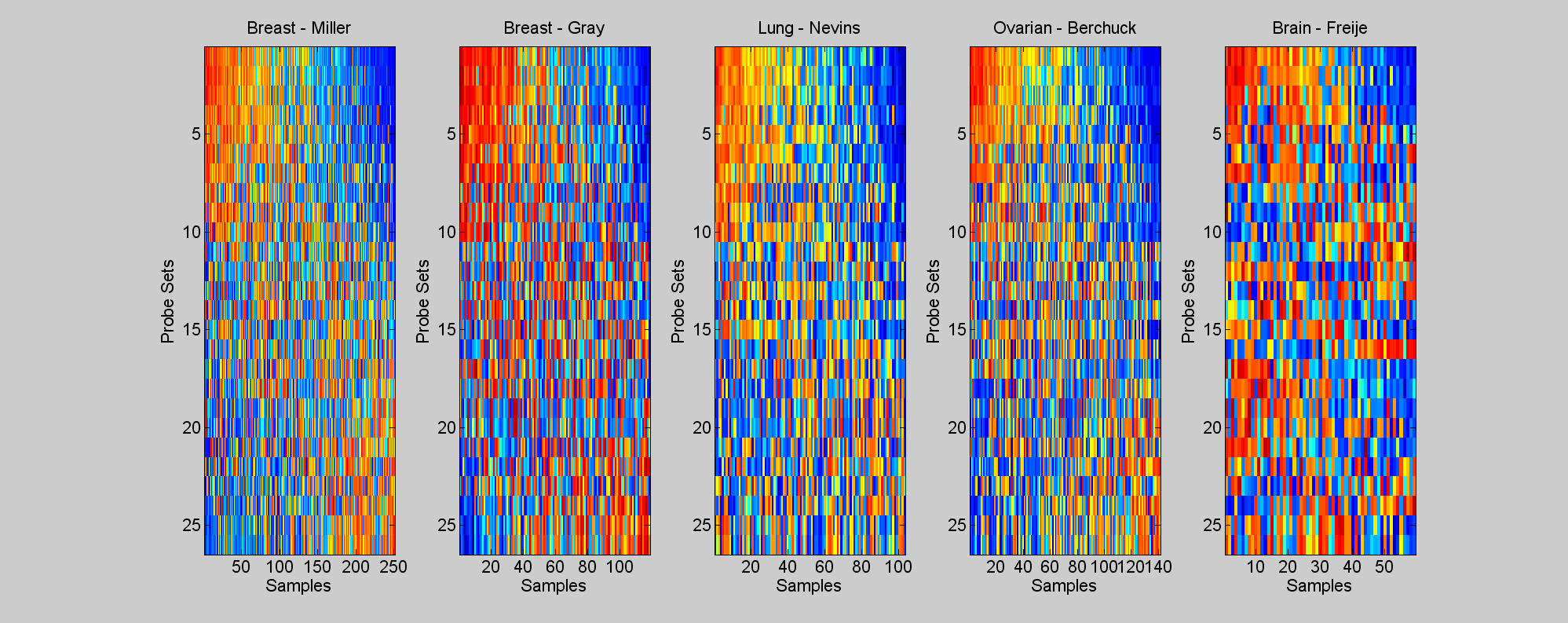

Supplement: Figure S1 — The coordinated expression of the latent factors in the five indicated cancer datasets of breast, lung, ovarian and brain cancers. (5.76 MB ZIP) [file pcbi.1000920.s001.zip › fac45.png]

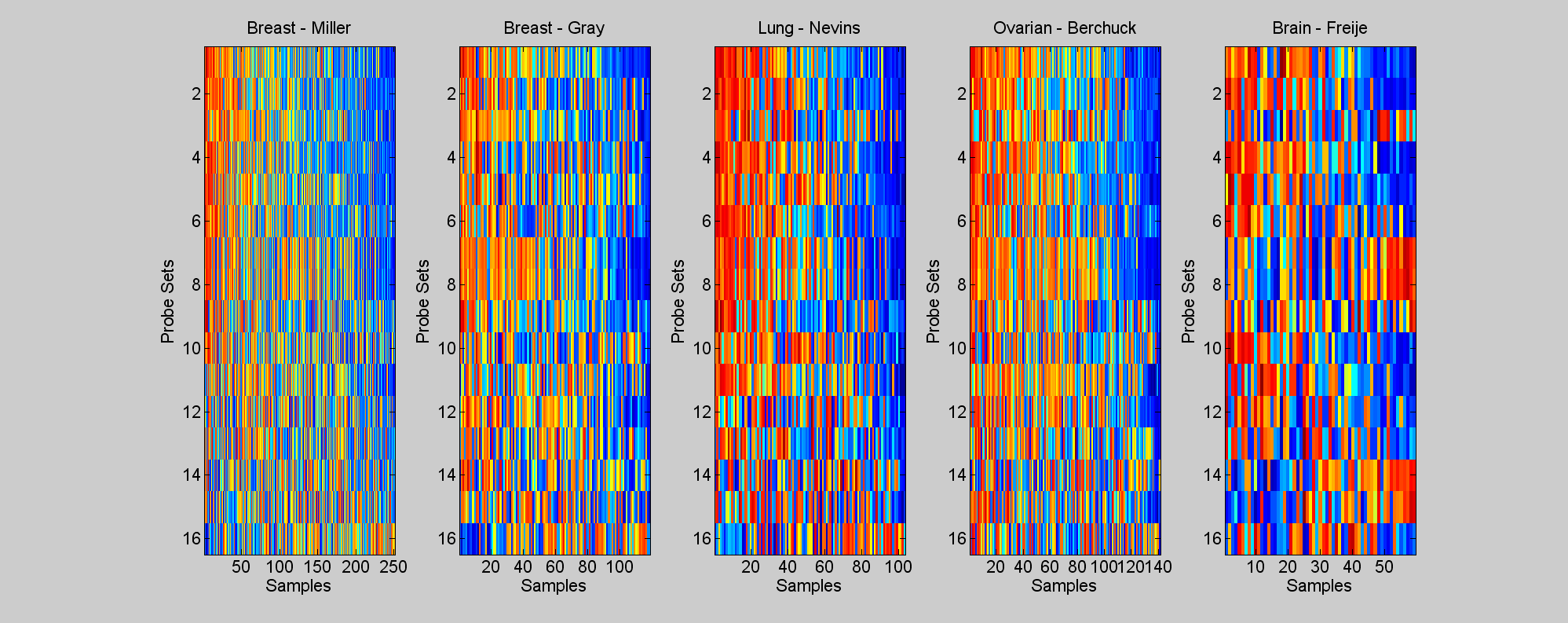

Supplement: Figure S1 — The coordinated expression of the latent factors in the five indicated cancer datasets of breast, lung, ovarian and brain cancers. (5.76 MB ZIP) [file pcbi.1000920.s001.zip › fac46.png]

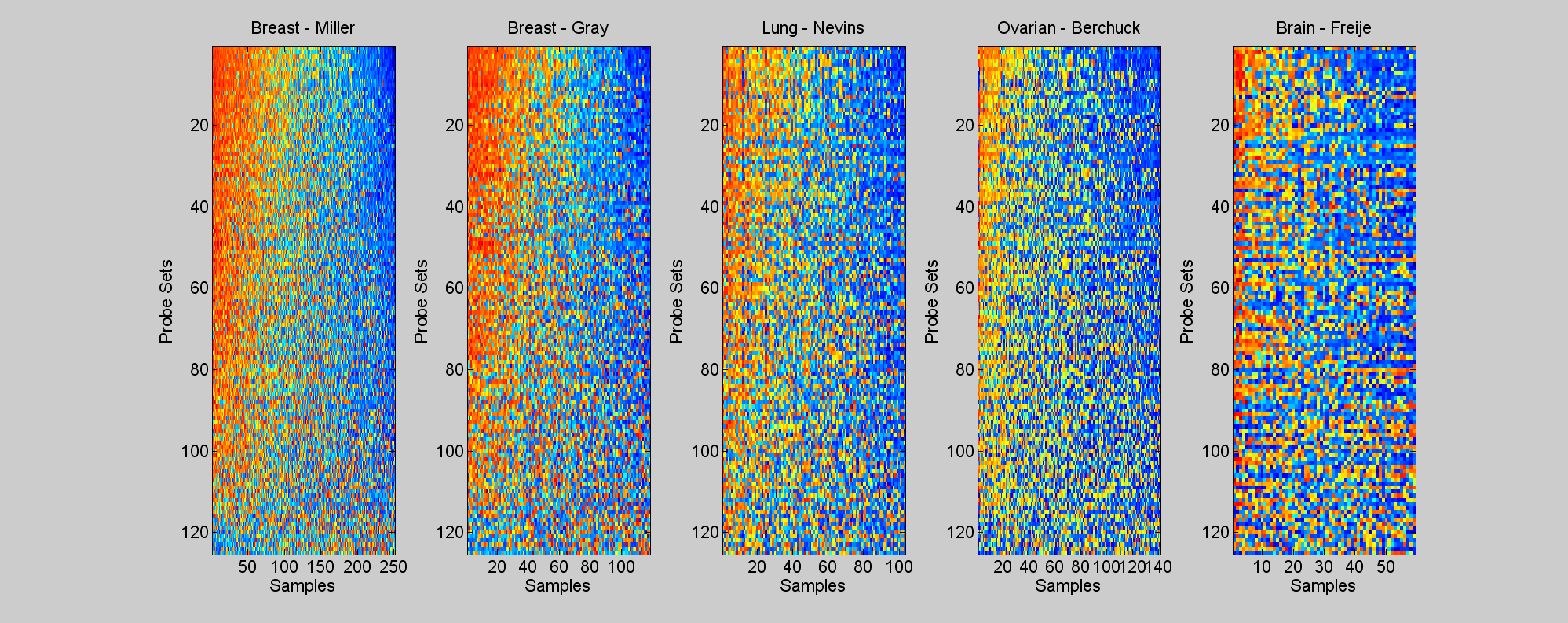

Supplement: Figure S1 — The coordinated expression of the latent factors in the five indicated cancer datasets of breast, lung, ovarian and brain cancers. (5.76 MB ZIP) [file pcbi.1000920.s001.zip › fac47.png]

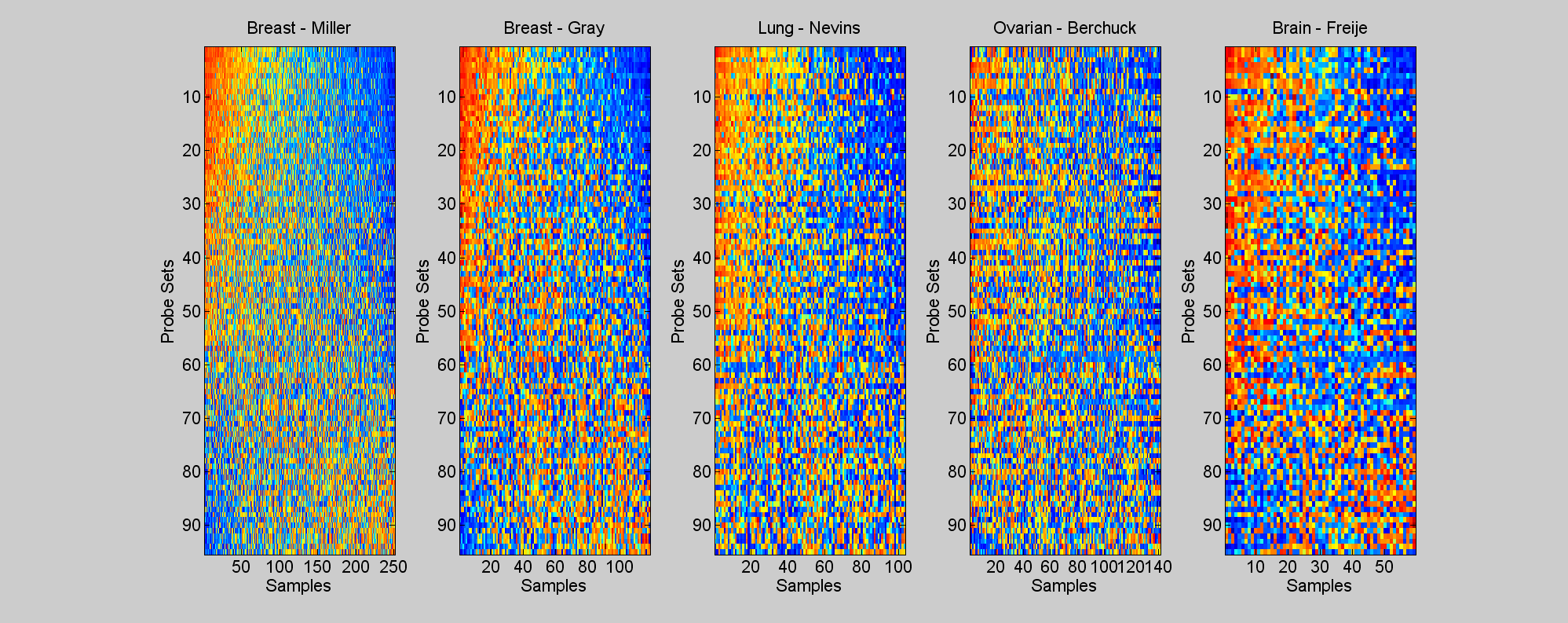

Supplement: Figure S1 — The coordinated expression of the latent factors in the five indicated cancer datasets of breast, lung, ovarian and brain cancers. (5.76 MB ZIP) [file pcbi.1000920.s001.zip › fac48.png]

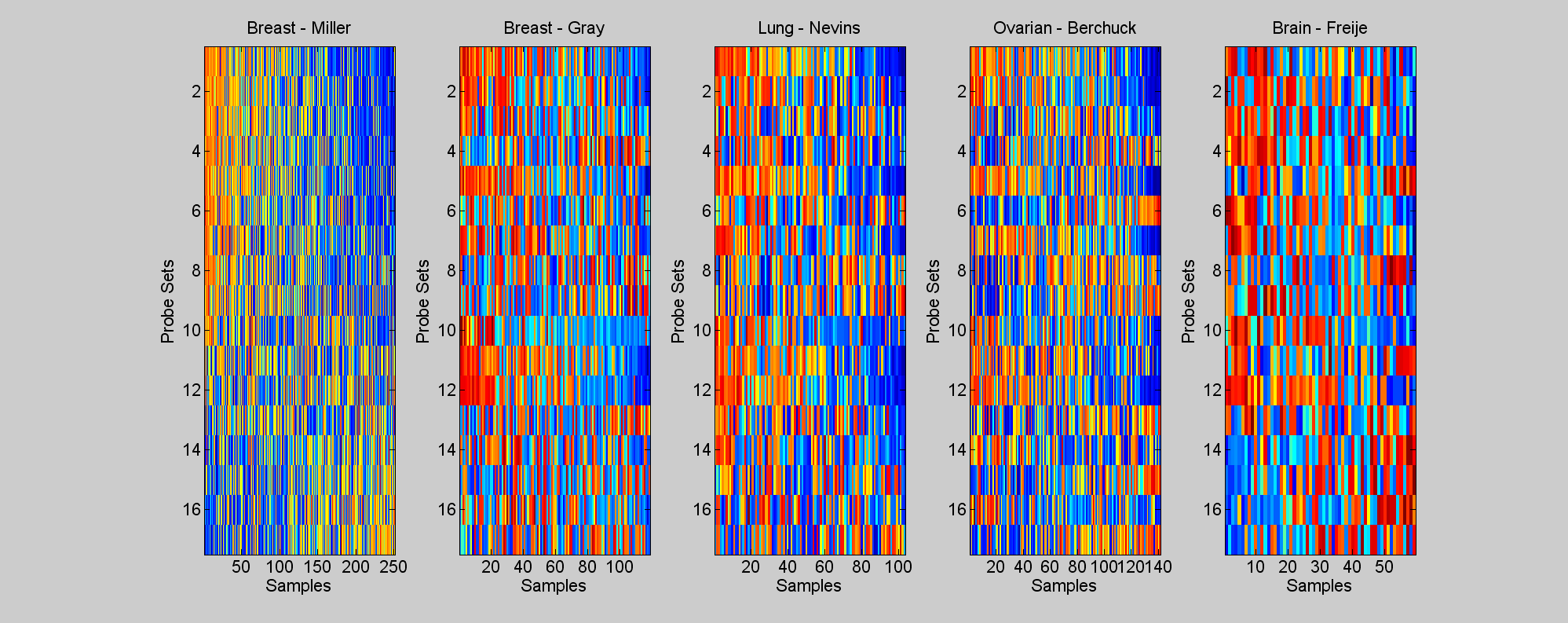

Supplement: Figure S1 — The coordinated expression of the latent factors in the five indicated cancer datasets of breast, lung, ovarian and brain cancers. (5.76 MB ZIP) [file pcbi.1000920.s001.zip › fac49.png]

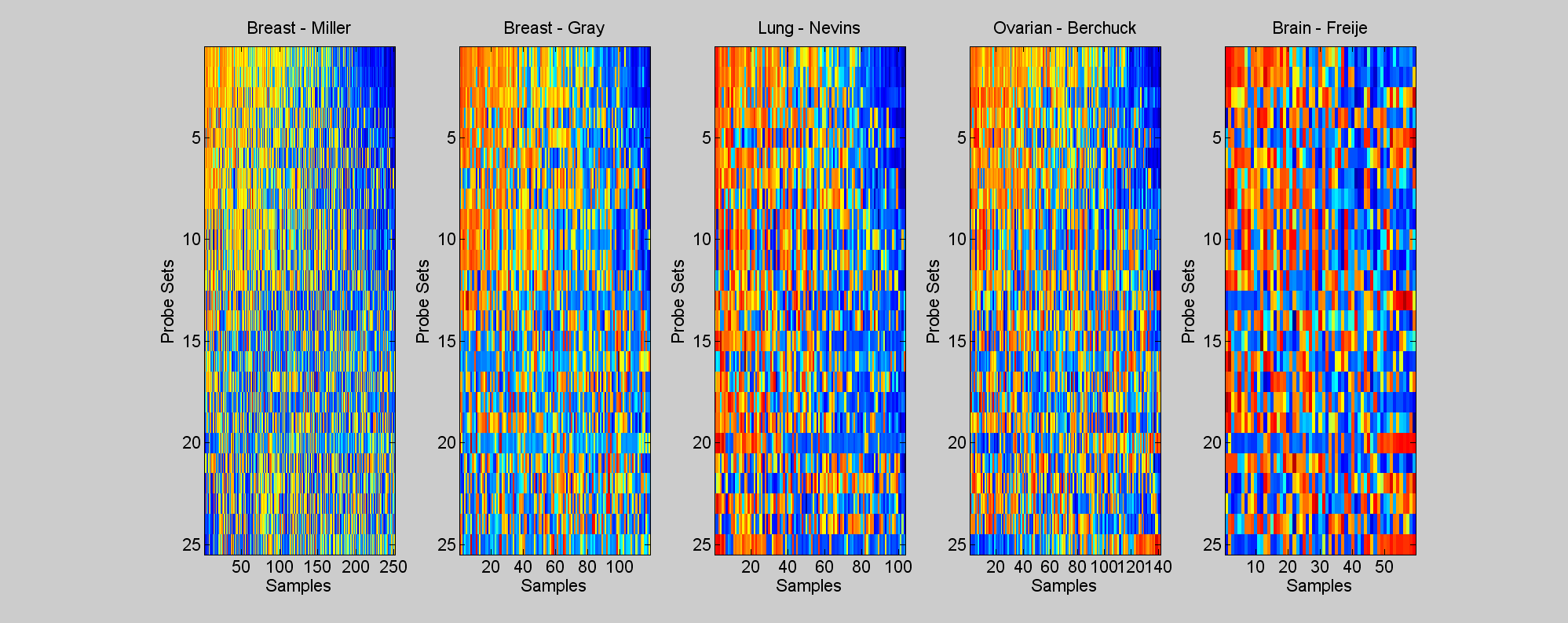

Supplement: Figure S1 — The coordinated expression of the latent factors in the five indicated cancer datasets of breast, lung, ovarian and brain cancers. (5.76 MB ZIP) [file pcbi.1000920.s001.zip › fac51.png]

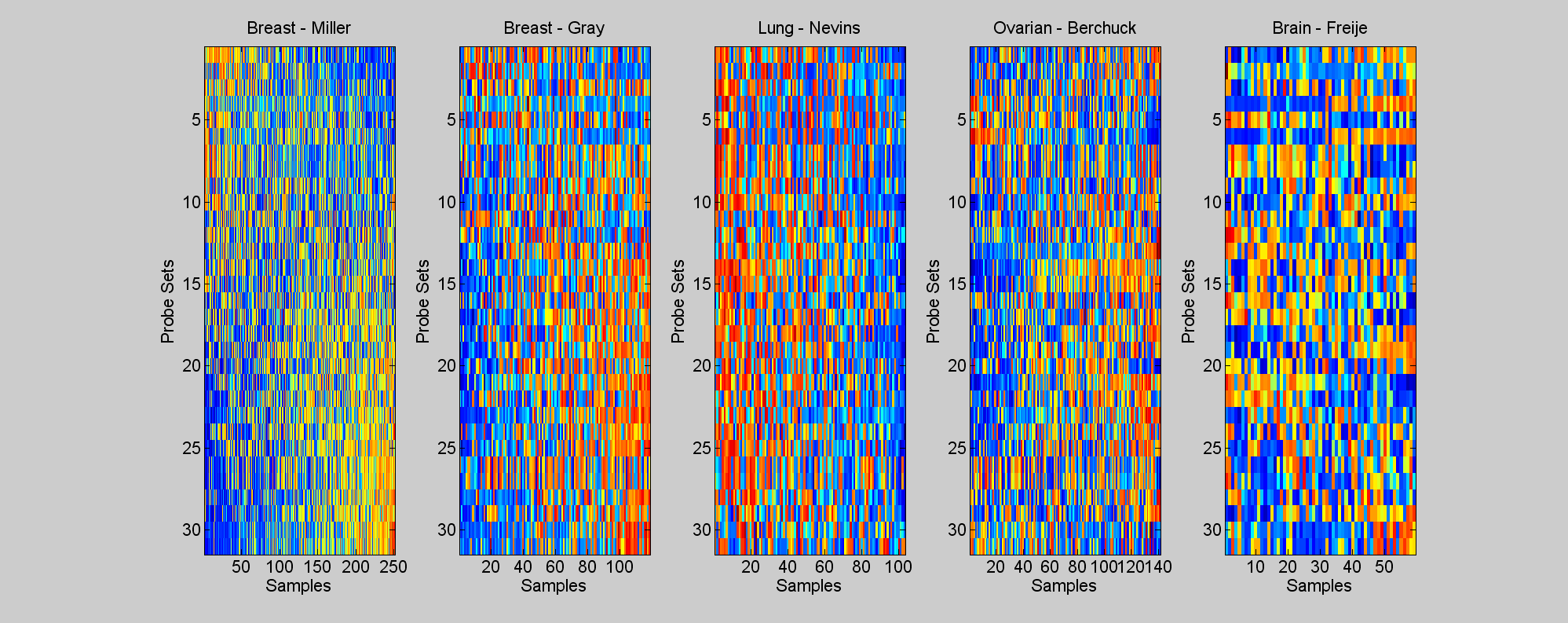

Supplement: Figure S1 — The coordinated expression of the latent factors in the five indicated cancer datasets of breast, lung, ovarian and brain cancers. (5.76 MB ZIP) [file pcbi.1000920.s001.zip › fac52.png]

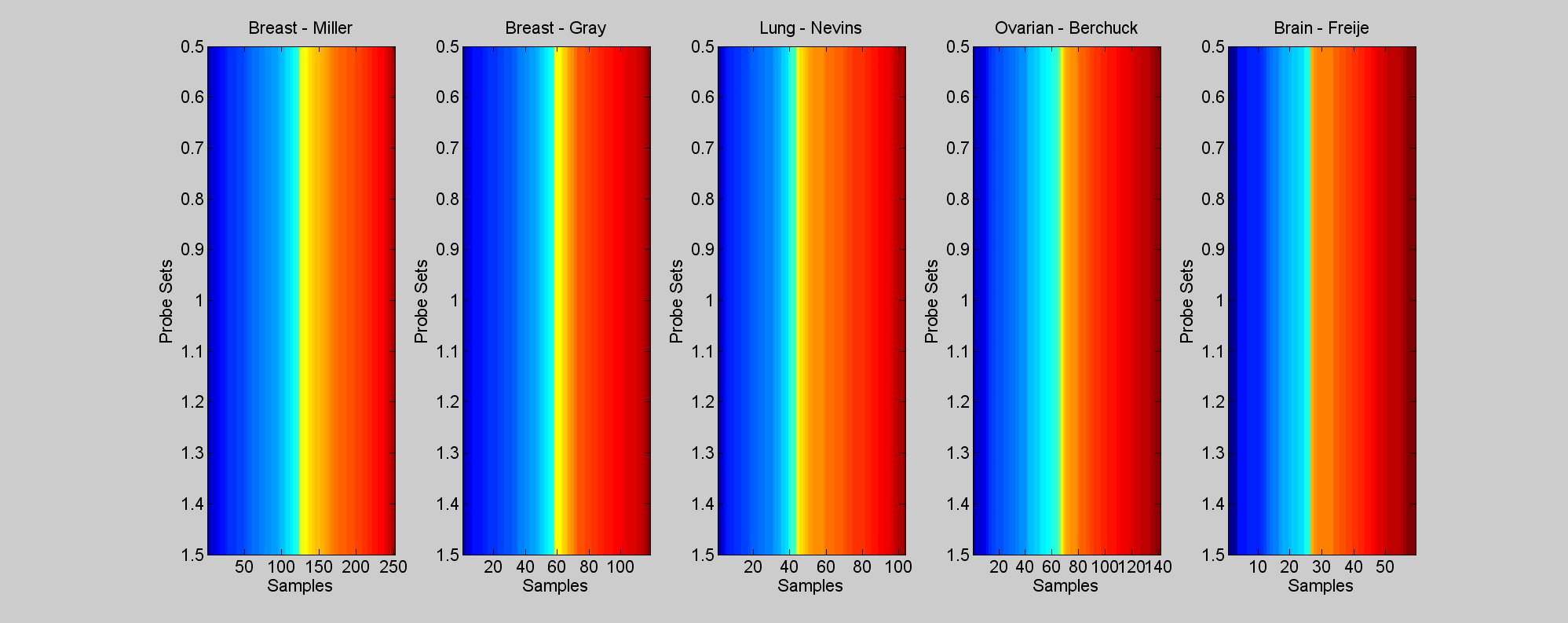

Supplement: Figure S1 — The coordinated expression of the latent factors in the five indicated cancer datasets of breast, lung, ovarian and brain cancers. (5.76 MB ZIP) [file pcbi.1000920.s001.zip › fac53.png]

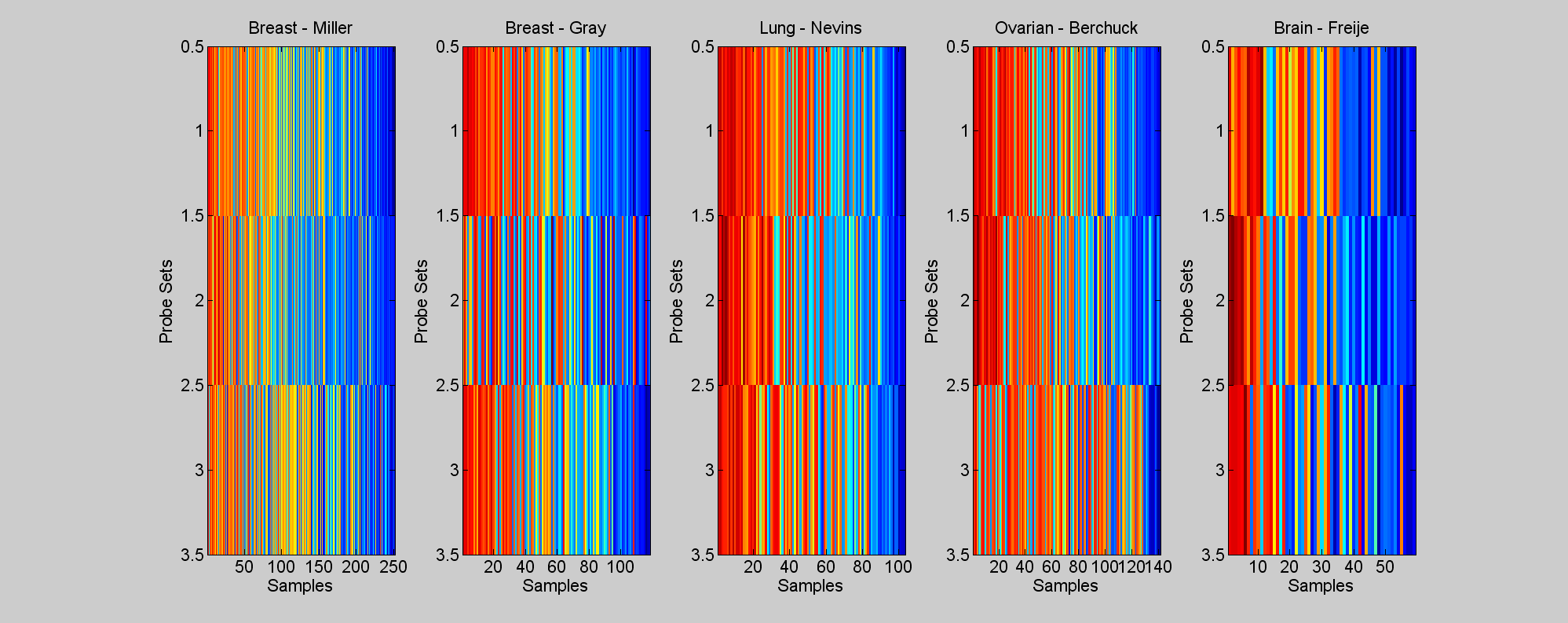

Supplement: Figure S1 — The coordinated expression of the latent factors in the five indicated cancer datasets of breast, lung, ovarian and brain cancers. (5.76 MB ZIP) [file pcbi.1000920.s001.zip › fac54.png]

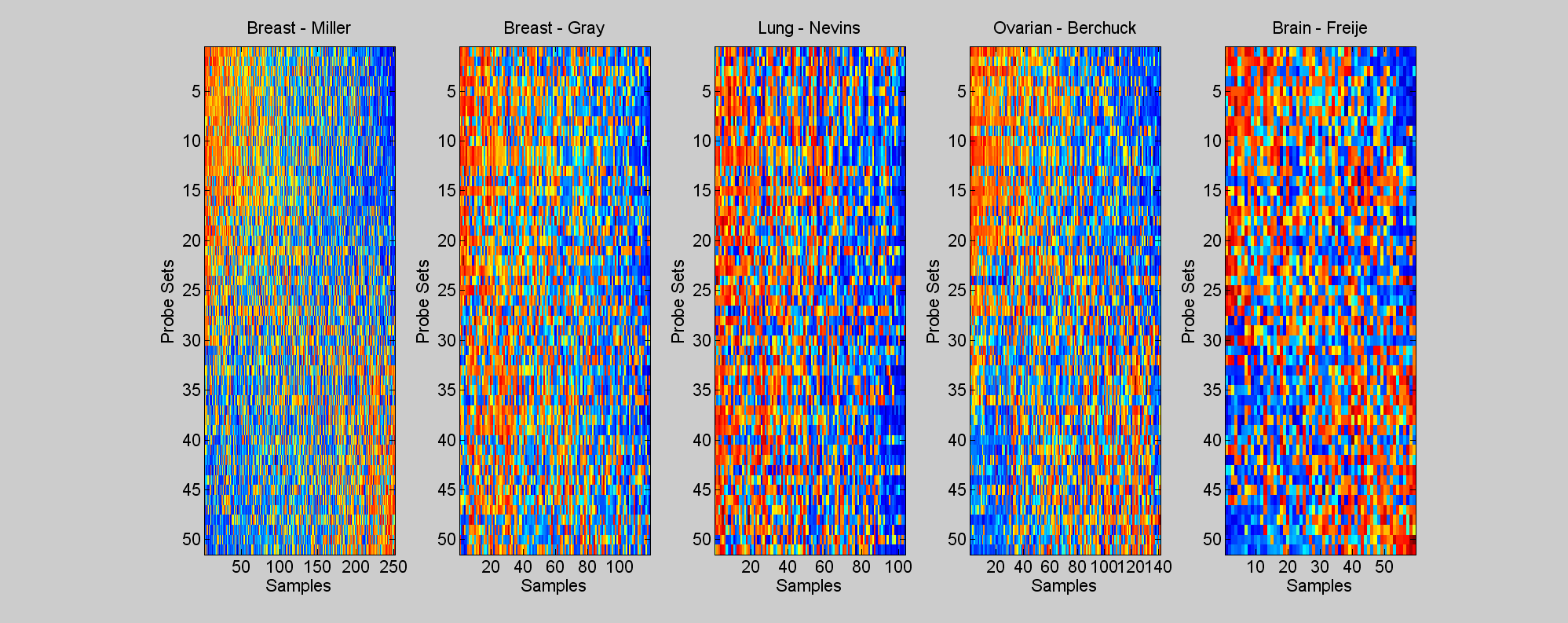

Supplement: Figure S1 — The coordinated expression of the latent factors in the five indicated cancer datasets of breast, lung, ovarian and brain cancers. (5.76 MB ZIP) [file pcbi.1000920.s001.zip › fac55.png]

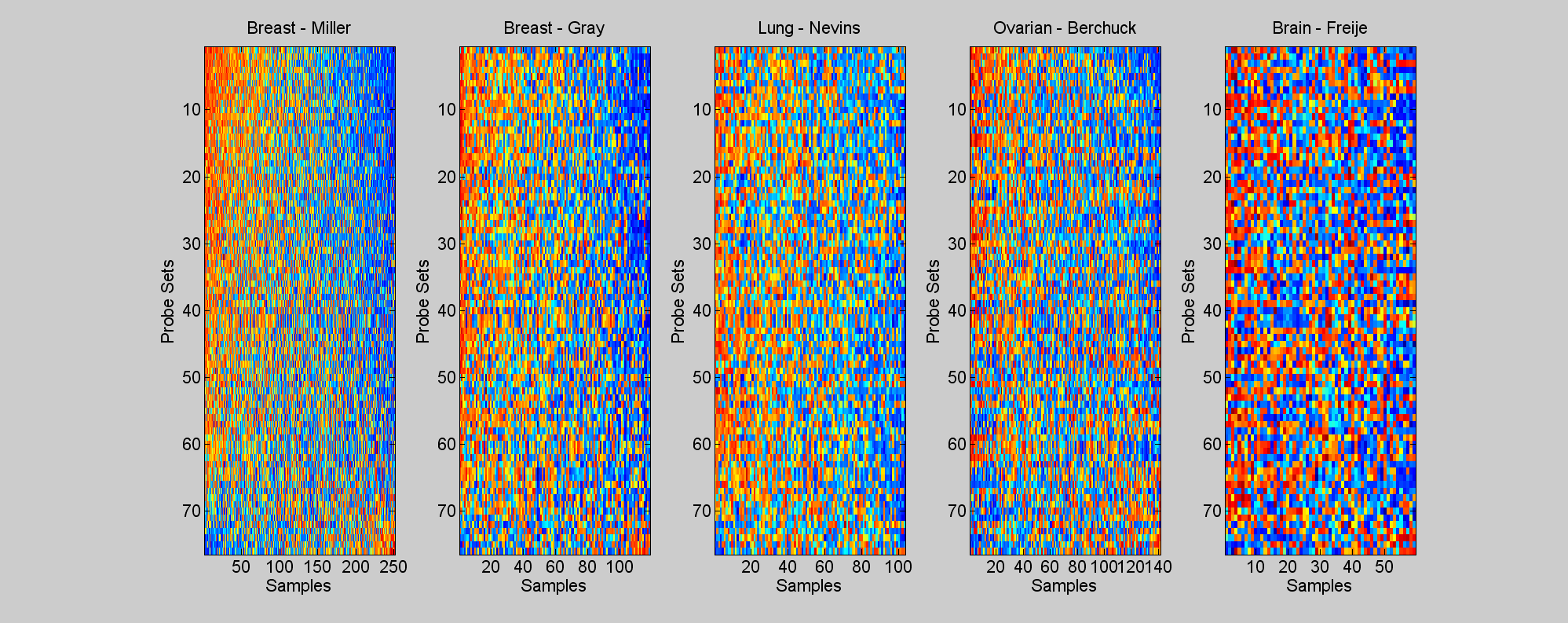

Supplement: Figure S1 — The coordinated expression of the latent factors in the five indicated cancer datasets of breast, lung, ovarian and brain cancers. (5.76 MB ZIP) [file pcbi.1000920.s001.zip › fac56.png]

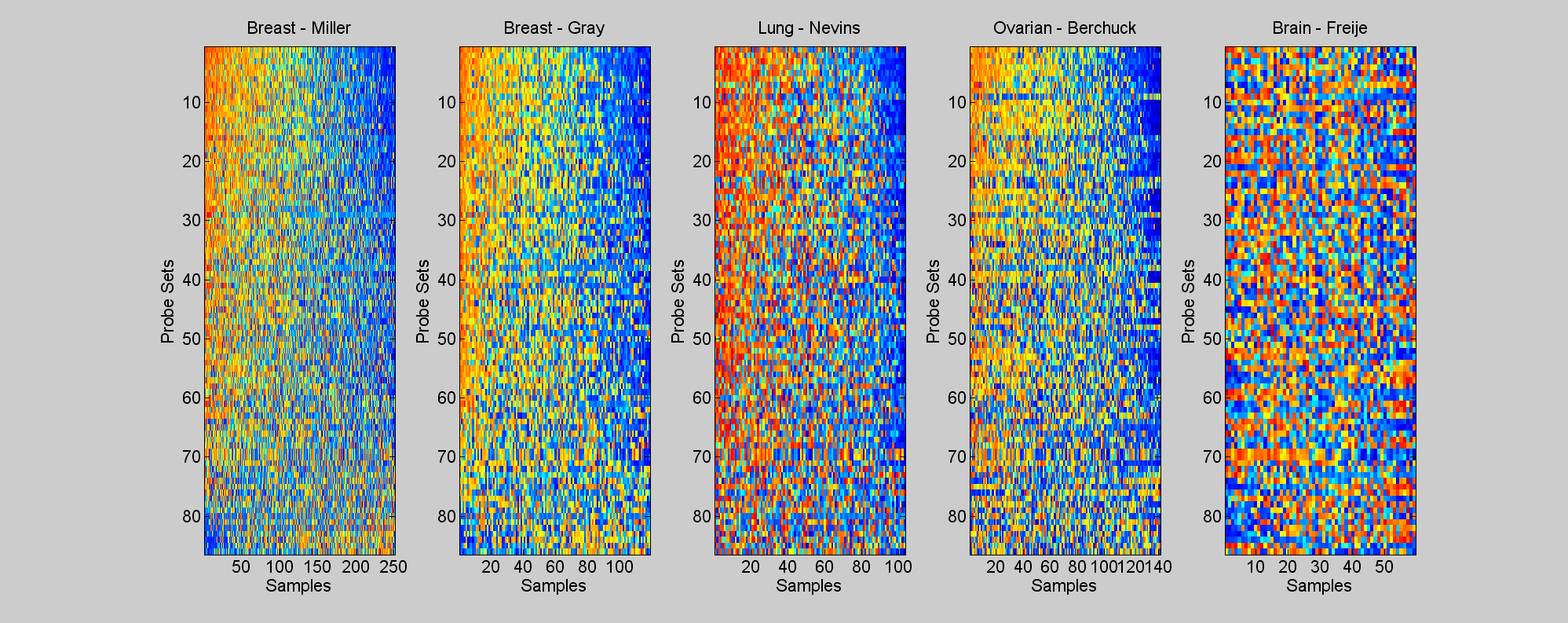

Supplement: Figure S1 — The coordinated expression of the latent factors in the five indicated cancer datasets of breast, lung, ovarian and brain cancers. (5.76 MB ZIP) [file pcbi.1000920.s001.zip › fac1.png]

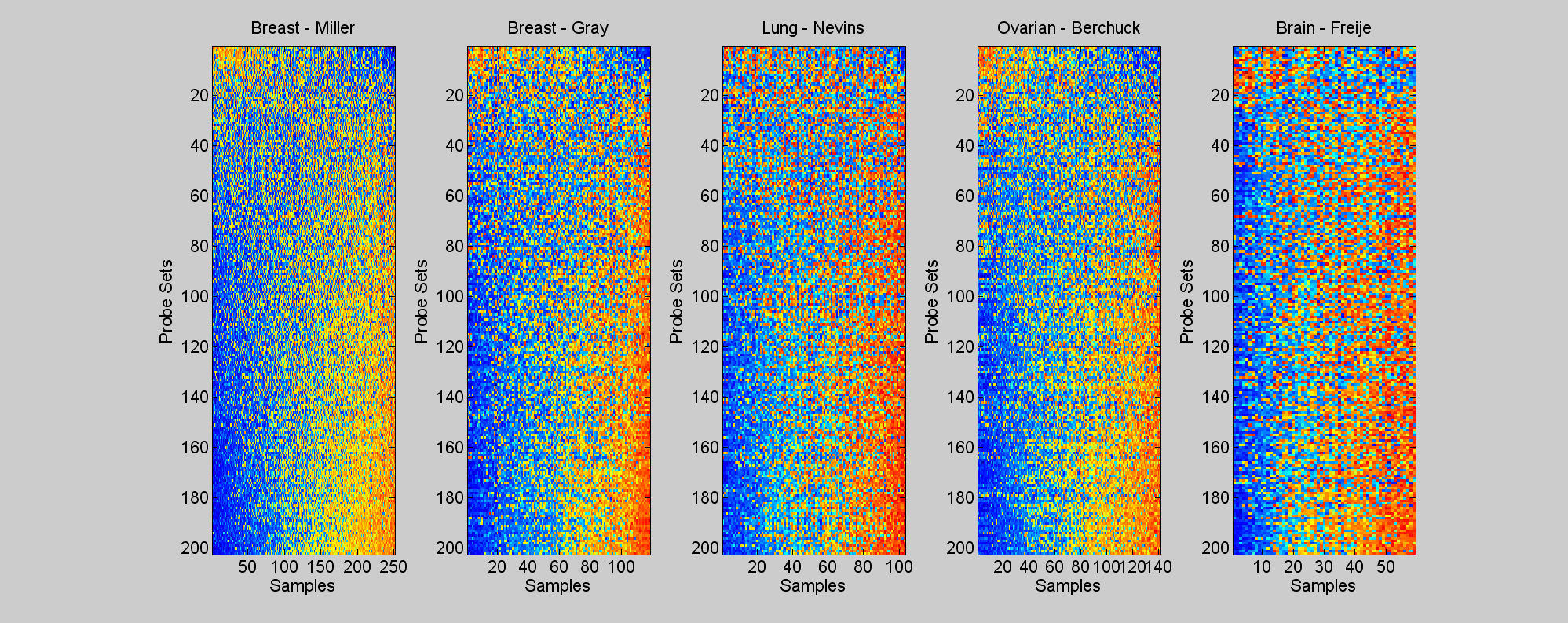

Supplement: Figure S1 — The coordinated expression of the latent factors in the five indicated cancer datasets of breast, lung, ovarian and brain cancers. (5.76 MB ZIP) [file pcbi.1000920.s001.zip › fac2.png]

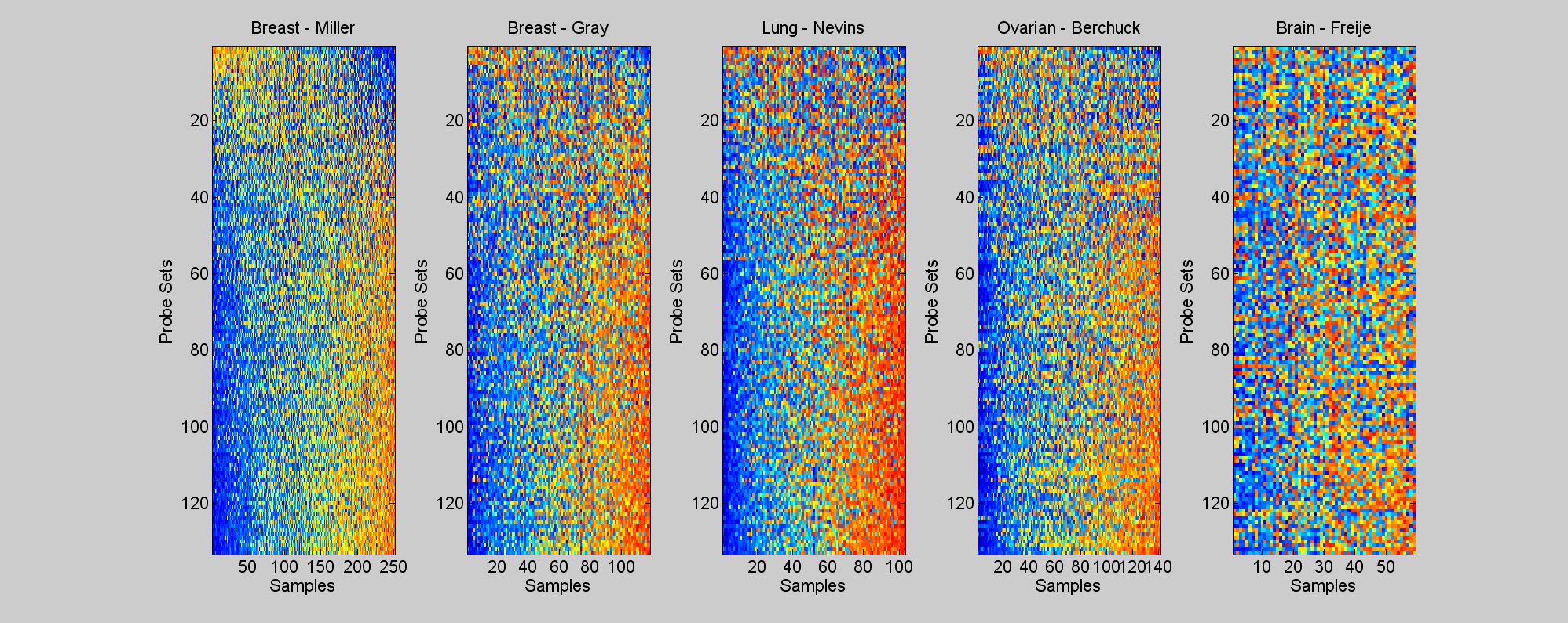

Supplement: Figure S1 — The coordinated expression of the latent factors in the five indicated cancer datasets of breast, lung, ovarian and brain cancers. (5.76 MB ZIP) [file pcbi.1000920.s001.zip › fac3.png]

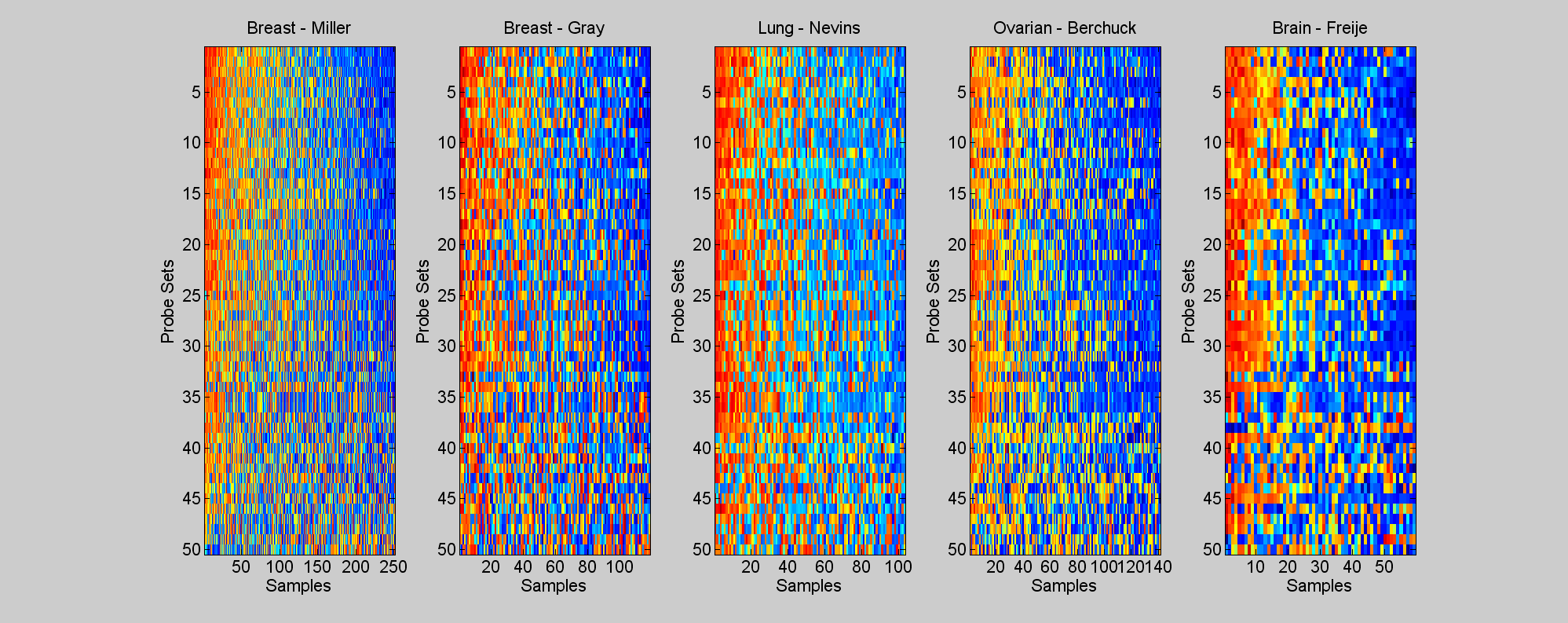

Supplement: Figure S1 — The coordinated expression of the latent factors in the five indicated cancer datasets of breast, lung, ovarian and brain cancers. (5.76 MB ZIP) [file pcbi.1000920.s001.zip › fac4.png]

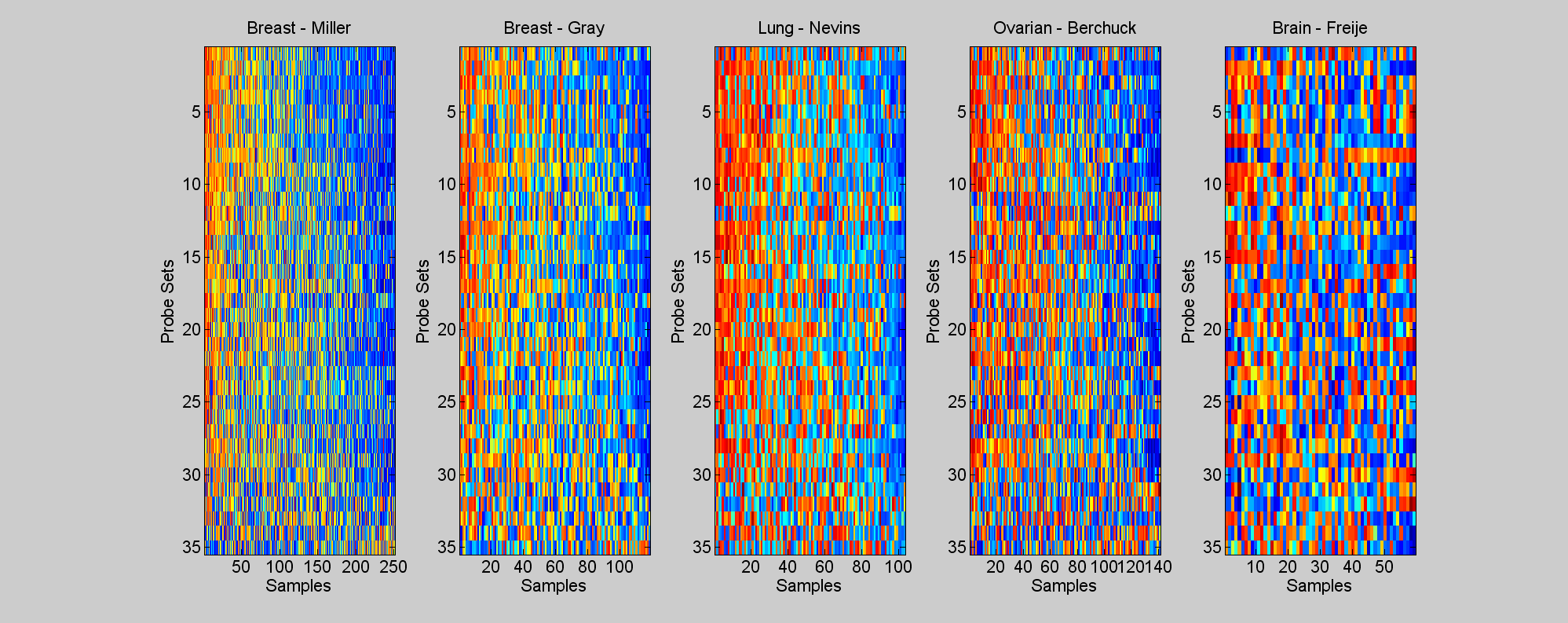

Supplement: Figure S1 — The coordinated expression of the latent factors in the five indicated cancer datasets of breast, lung, ovarian and brain cancers. (5.76 MB ZIP) [file pcbi.1000920.s001.zip › fac5.png]

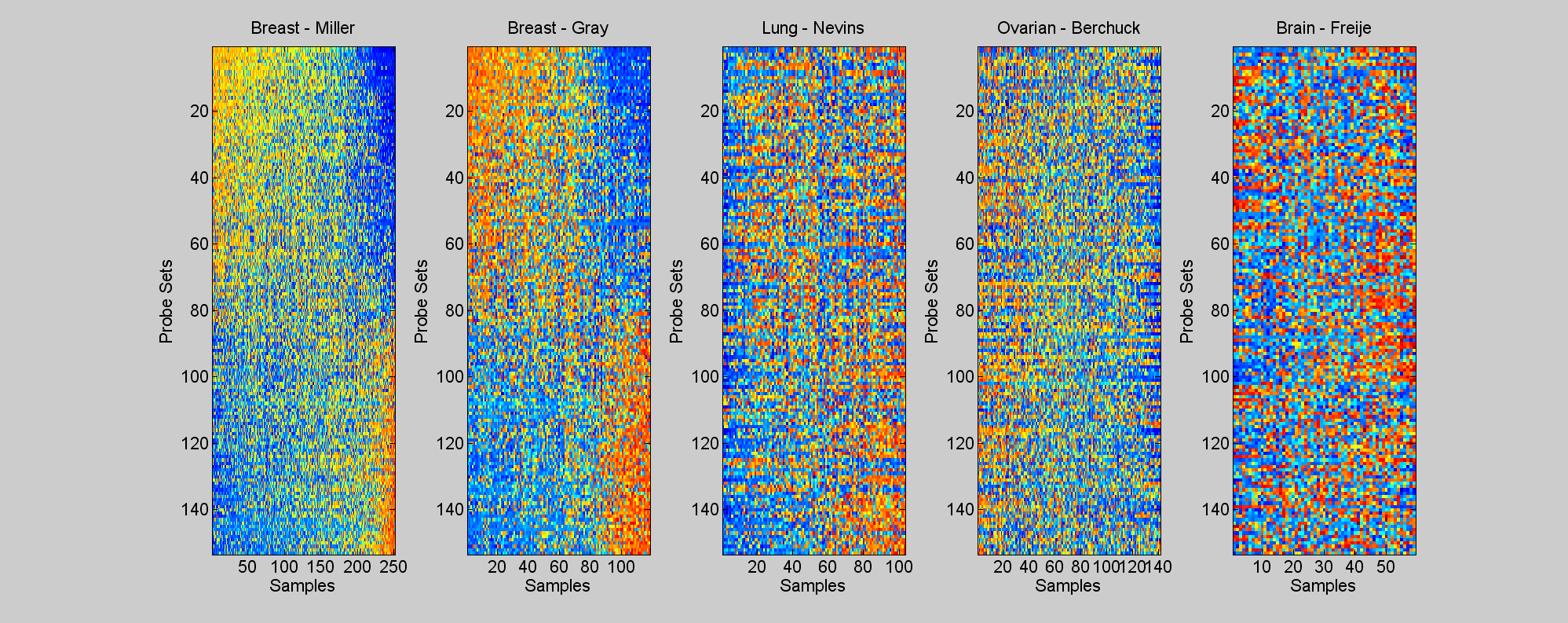

Supplement: Figure S1 — The coordinated expression of the latent factors in the five indicated cancer datasets of breast, lung, ovarian and brain cancers. (5.76 MB ZIP) [file pcbi.1000920.s001.zip › fac6.png]

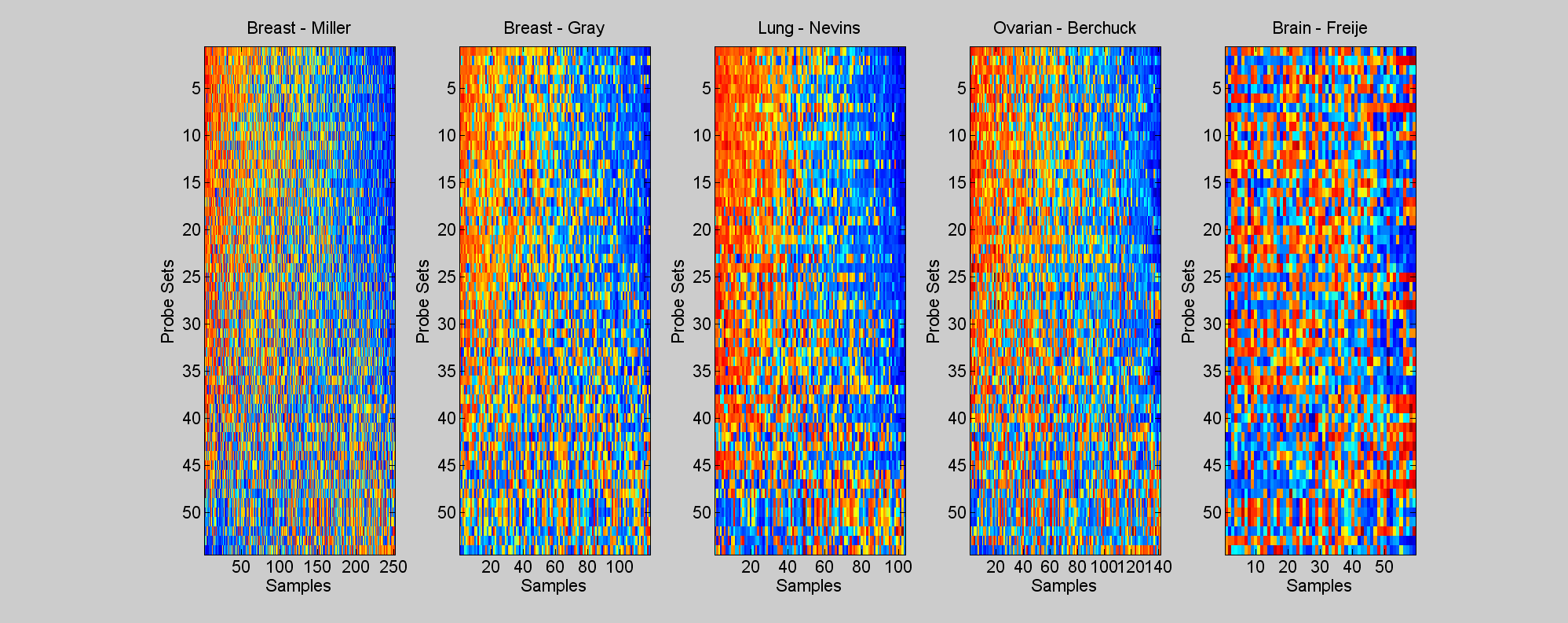

Supplement: Figure S1 — The coordinated expression of the latent factors in the five indicated cancer datasets of breast, lung, ovarian and brain cancers. (5.76 MB ZIP) [file pcbi.1000920.s001.zip › fac7.png]

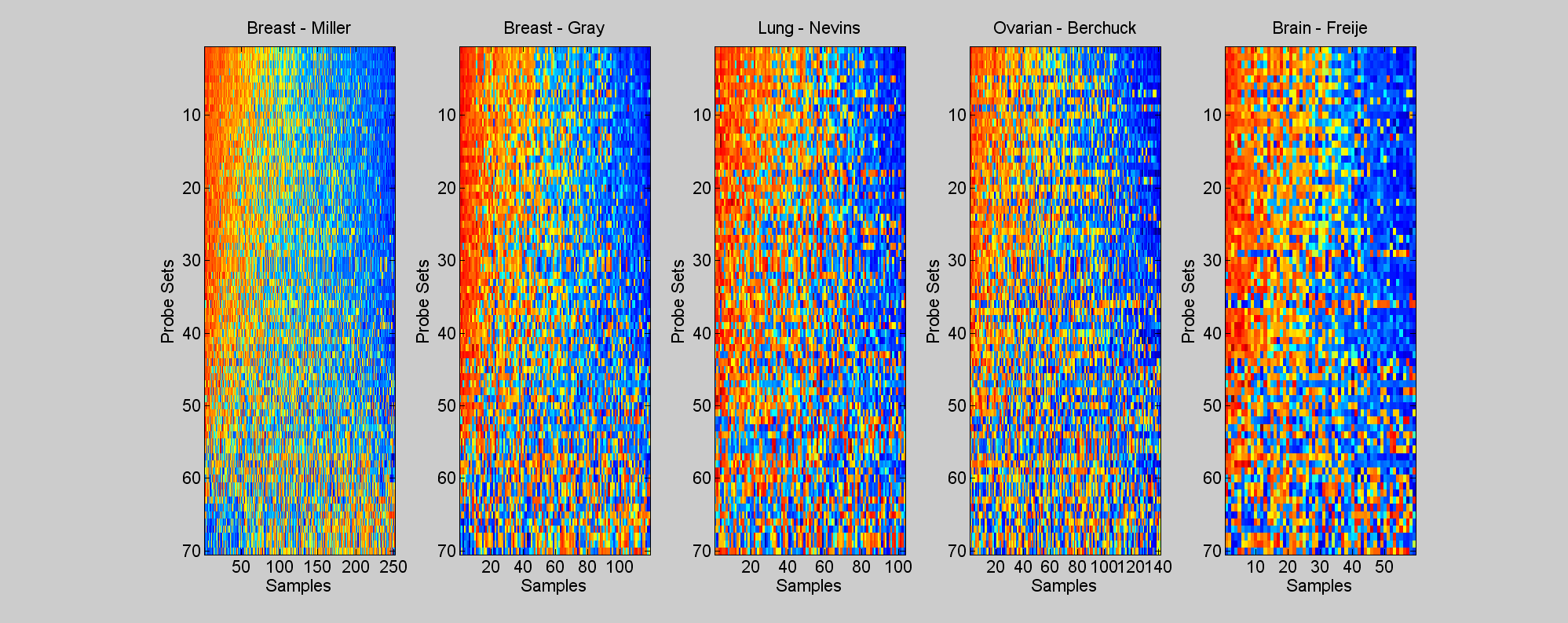

Supplement: Figure S1 — The coordinated expression of the latent factors in the five indicated cancer datasets of breast, lung, ovarian and brain cancers. (5.76 MB ZIP) [file pcbi.1000920.s001.zip › fac8.png]

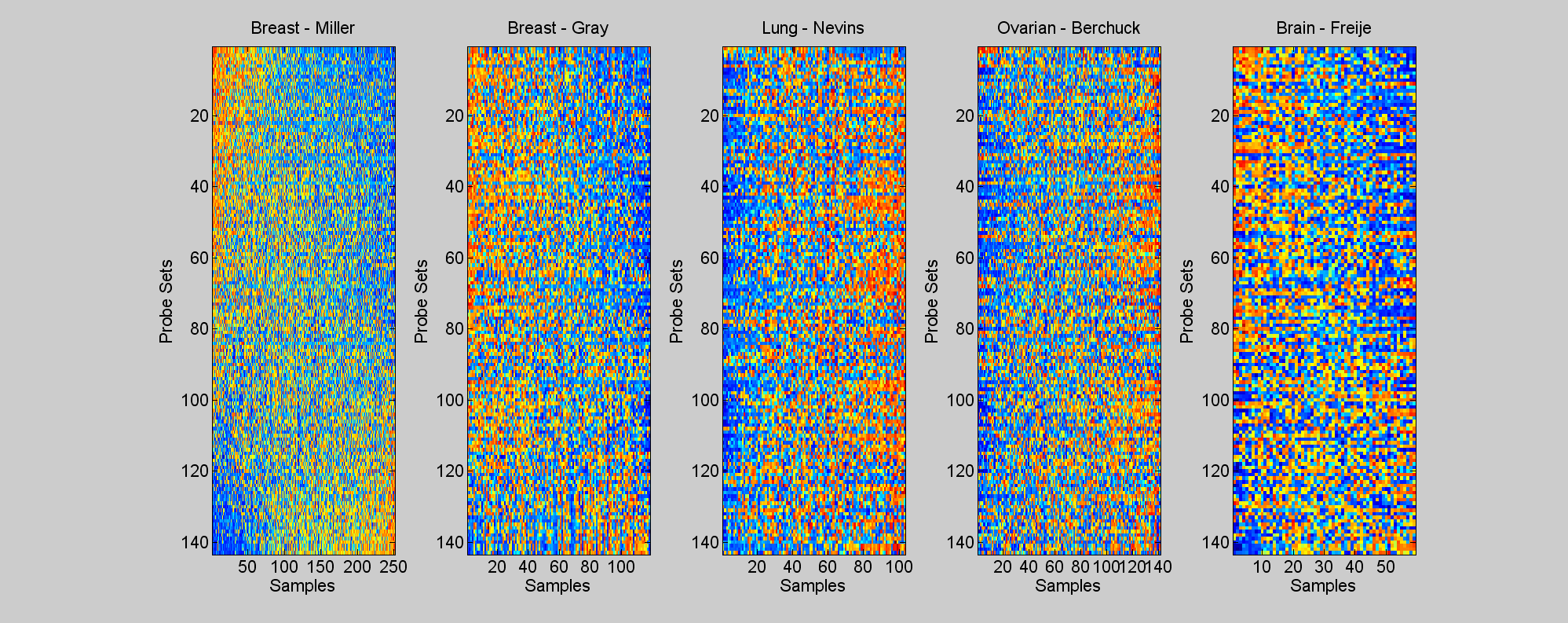

Supplement: Figure S1 — The coordinated expression of the latent factors in the five indicated cancer datasets of breast, lung, ovarian and brain cancers. (5.76 MB ZIP) [file pcbi.1000920.s001.zip › fac9.png]

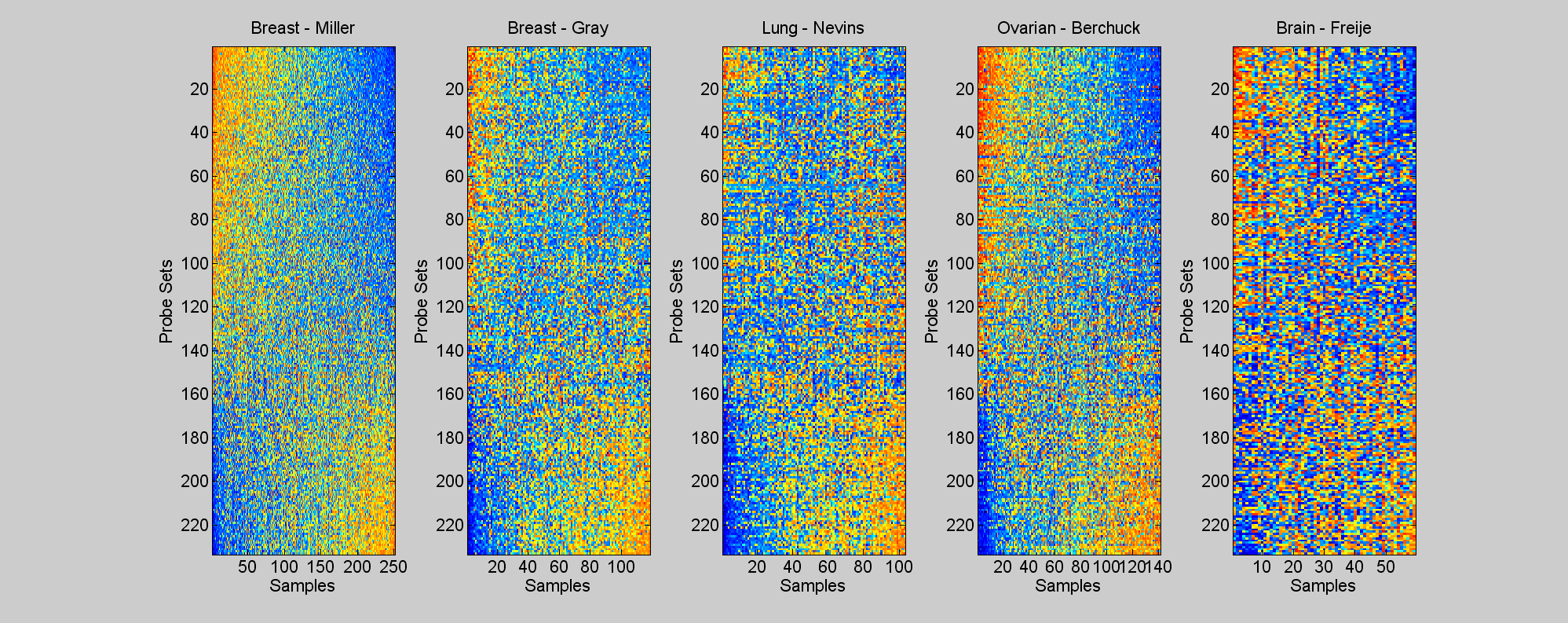

Supplement: Figure S1 — The coordinated expression of the latent factors in the five indicated cancer datasets of breast, lung, ovarian and brain cancers. (5.76 MB ZIP) [file pcbi.1000920.s001.zip › fac10.png]

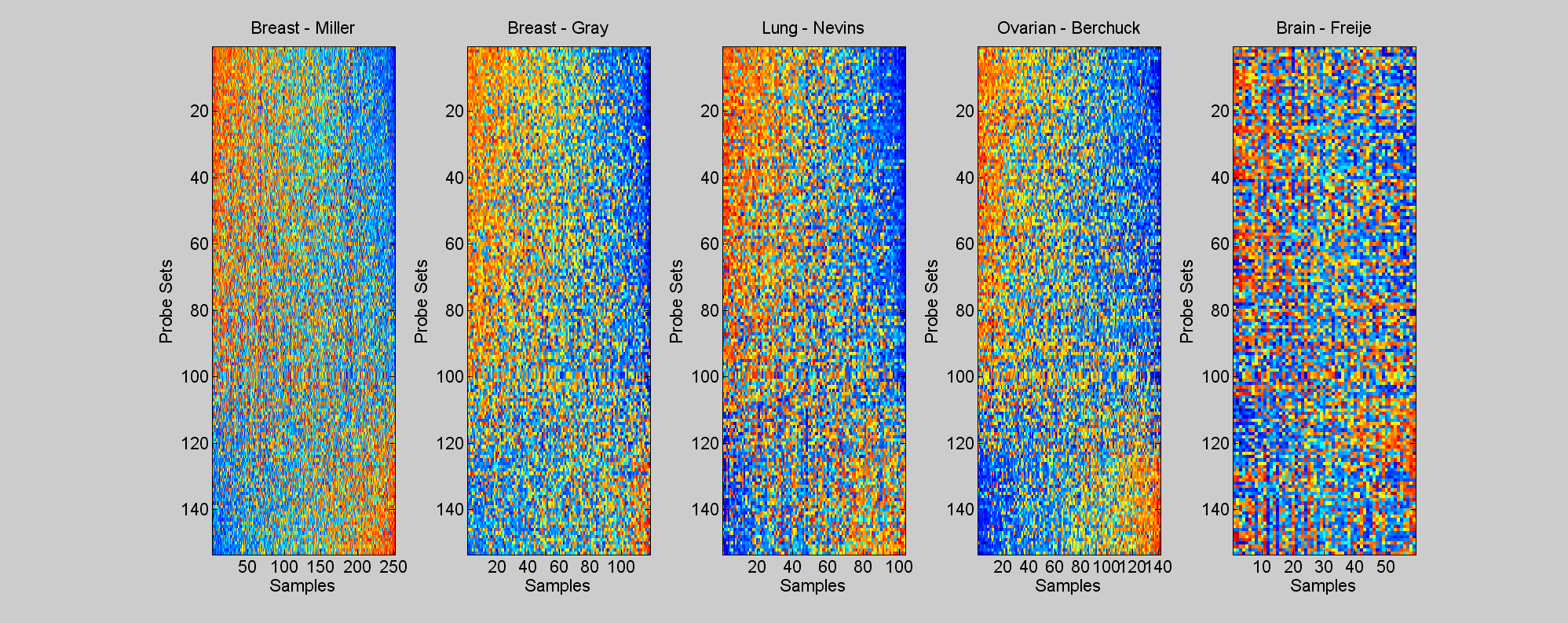

Supplement: Figure S1 — The coordinated expression of the latent factors in the five indicated cancer datasets of breast, lung, ovarian and brain cancers. (5.76 MB ZIP) [file pcbi.1000920.s001.zip › fac11.png]

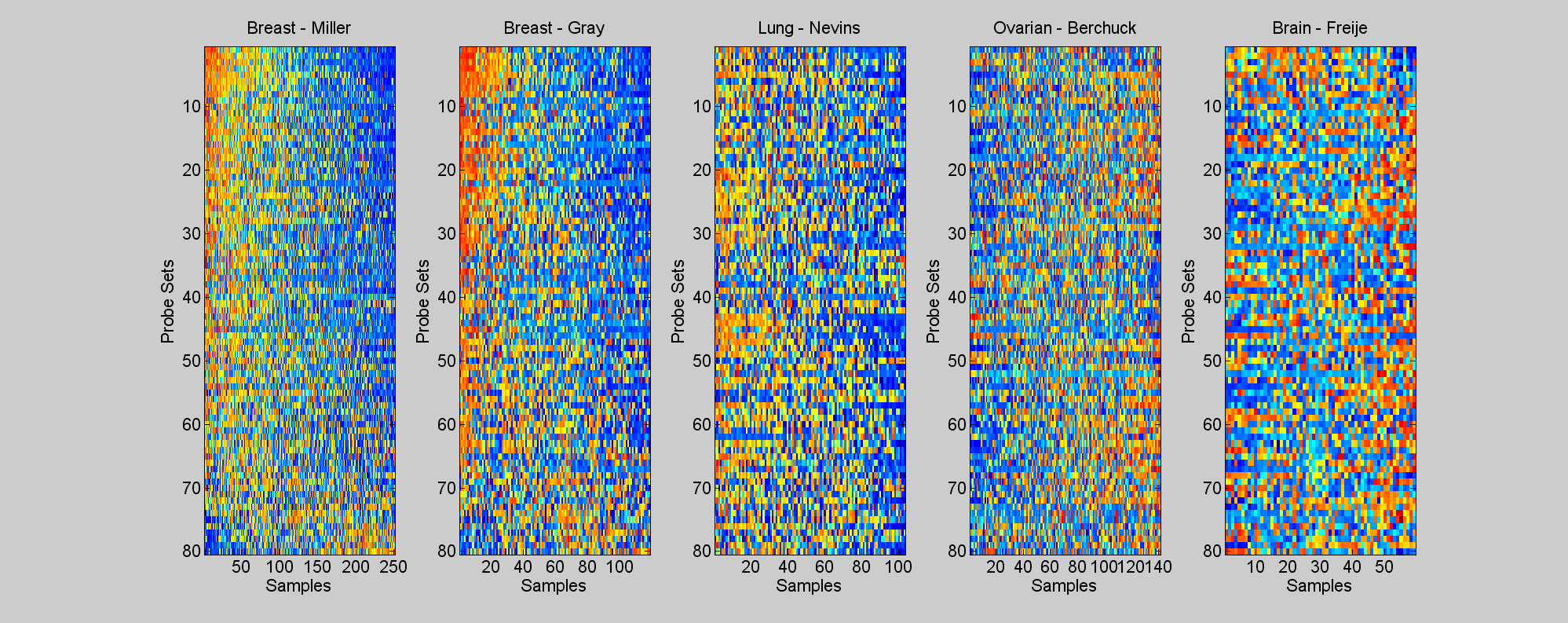

Supplement: Figure S1 — The coordinated expression of the latent factors in the five indicated cancer datasets of breast, lung, ovarian and brain cancers. (5.76 MB ZIP) [file pcbi.1000920.s001.zip › fac12.png]

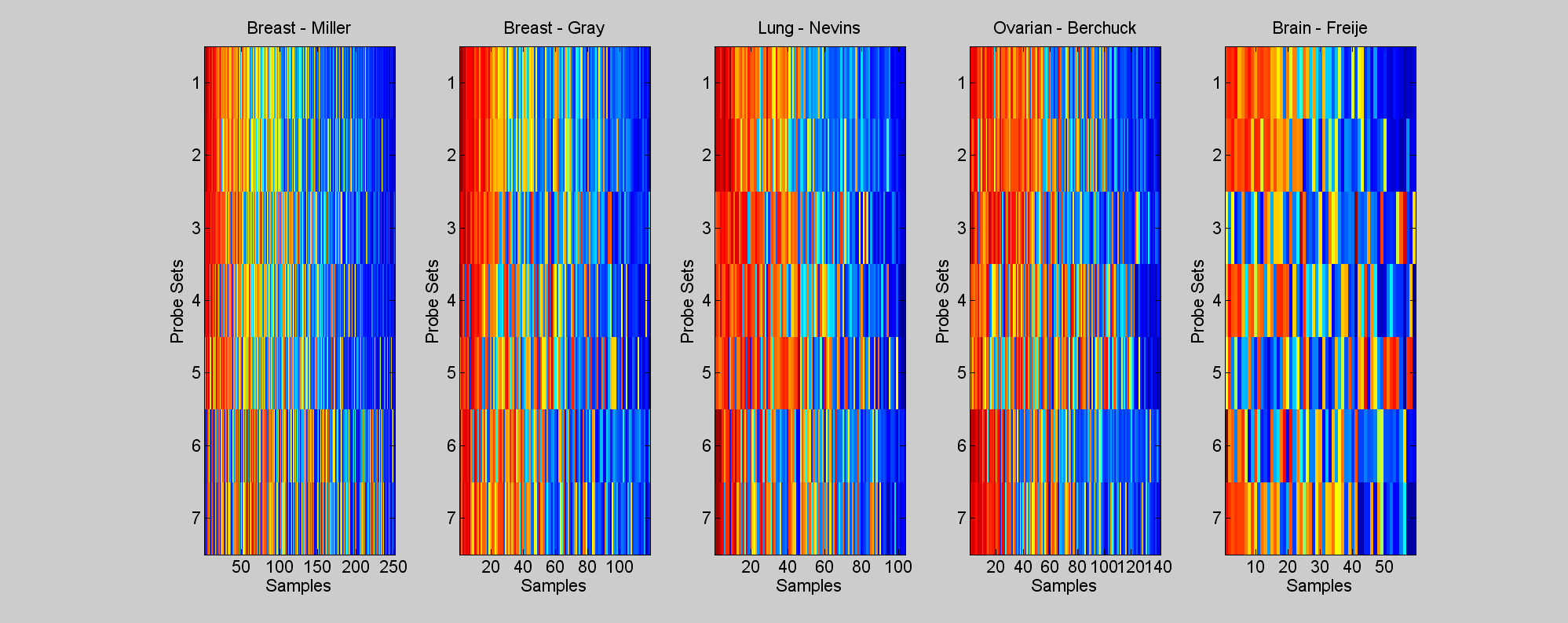

Supplement: Figure S1 — The coordinated expression of the latent factors in the five indicated cancer datasets of breast, lung, ovarian and brain cancers. (5.76 MB ZIP) [file pcbi.1000920.s001.zip › fac13.png]

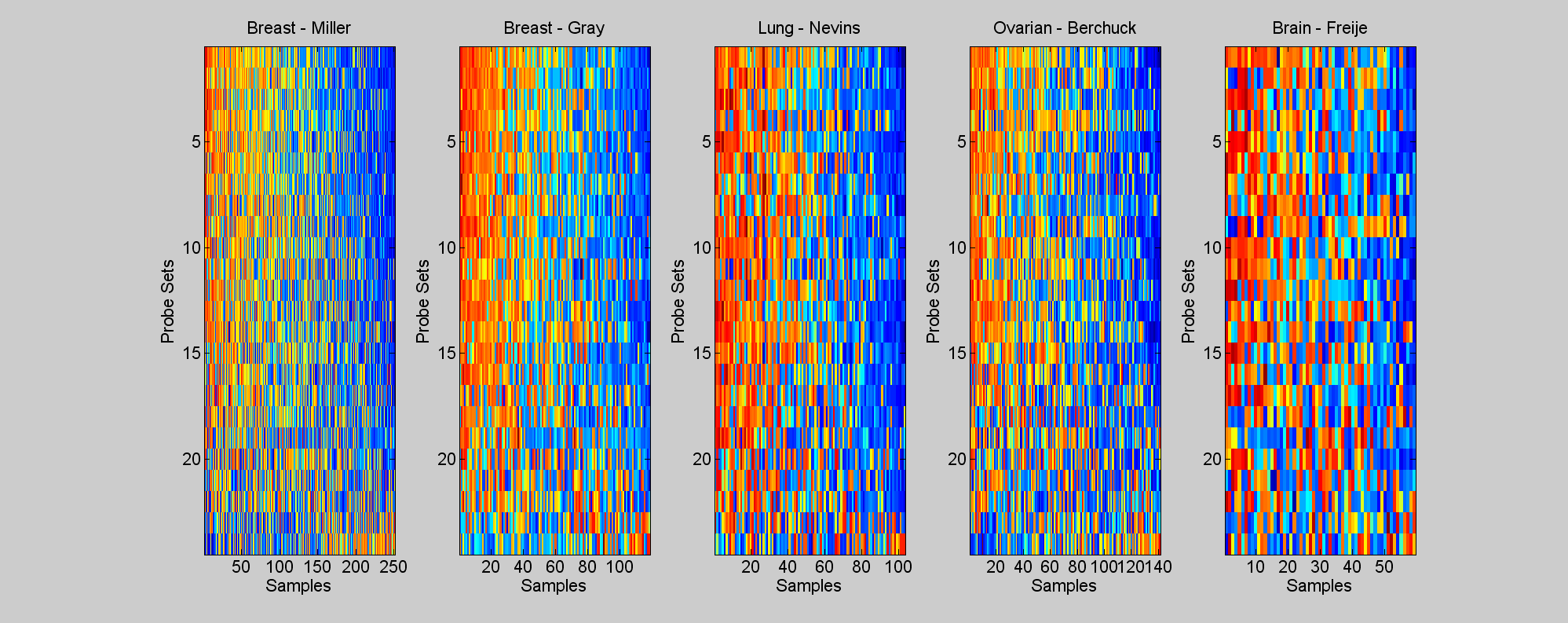

Supplement: Figure S1 — The coordinated expression of the latent factors in the five indicated cancer datasets of breast, lung, ovarian and brain cancers. (5.76 MB ZIP) [file pcbi.1000920.s001.zip › fac14.png]

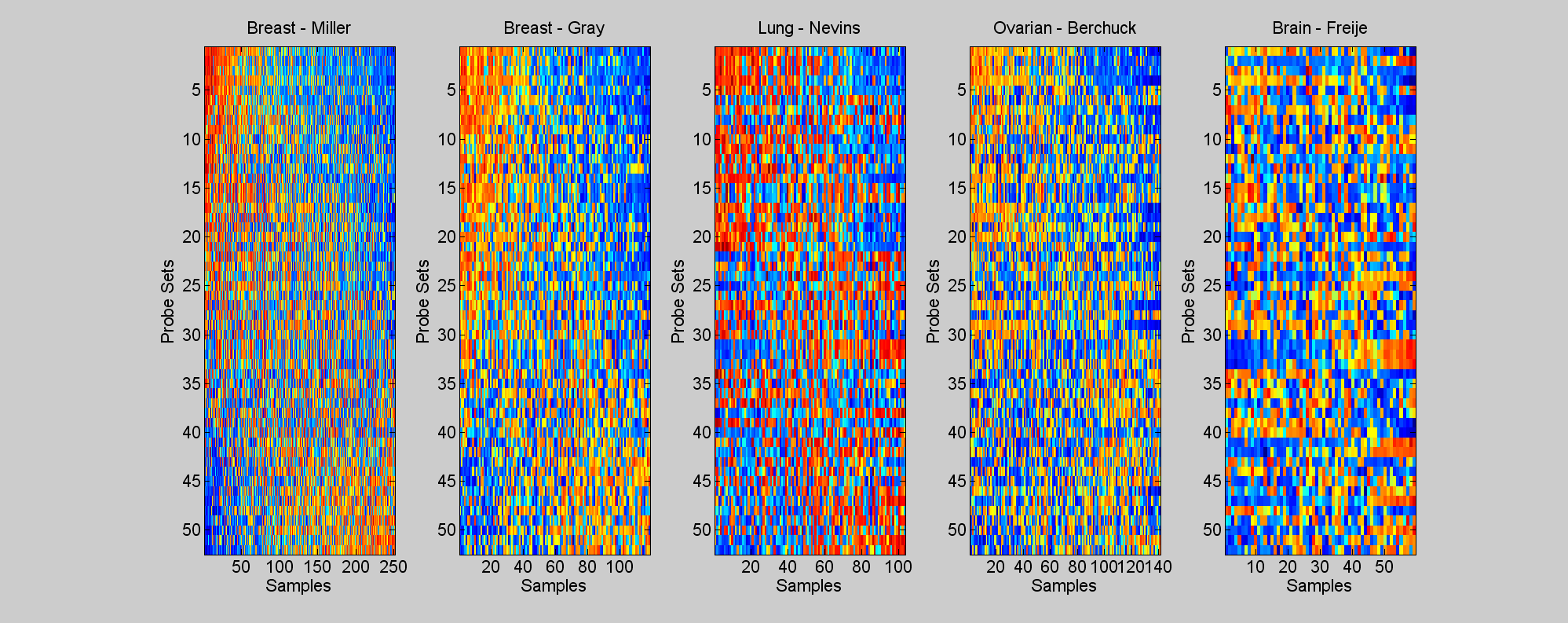

Supplement: Figure S1 — The coordinated expression of the latent factors in the five indicated cancer datasets of breast, lung, ovarian and brain cancers. (5.76 MB ZIP) [file pcbi.1000920.s001.zip › fac15.png]

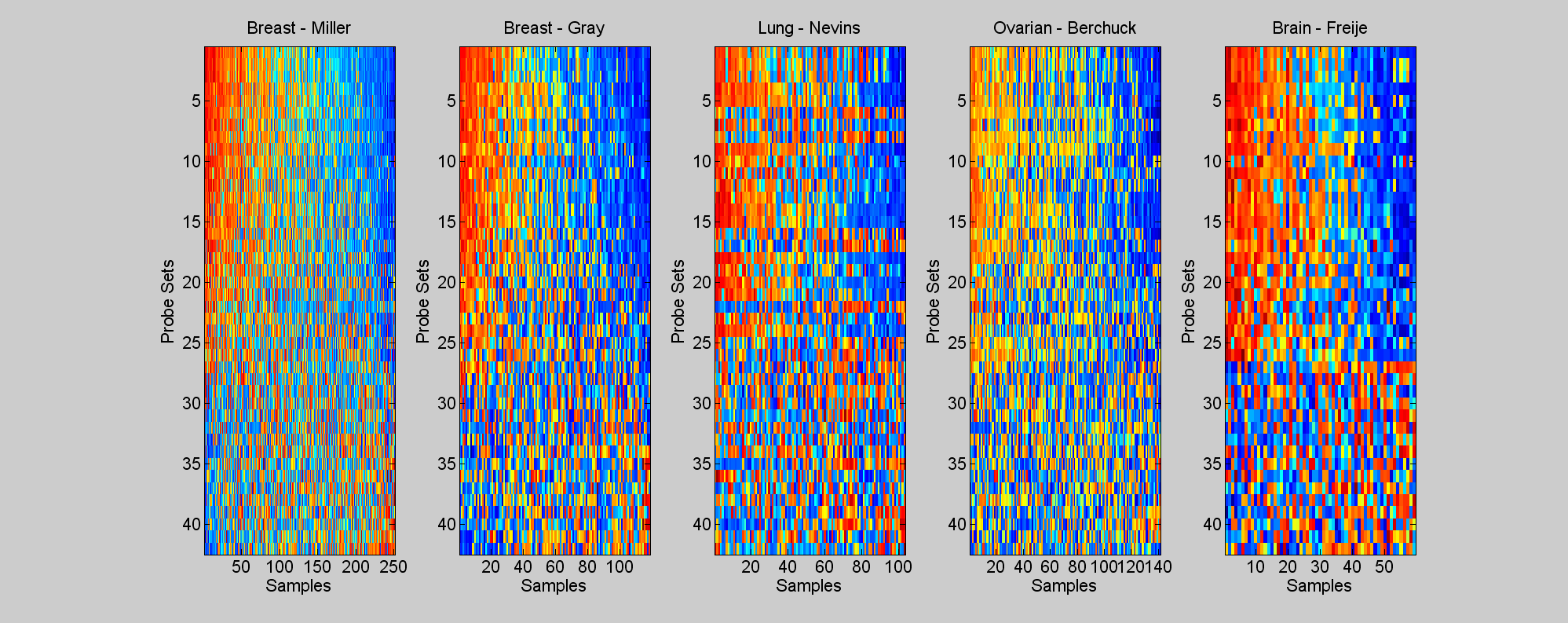

Supplement: Figure S1 — The coordinated expression of the latent factors in the five indicated cancer datasets of breast, lung, ovarian and brain cancers. (5.76 MB ZIP) [file pcbi.1000920.s001.zip › fac16.png]

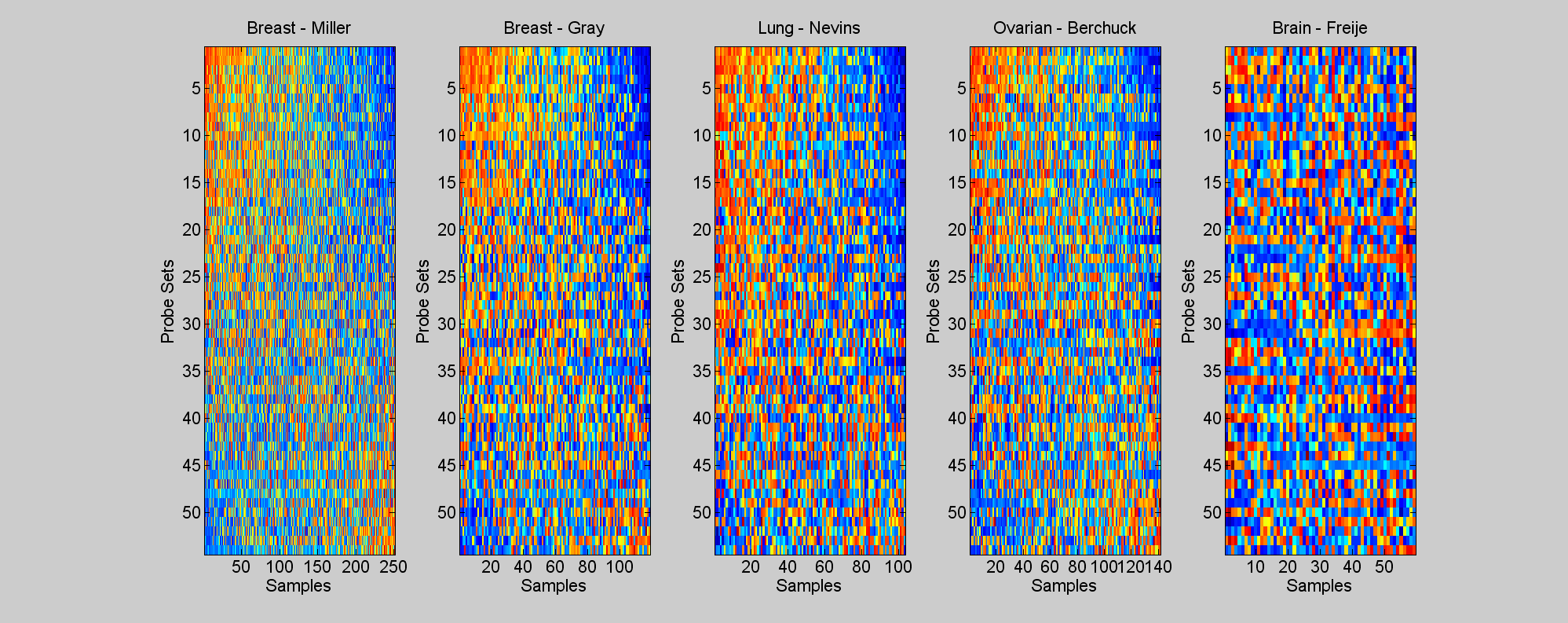

Supplement: Figure S1 — The coordinated expression of the latent factors in the five indicated cancer datasets of breast, lung, ovarian and brain cancers. (5.76 MB ZIP) [file pcbi.1000920.s001.zip › fac17.png]

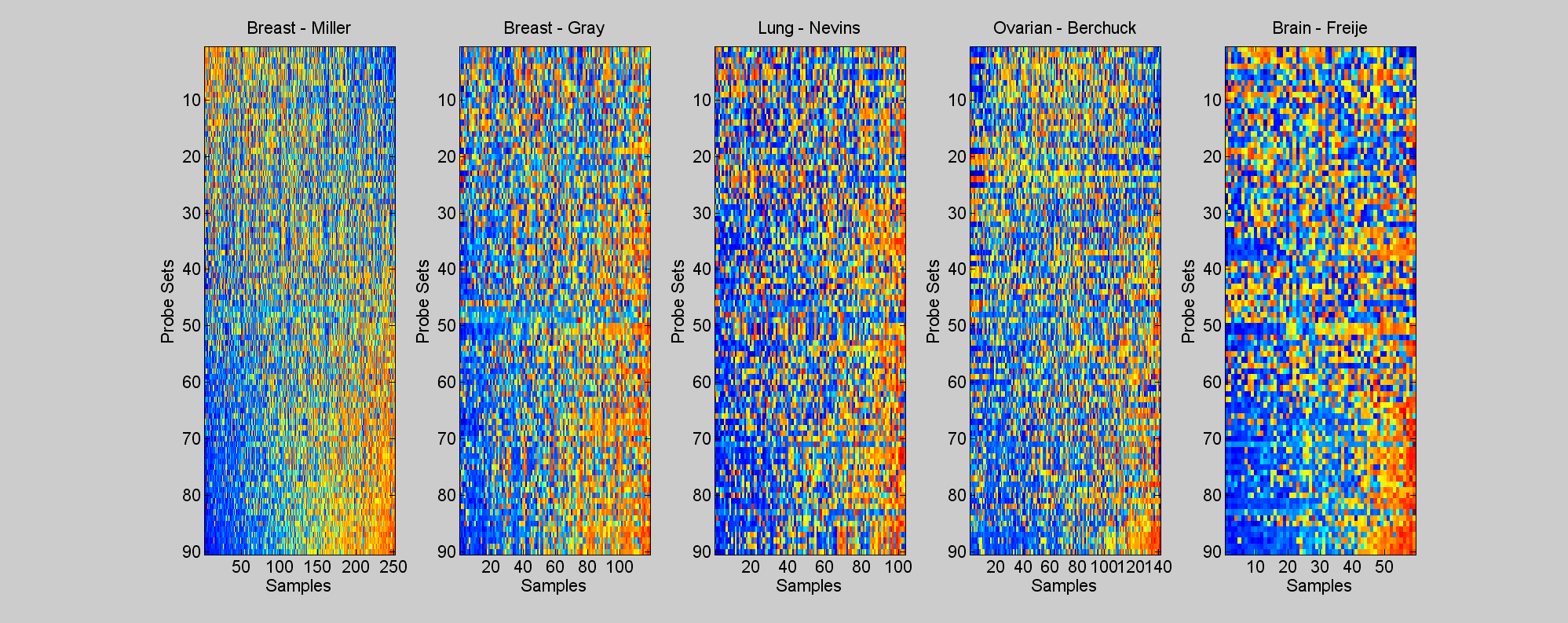

Supplement: Figure S1 — The coordinated expression of the latent factors in the five indicated cancer datasets of breast, lung, ovarian and brain cancers. (5.76 MB ZIP) [file pcbi.1000920.s001.zip › fac18.png]

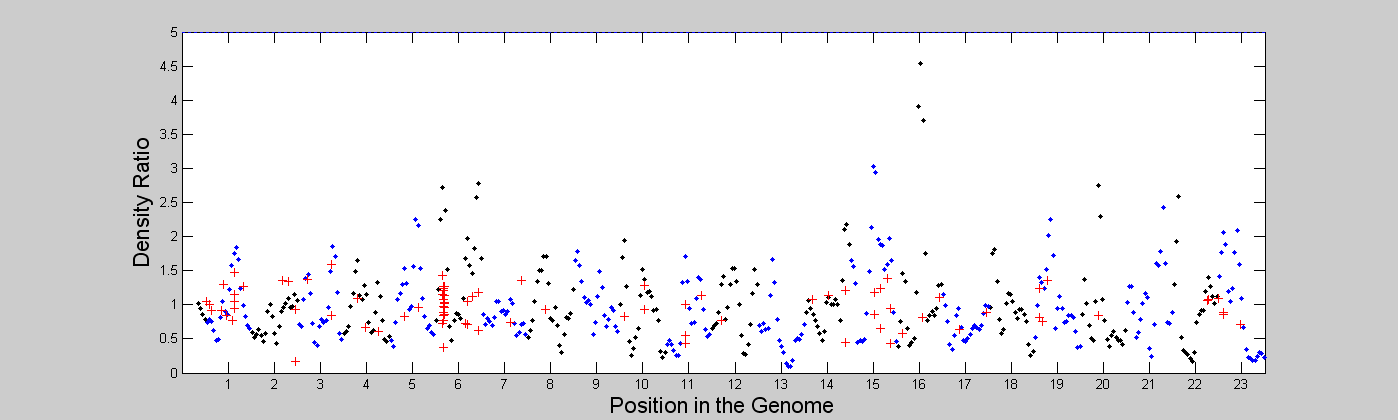

Supplement: Figure S2 — The local enrichment ratios (LER) for chromosomal enrichment of the latent factors. (0.86 MB ZIP) [file pcbi.1000920.s002.zip › fac8.png]

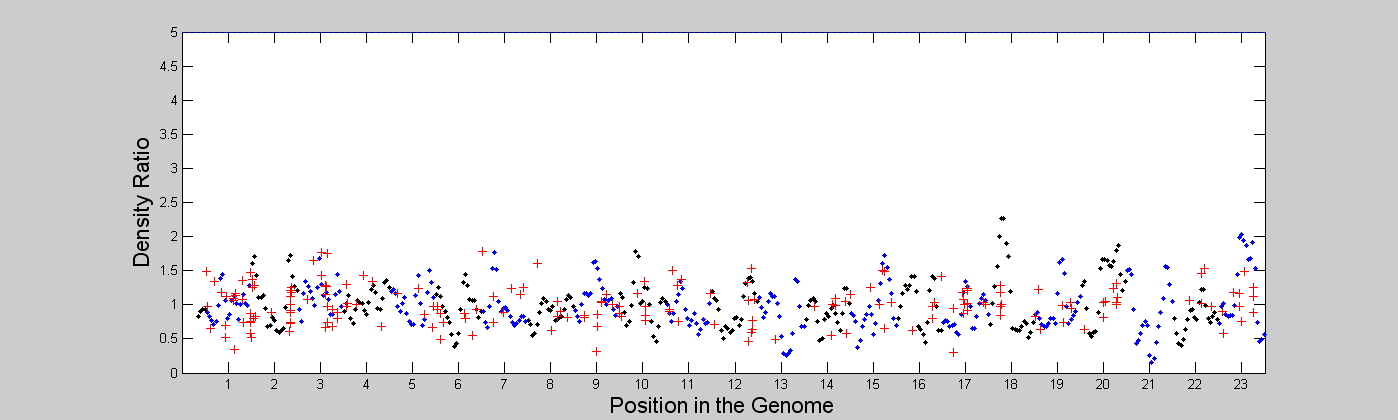

Supplement: Figure S2 — The local enrichment ratios (LER) for chromosomal enrichment of the latent factors. (0.86 MB ZIP) [file pcbi.1000920.s002.zip › fac9.png]

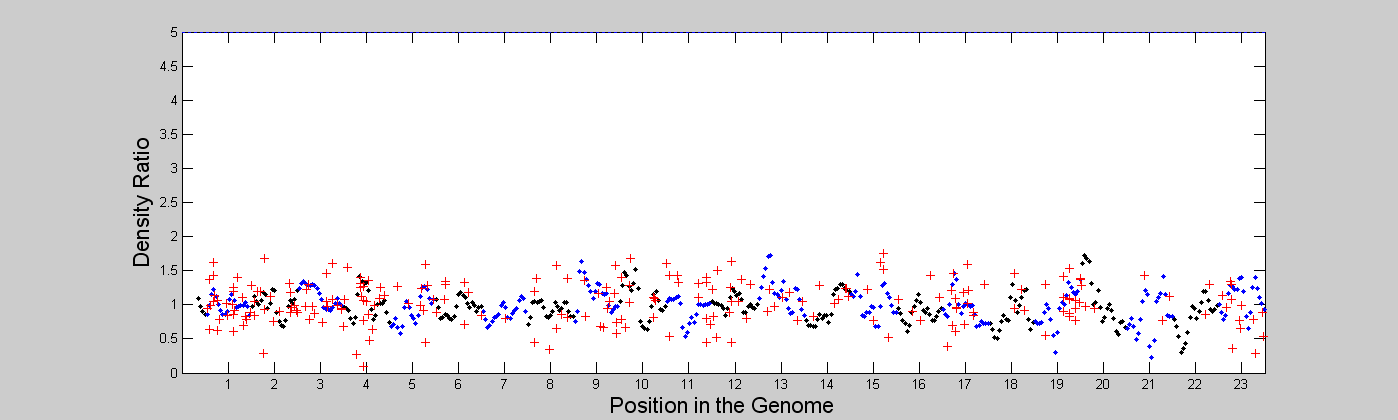

Supplement: Figure S2 — The local enrichment ratios (LER) for chromosomal enrichment of the latent factors. (0.86 MB ZIP) [file pcbi.1000920.s002.zip › fac10.png]

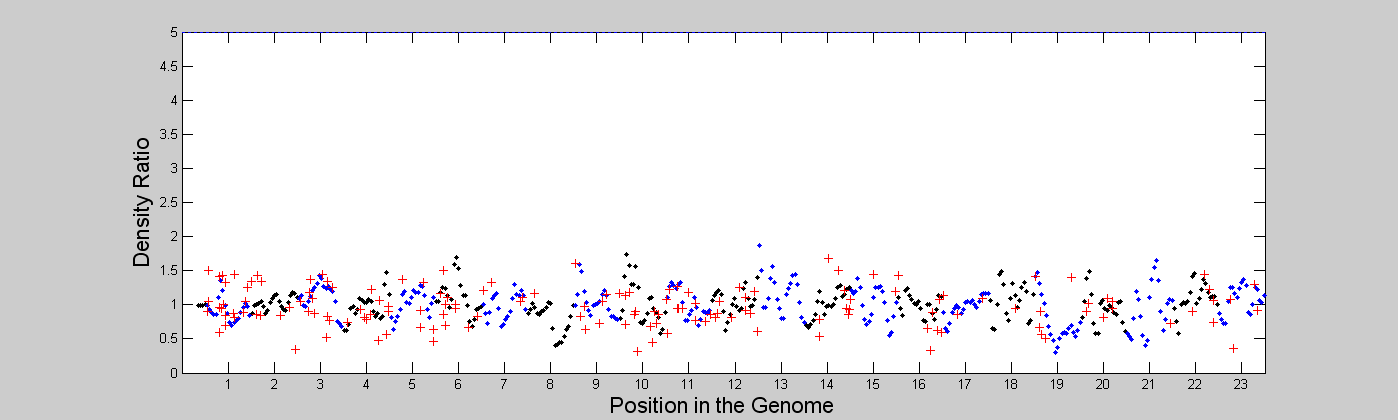

Supplement: Figure S2 — The local enrichment ratios (LER) for chromosomal enrichment of the latent factors. (0.86 MB ZIP) [file pcbi.1000920.s002.zip › fac11.png]

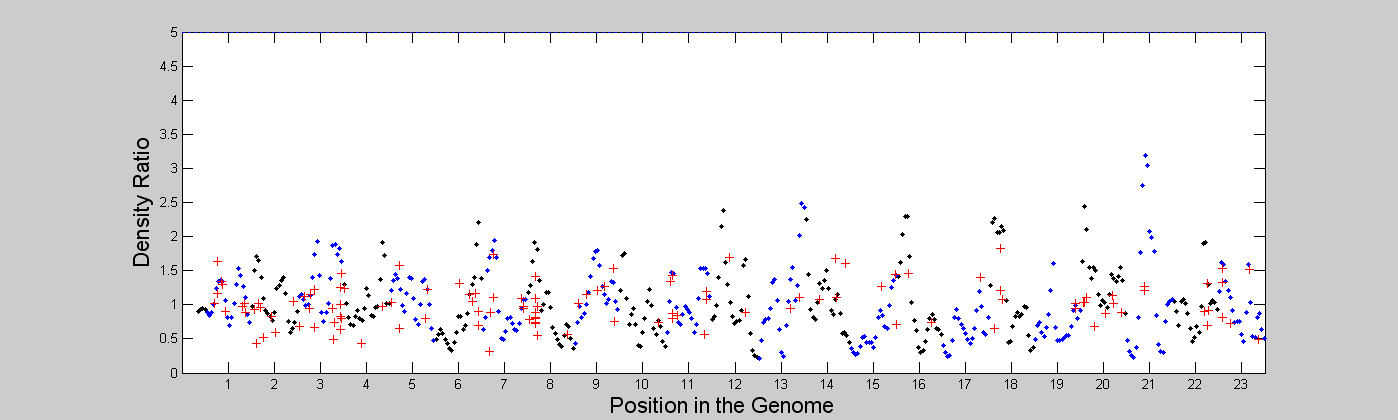

Supplement: Figure S2 — The local enrichment ratios (LER) for chromosomal enrichment of the latent factors. (0.86 MB ZIP) [file pcbi.1000920.s002.zip › fac12.png]

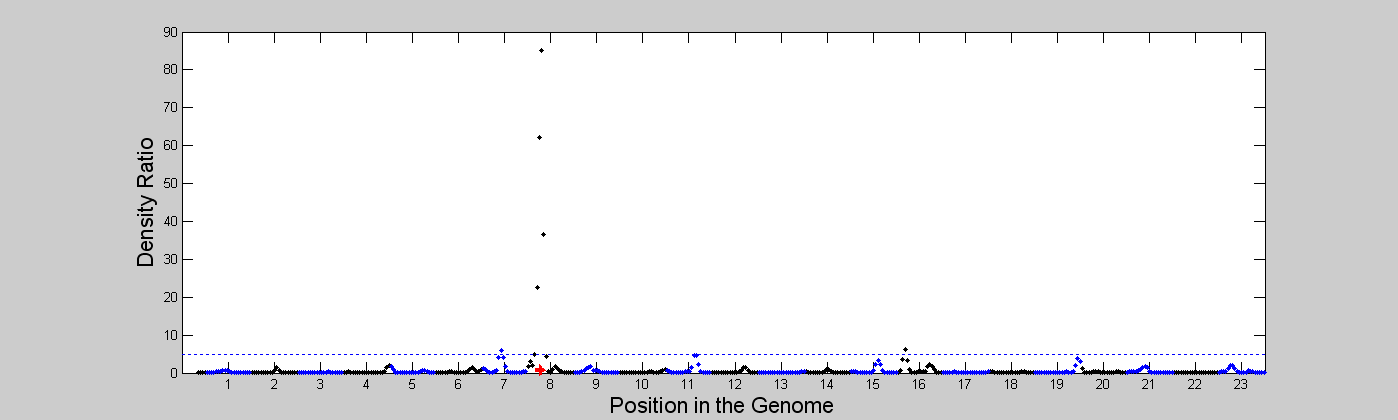

Supplement: Figure S2 — The local enrichment ratios (LER) for chromosomal enrichment of the latent factors. (0.86 MB ZIP) [file pcbi.1000920.s002.zip › fac13.png]

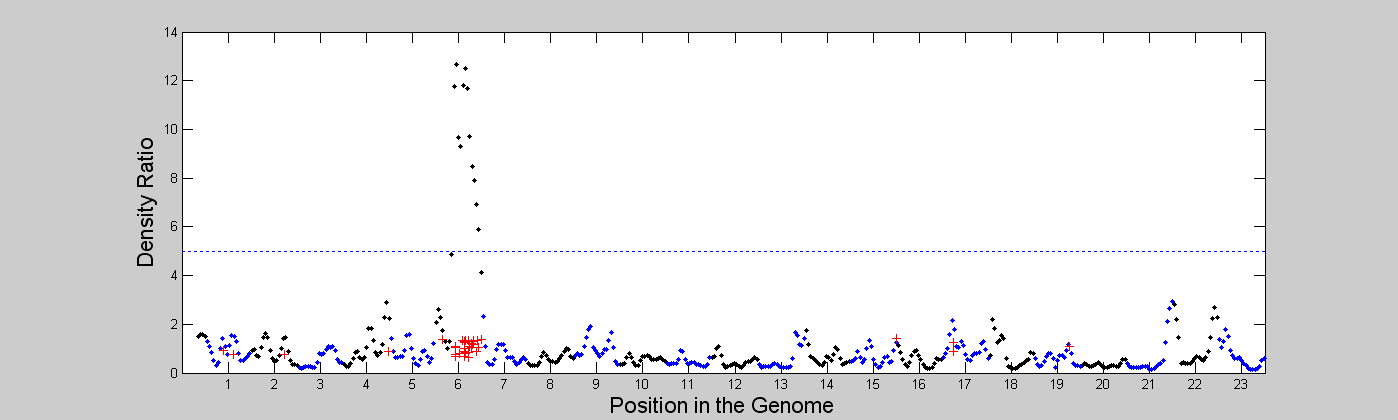

Supplement: Figure S2 — The local enrichment ratios (LER) for chromosomal enrichment of the latent factors. (0.86 MB ZIP) [file pcbi.1000920.s002.zip › fac14.png]

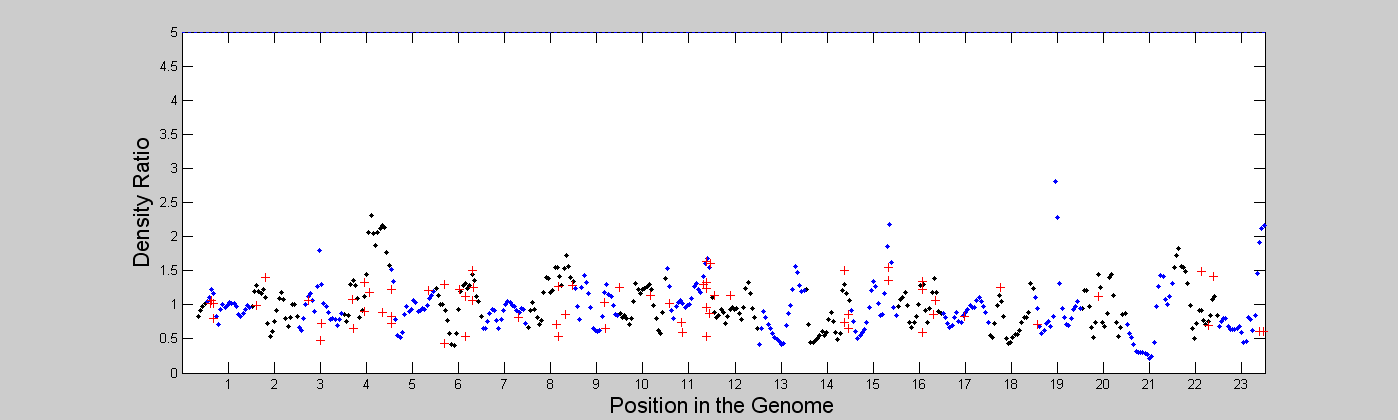

Supplement: Figure S2 — The local enrichment ratios (LER) for chromosomal enrichment of the latent factors. (0.86 MB ZIP) [file pcbi.1000920.s002.zip › fac15.png]

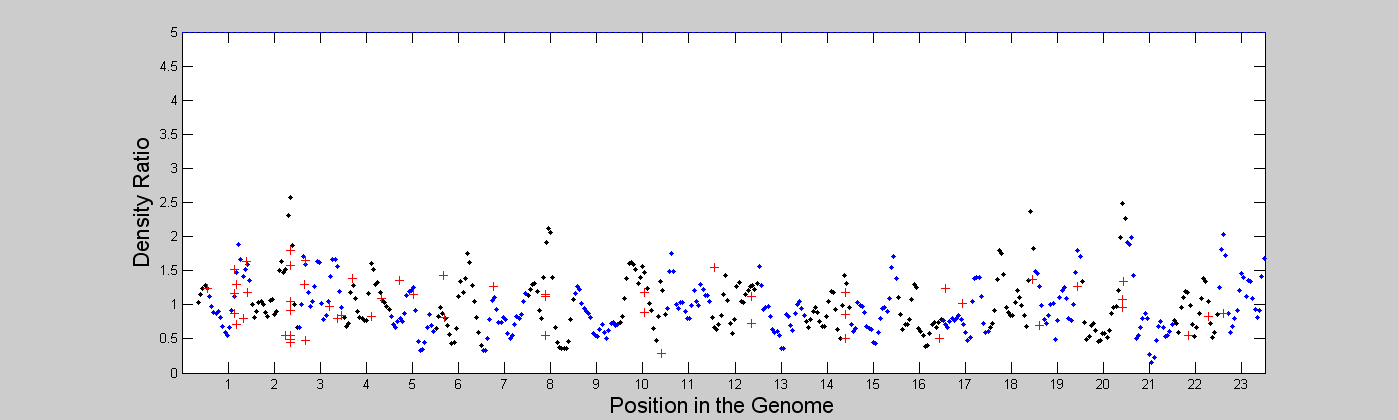

Supplement: Figure S2 — The local enrichment ratios (LER) for chromosomal enrichment of the latent factors. (0.86 MB ZIP) [file pcbi.1000920.s002.zip › fac16.png]

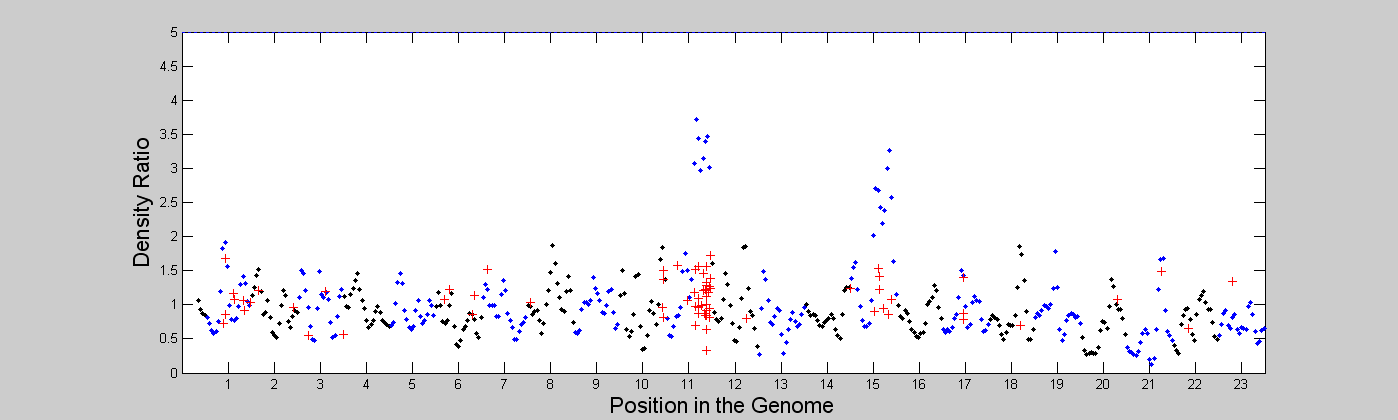

Supplement: Figure S2 — The local enrichment ratios (LER) for chromosomal enrichment of the latent factors. (0.86 MB ZIP) [file pcbi.1000920.s002.zip › fac17.png]

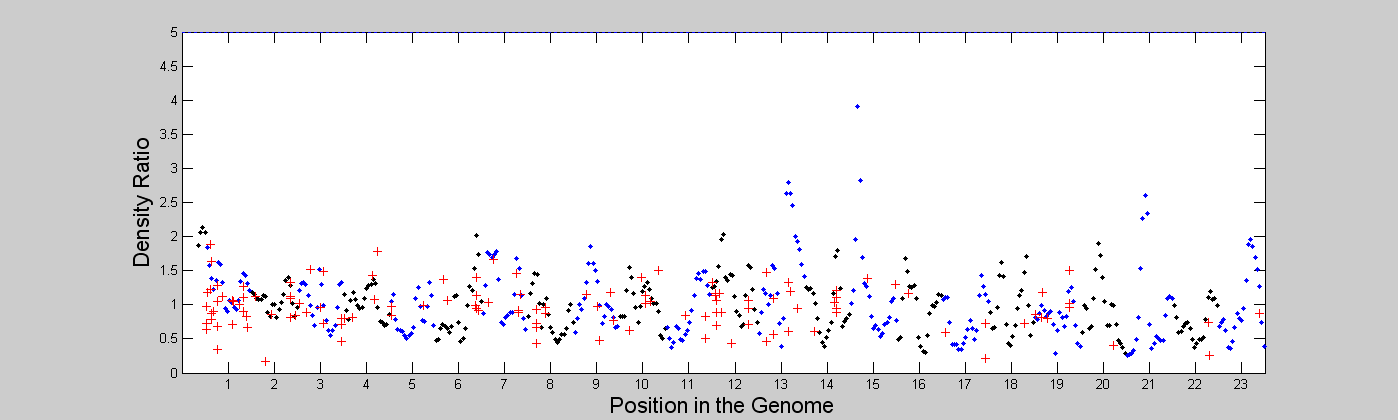

Supplement: Figure S2 — The local enrichment ratios (LER) for chromosomal enrichment of the latent factors. (0.86 MB ZIP) [file pcbi.1000920.s002.zip › fac18.png]

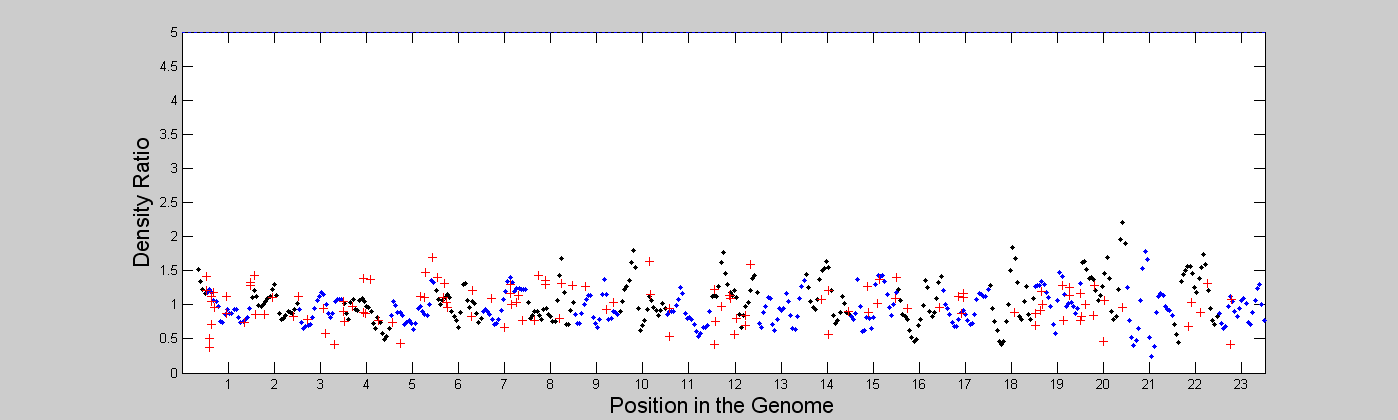

Supplement: Figure S2 — The local enrichment ratios (LER) for chromosomal enrichment of the latent factors. (0.86 MB ZIP) [file pcbi.1000920.s002.zip › fac19.png]

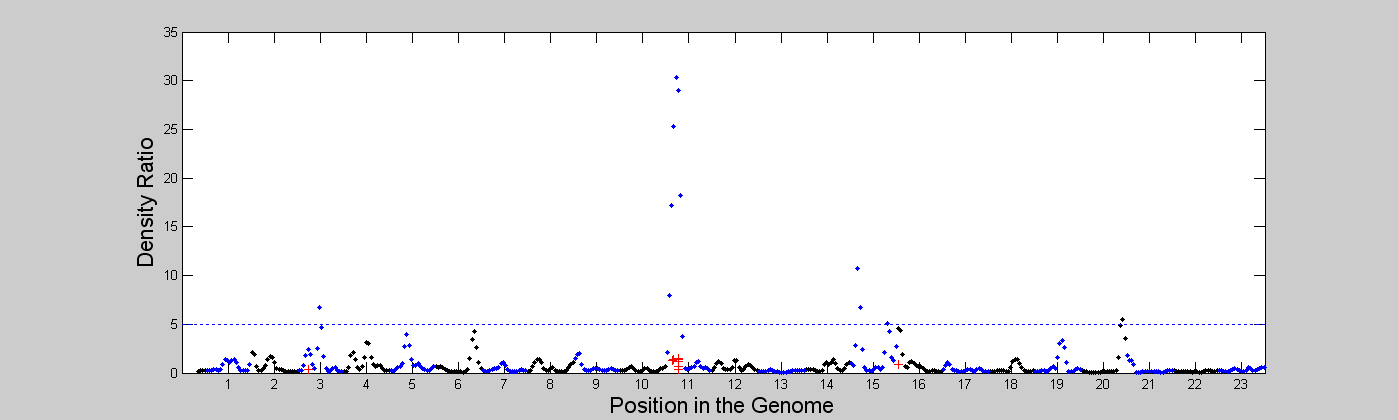

Supplement: Figure S2 — The local enrichment ratios (LER) for chromosomal enrichment of the latent factors. (0.86 MB ZIP) [file pcbi.1000920.s002.zip › fac20.png]

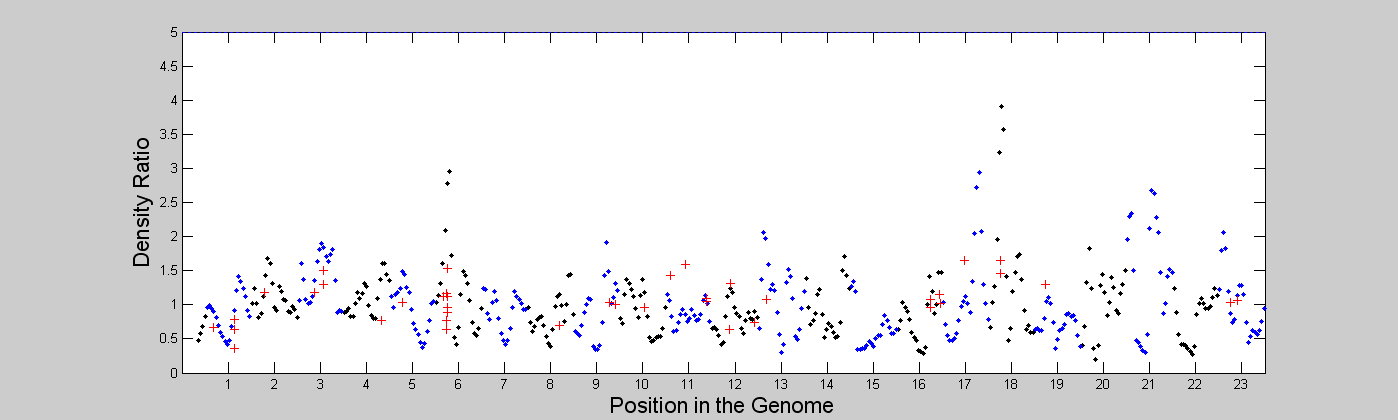

Supplement: Figure S2 — The local enrichment ratios (LER) for chromosomal enrichment of the latent factors. (0.86 MB ZIP) [file pcbi.1000920.s002.zip › fac21.png]

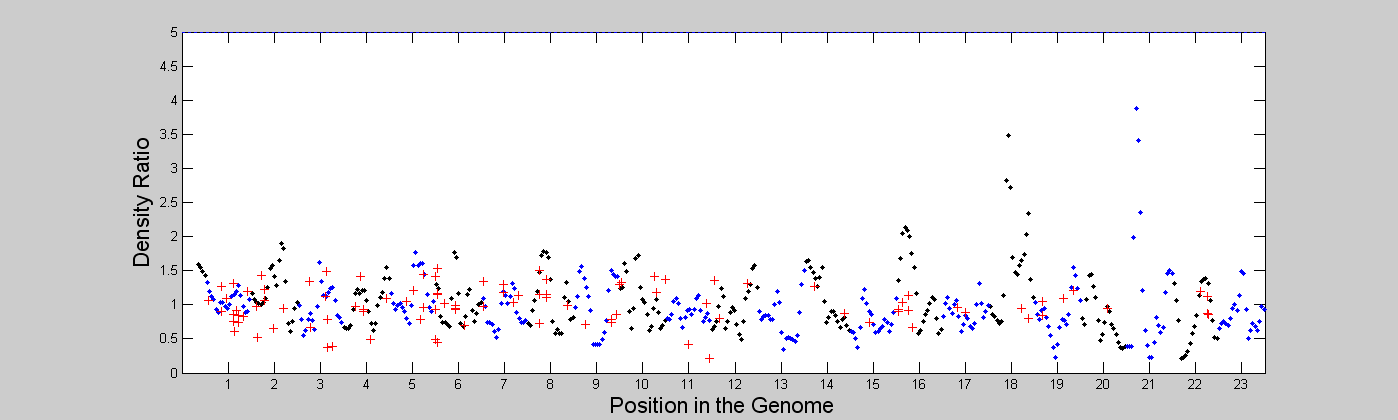

Supplement: Figure S2 — The local enrichment ratios (LER) for chromosomal enrichment of the latent factors. (0.86 MB ZIP) [file pcbi.1000920.s002.zip › fac22.png]

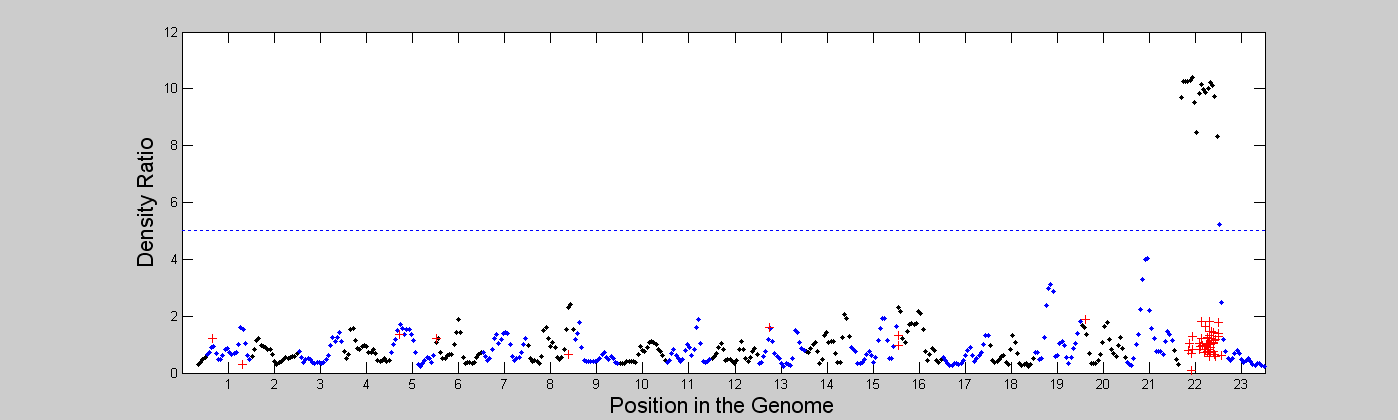

Supplement: Figure S2 — The local enrichment ratios (LER) for chromosomal enrichment of the latent factors. (0.86 MB ZIP) [file pcbi.1000920.s002.zip › fac23.png]

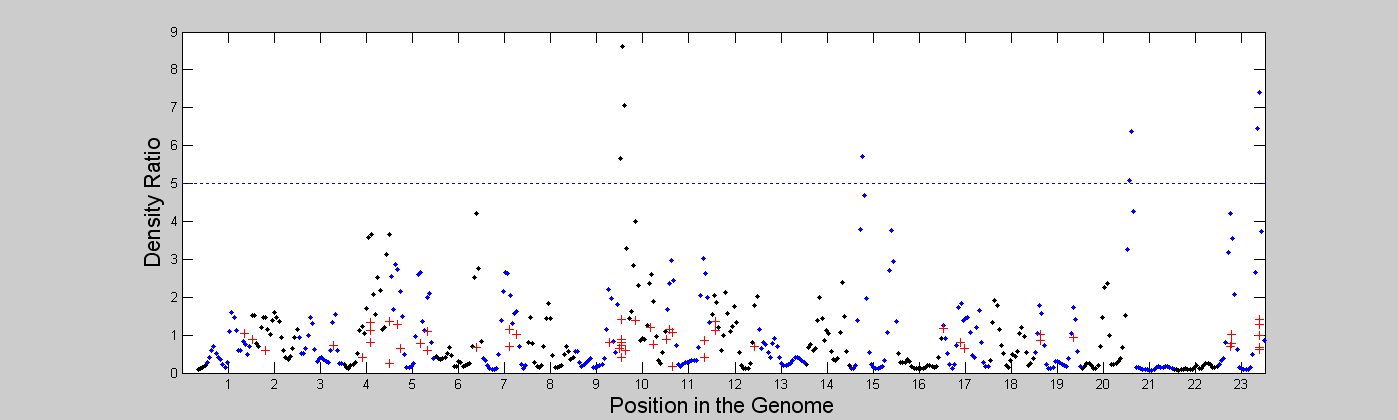

Supplement: Figure S2 — The local enrichment ratios (LER) for chromosomal enrichment of the latent factors. (0.86 MB ZIP) [file pcbi.1000920.s002.zip › fac24.png]

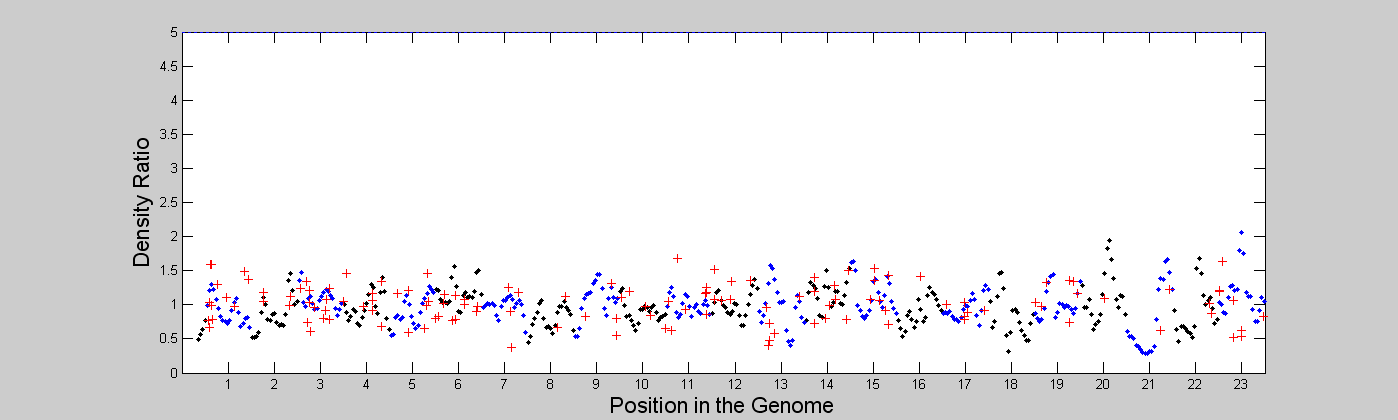

Supplement: Figure S2 — The local enrichment ratios (LER) for chromosomal enrichment of the latent factors. (0.86 MB ZIP) [file pcbi.1000920.s002.zip › fac25.png]

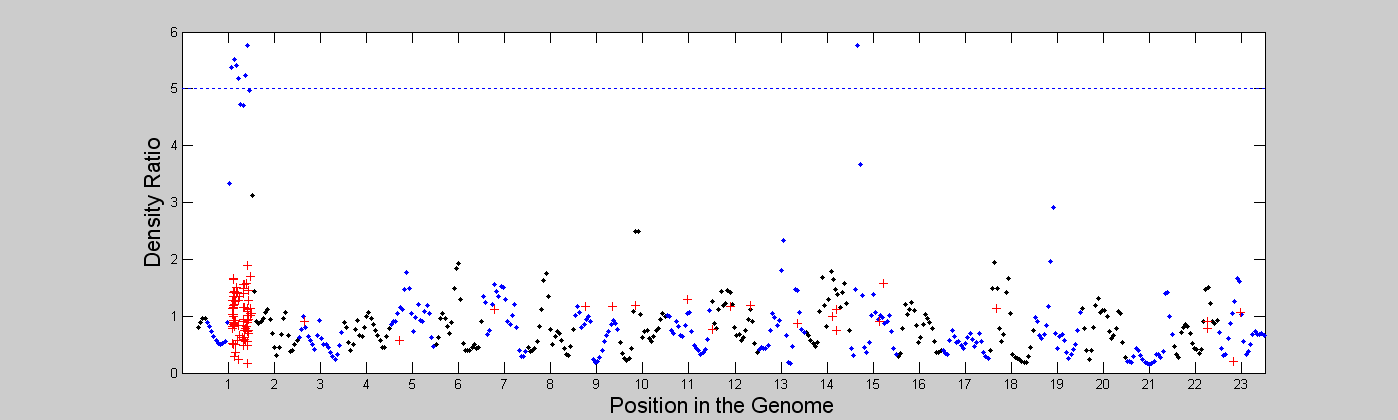

Supplement: Figure S2 — The local enrichment ratios (LER) for chromosomal enrichment of the latent factors. (0.86 MB ZIP) [file pcbi.1000920.s002.zip › fac26.png]

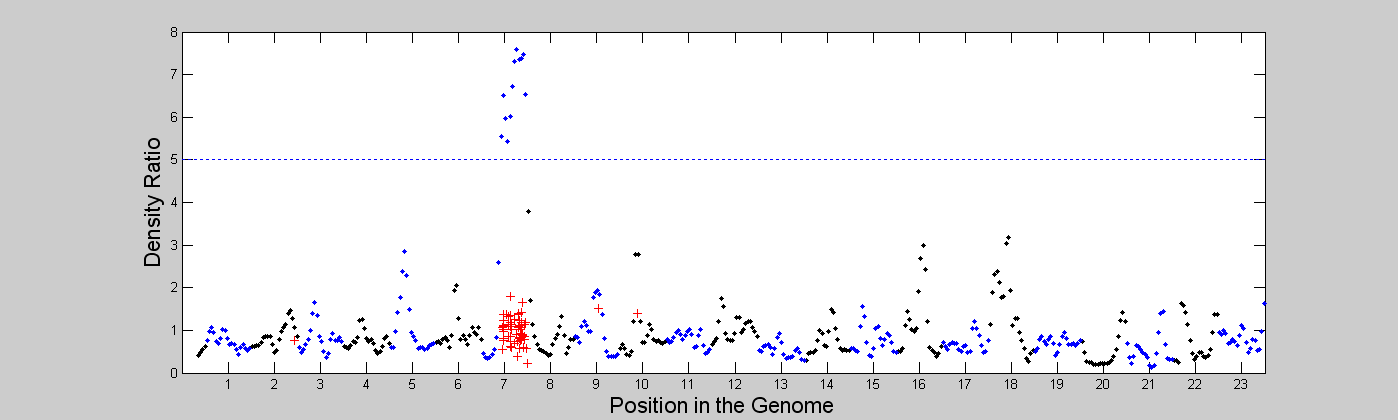

Supplement: Figure S2 — The local enrichment ratios (LER) for chromosomal enrichment of the latent factors. (0.86 MB ZIP) [file pcbi.1000920.s002.zip › fac27.png]

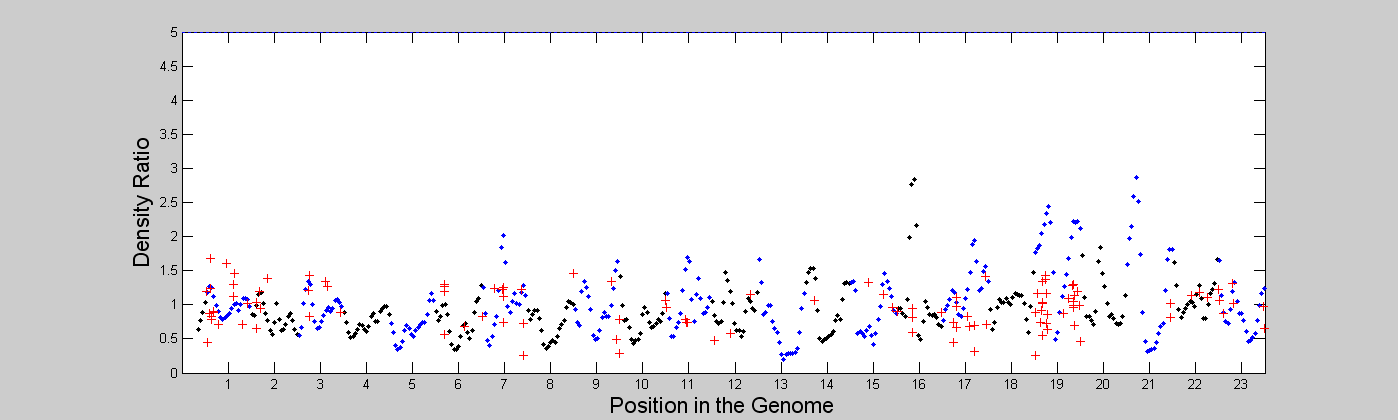

Supplement: Figure S2 — The local enrichment ratios (LER) for chromosomal enrichment of the latent factors. (0.86 MB ZIP) [file pcbi.1000920.s002.zip › fac28.png]

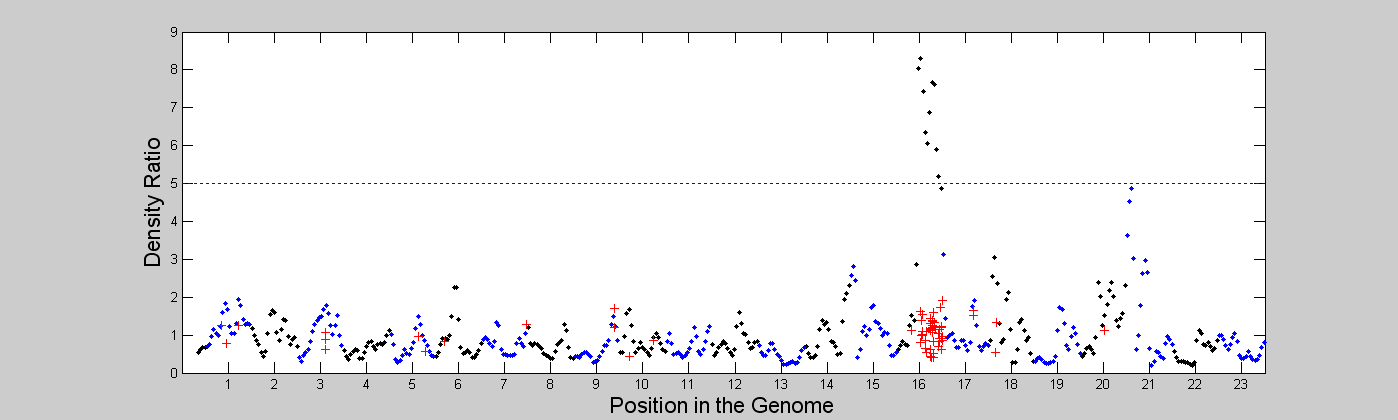

Supplement: Figure S2 — The local enrichment ratios (LER) for chromosomal enrichment of the latent factors. (0.86 MB ZIP) [file pcbi.1000920.s002.zip › fac29.png]

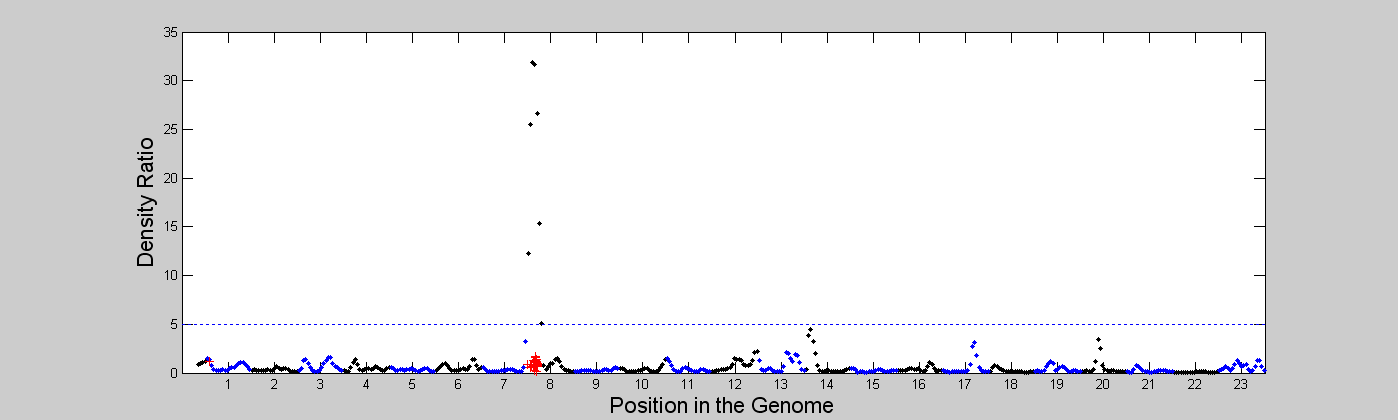

Supplement: Figure S2 — The local enrichment ratios (LER) for chromosomal enrichment of the latent factors. (0.86 MB ZIP) [file pcbi.1000920.s002.zip › fac30.png]

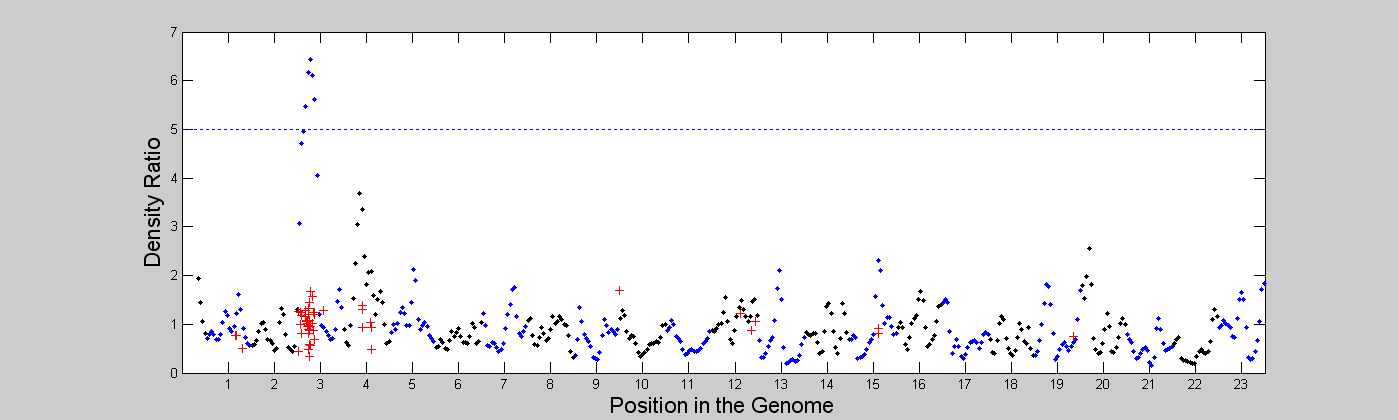

Supplement: Figure S2 — The local enrichment ratios (LER) for chromosomal enrichment of the latent factors. (0.86 MB ZIP) [file pcbi.1000920.s002.zip › fac31.png]

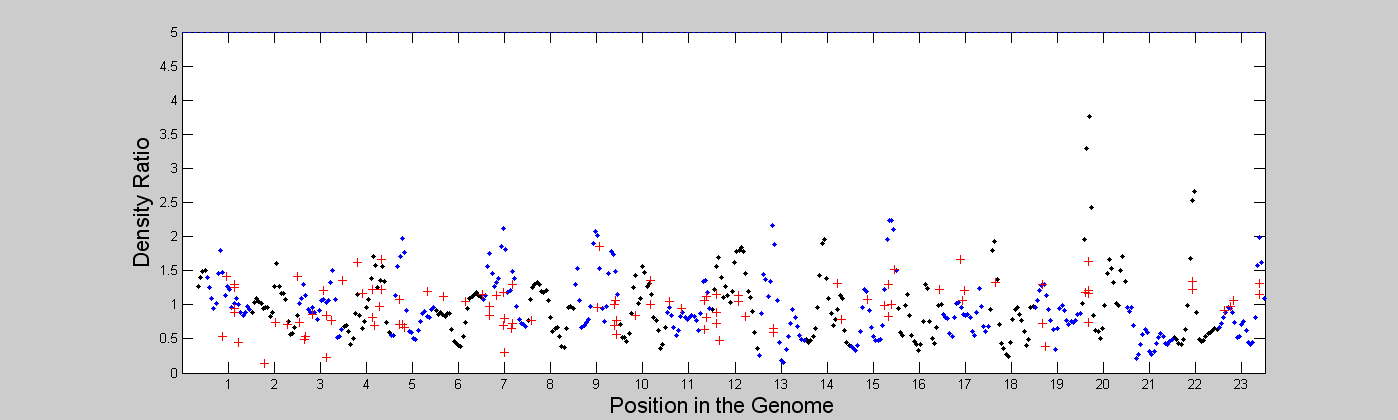

Supplement: Figure S2 — The local enrichment ratios (LER) for chromosomal enrichment of the latent factors. (0.86 MB ZIP) [file pcbi.1000920.s002.zip › fac32.png]

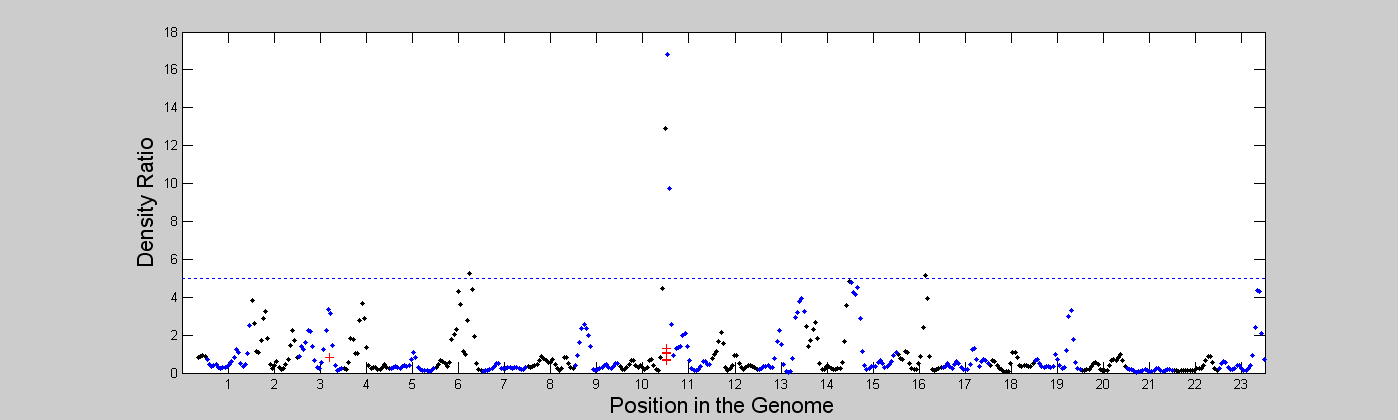

Supplement: Figure S2 — The local enrichment ratios (LER) for chromosomal enrichment of the latent factors. (0.86 MB ZIP) [file pcbi.1000920.s002.zip › fac33.png]

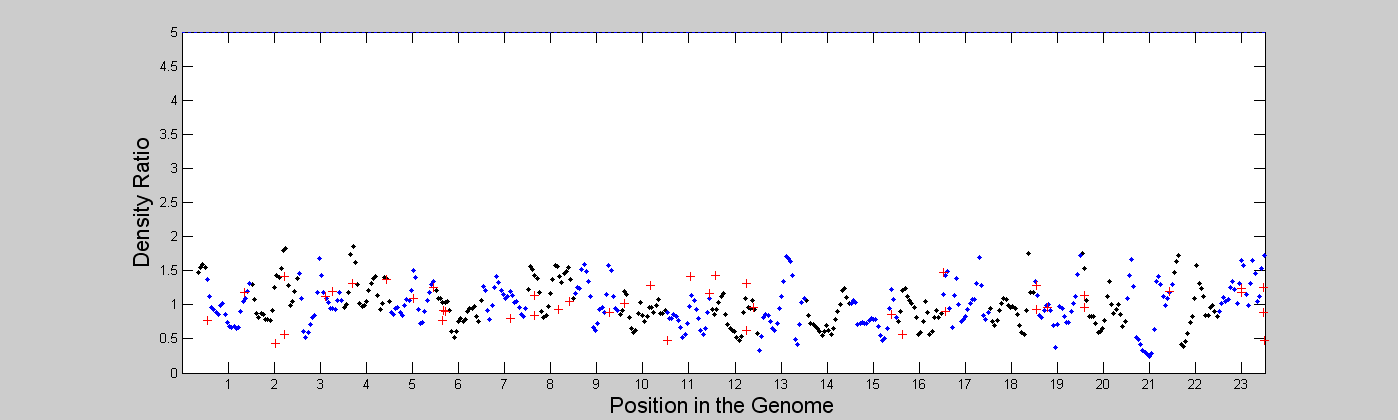

Supplement: Figure S2 — The local enrichment ratios (LER) for chromosomal enrichment of the latent factors. (0.86 MB ZIP) [file pcbi.1000920.s002.zip › fac34.png]

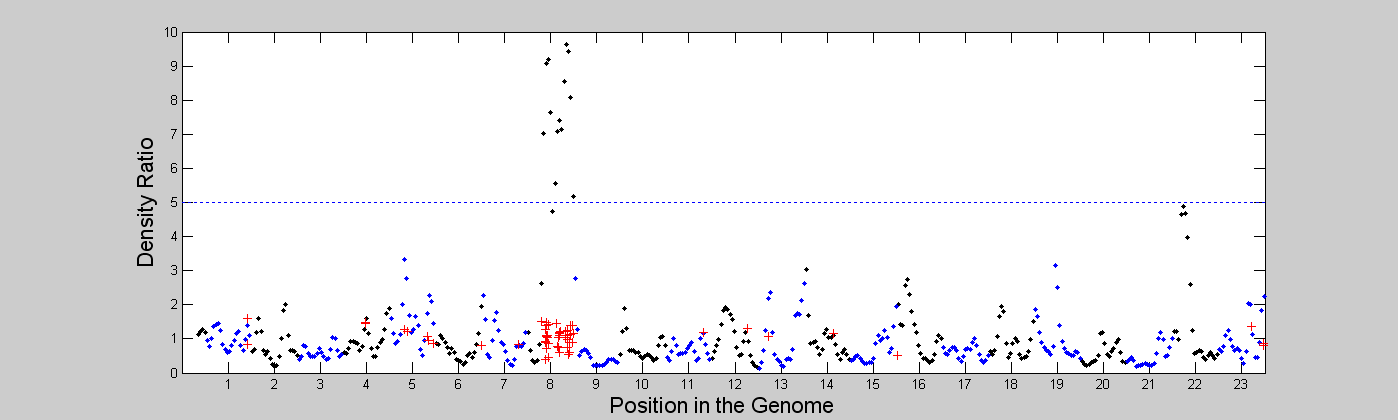

Supplement: Figure S2 — The local enrichment ratios (LER) for chromosomal enrichment of the latent factors. (0.86 MB ZIP) [file pcbi.1000920.s002.zip › fac35.png]

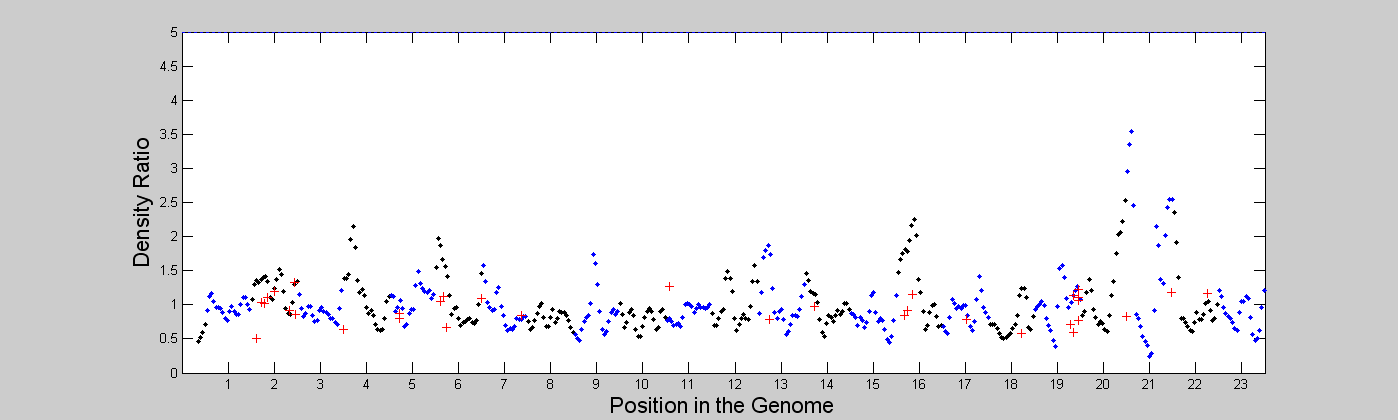

Supplement: Figure S2 — The local enrichment ratios (LER) for chromosomal enrichment of the latent factors. (0.86 MB ZIP) [file pcbi.1000920.s002.zip › fac36.png]

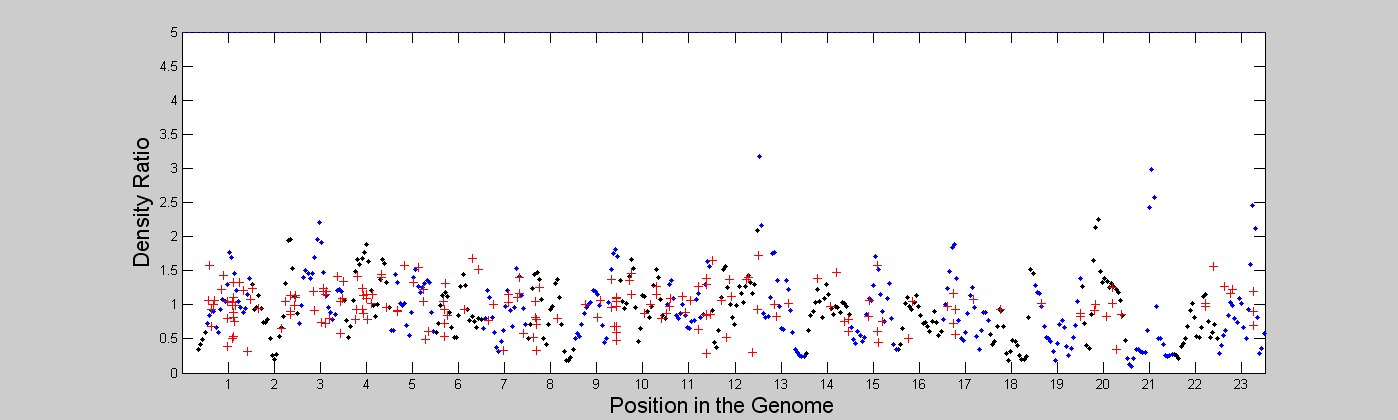

Supplement: Figure S2 — The local enrichment ratios (LER) for chromosomal enrichment of the latent factors. (0.86 MB ZIP) [file pcbi.1000920.s002.zip › fac37.png]

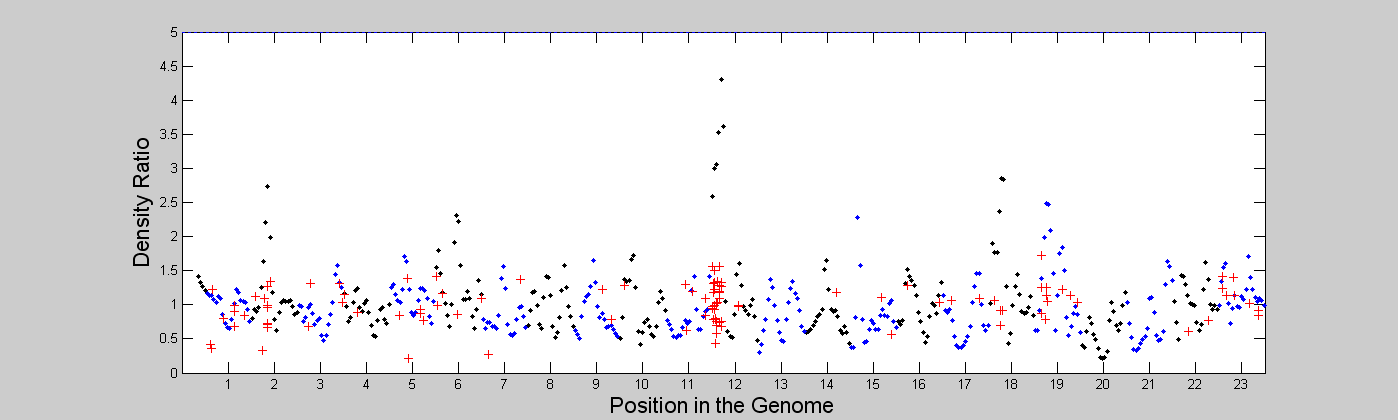

Supplement: Figure S2 — The local enrichment ratios (LER) for chromosomal enrichment of the latent factors. (0.86 MB ZIP) [file pcbi.1000920.s002.zip › fac38.png]

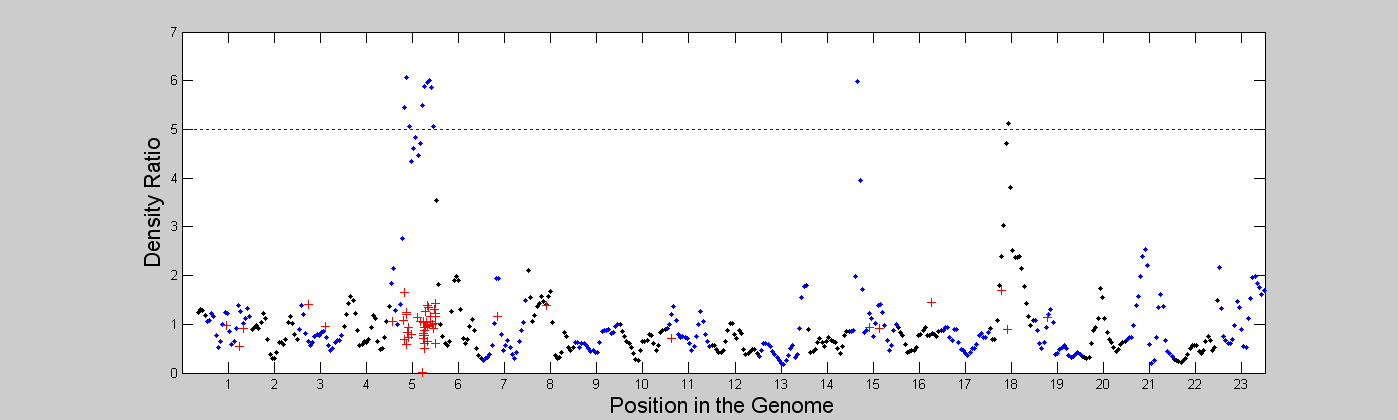

Supplement: Figure S2 — The local enrichment ratios (LER) for chromosomal enrichment of the latent factors. (0.86 MB ZIP) [file pcbi.1000920.s002.zip › fac39.png]

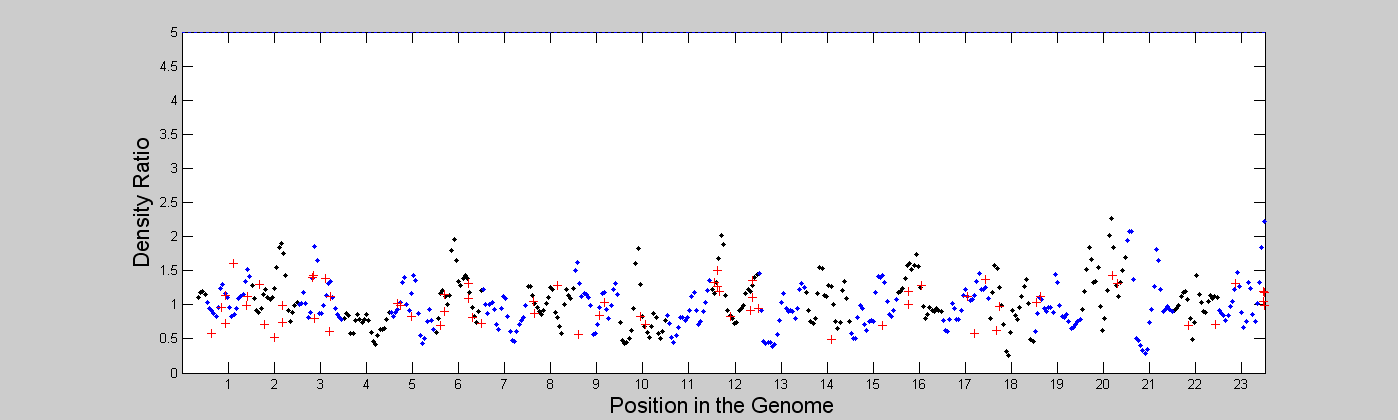

Supplement: Figure S2 — The local enrichment ratios (LER) for chromosomal enrichment of the latent factors. (0.86 MB ZIP) [file pcbi.1000920.s002.zip › fac40.png]

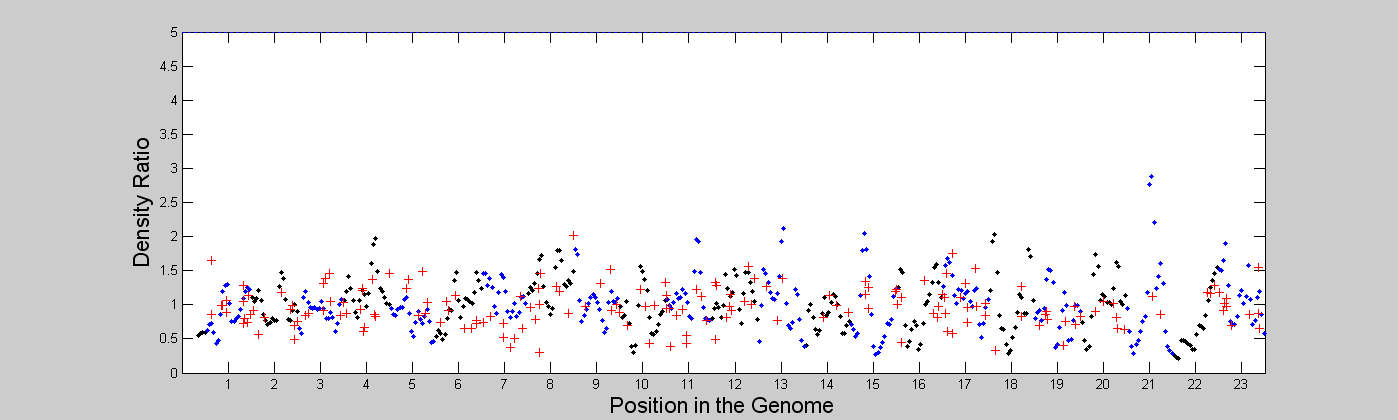

Supplement: Figure S2 — The local enrichment ratios (LER) for chromosomal enrichment of the latent factors. (0.86 MB ZIP) [file pcbi.1000920.s002.zip › fac41.png]

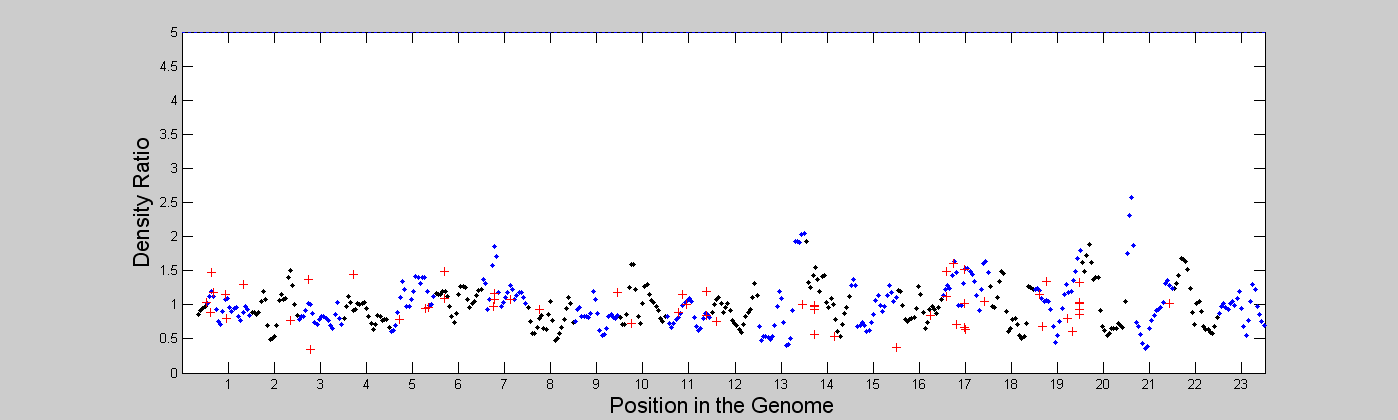

Supplement: Figure S2 — The local enrichment ratios (LER) for chromosomal enrichment of the latent factors. (0.86 MB ZIP) [file pcbi.1000920.s002.zip › fac42.png]

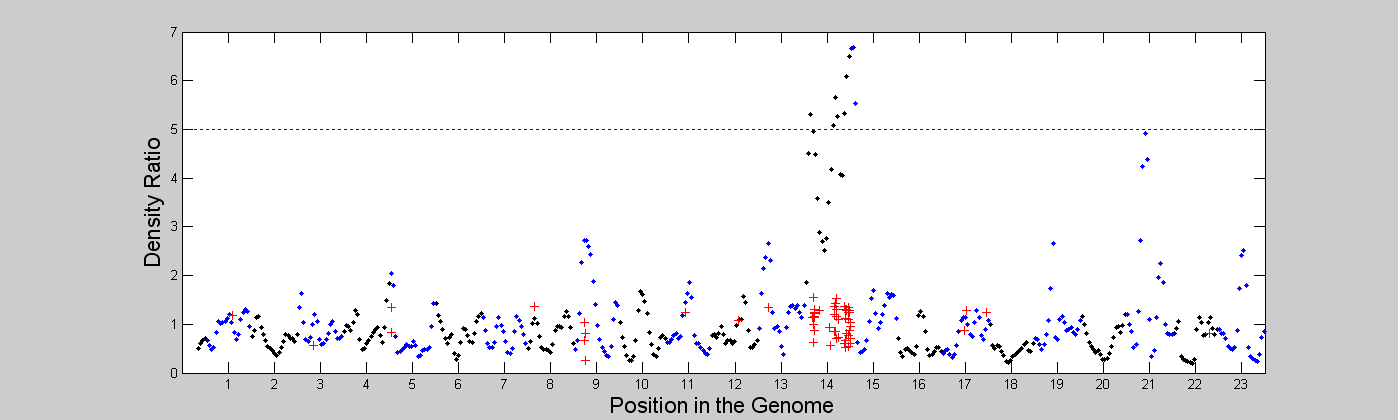

Supplement: Figure S2 — The local enrichment ratios (LER) for chromosomal enrichment of the latent factors. (0.86 MB ZIP) [file pcbi.1000920.s002.zip › fac43.png]

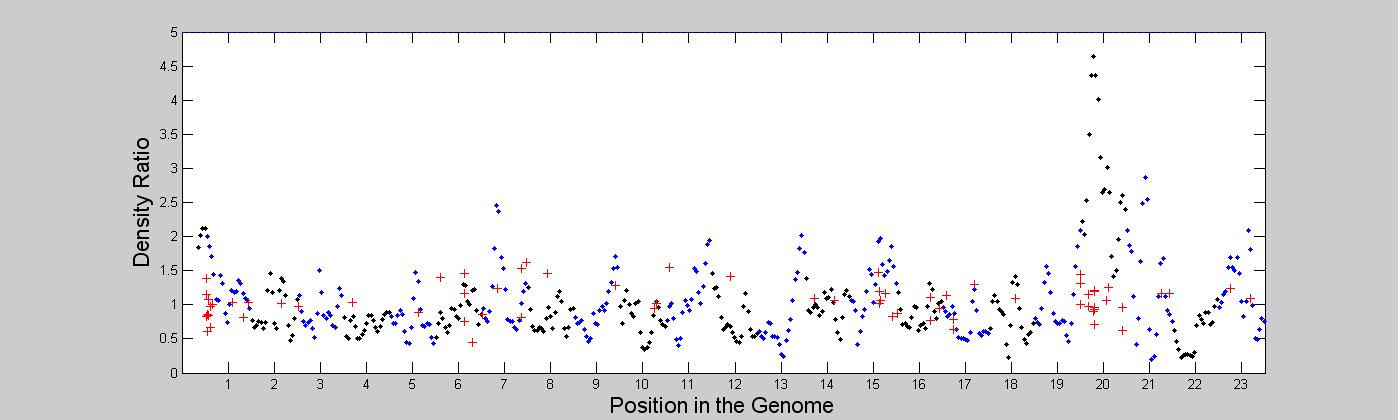

Supplement: Figure S2 — The local enrichment ratios (LER) for chromosomal enrichment of the latent factors. (0.86 MB ZIP) [file pcbi.1000920.s002.zip › fac44.png]

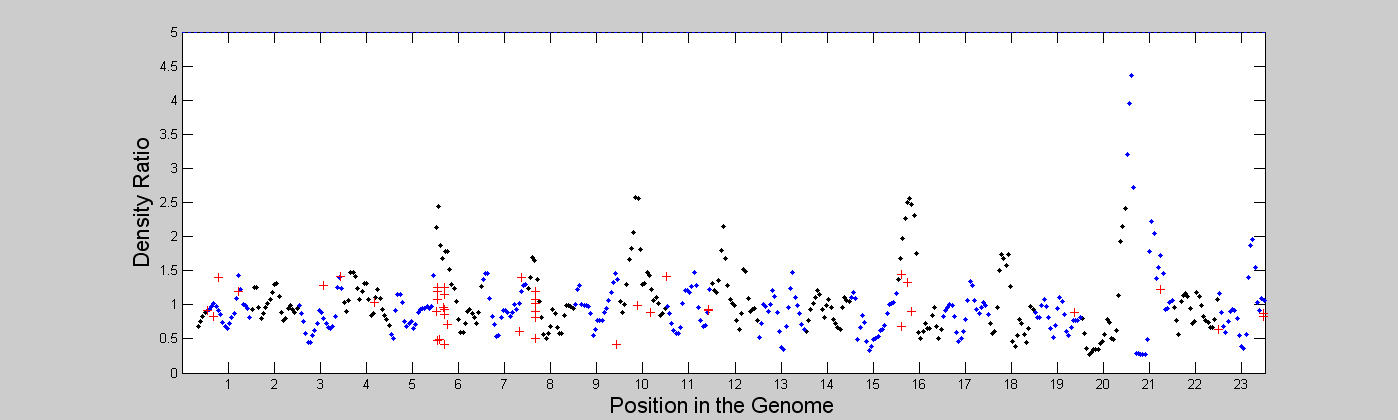

Supplement: Figure S2 — The local enrichment ratios (LER) for chromosomal enrichment of the latent factors. (0.86 MB ZIP) [file pcbi.1000920.s002.zip › fac45.png]

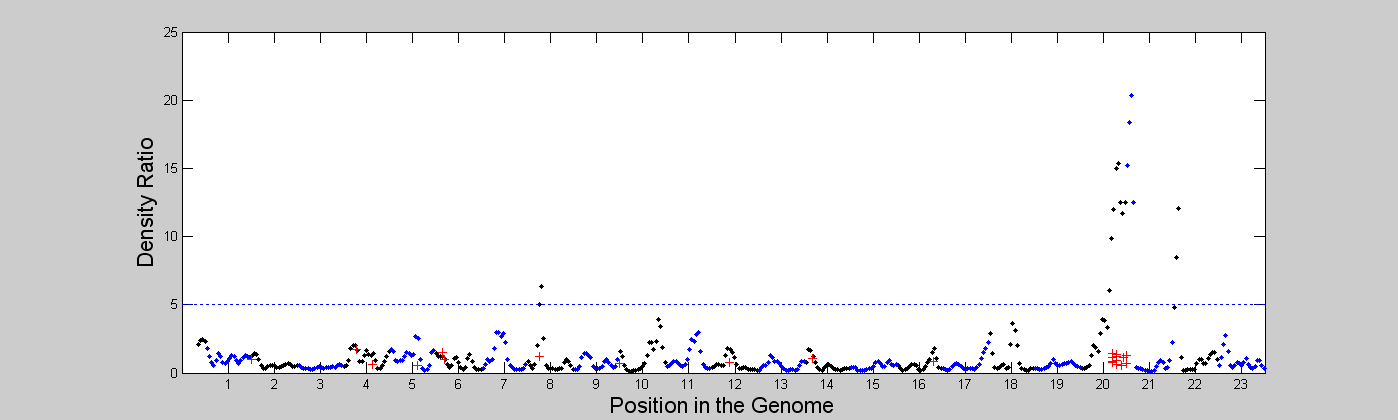

Supplement: Figure S2 — The local enrichment ratios (LER) for chromosomal enrichment of the latent factors. (0.86 MB ZIP) [file pcbi.1000920.s002.zip › fac46.png]

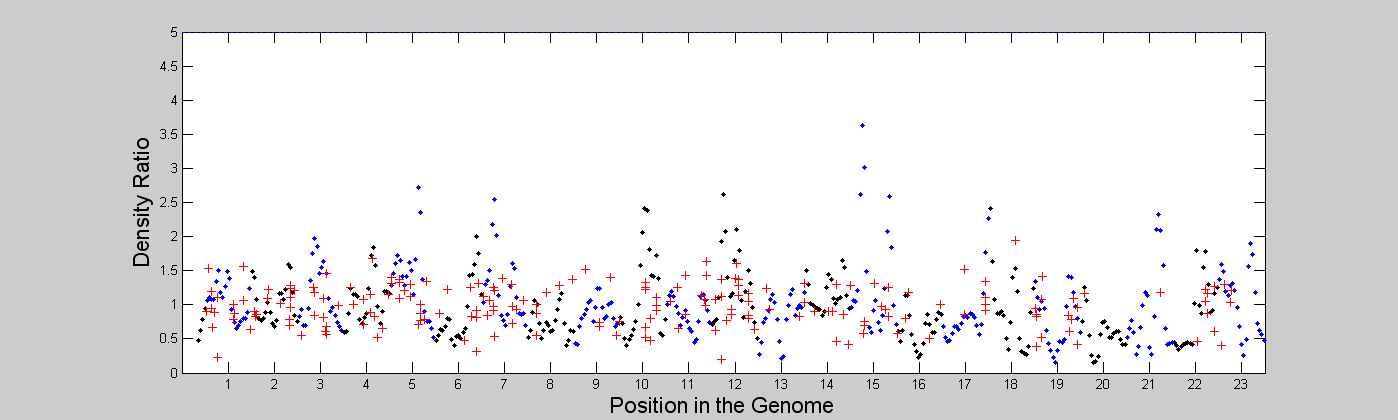

Supplement: Figure S2 — The local enrichment ratios (LER) for chromosomal enrichment of the latent factors. (0.86 MB ZIP) [file pcbi.1000920.s002.zip › fac47.png]

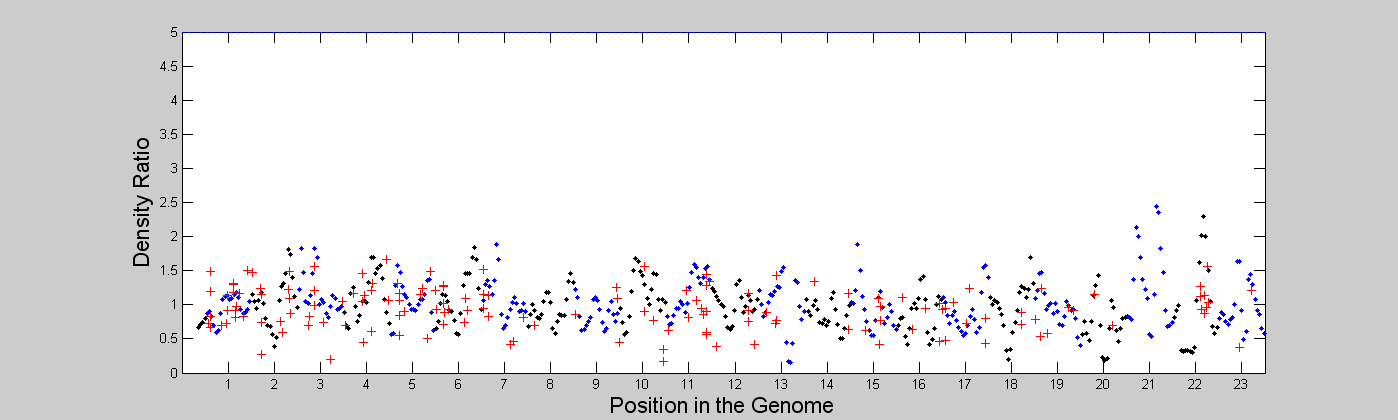

Supplement: Figure S2 — The local enrichment ratios (LER) for chromosomal enrichment of the latent factors. (0.86 MB ZIP) [file pcbi.1000920.s002.zip › fac48.png]

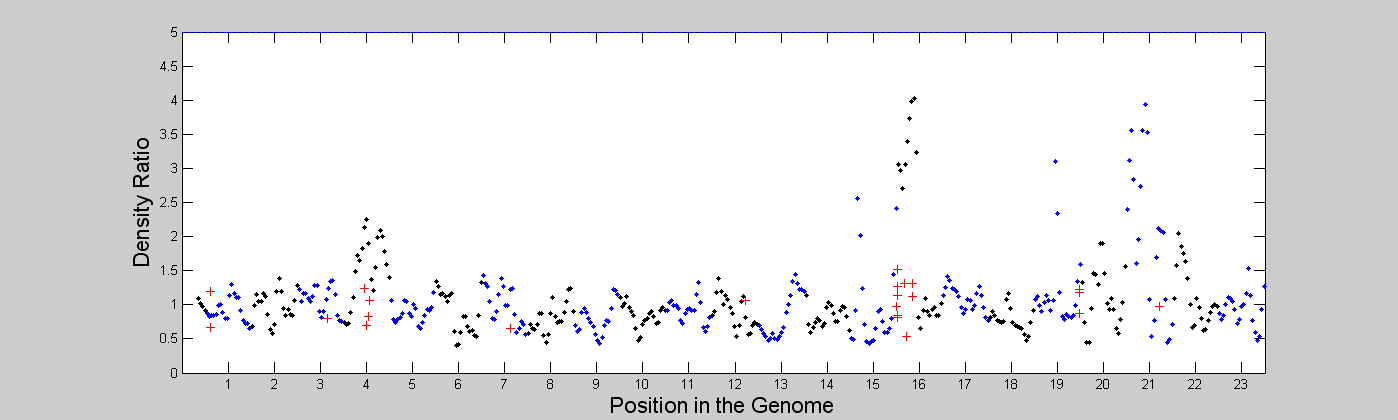

Supplement: Figure S2 — The local enrichment ratios (LER) for chromosomal enrichment of the latent factors. (0.86 MB ZIP) [file pcbi.1000920.s002.zip › fac49.png]

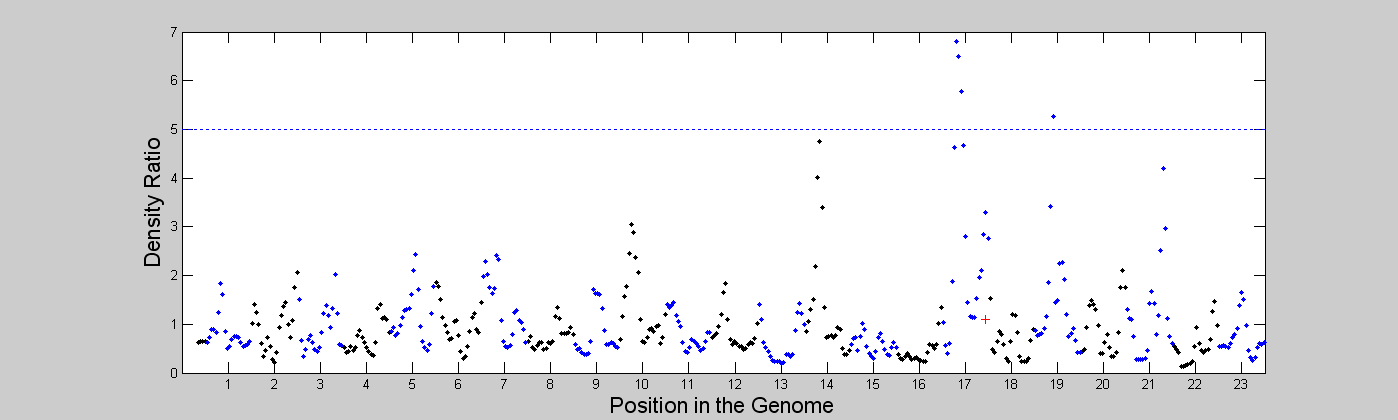

Supplement: Figure S2 — The local enrichment ratios (LER) for chromosomal enrichment of the latent factors. (0.86 MB ZIP) [file pcbi.1000920.s002.zip › fac50.png]

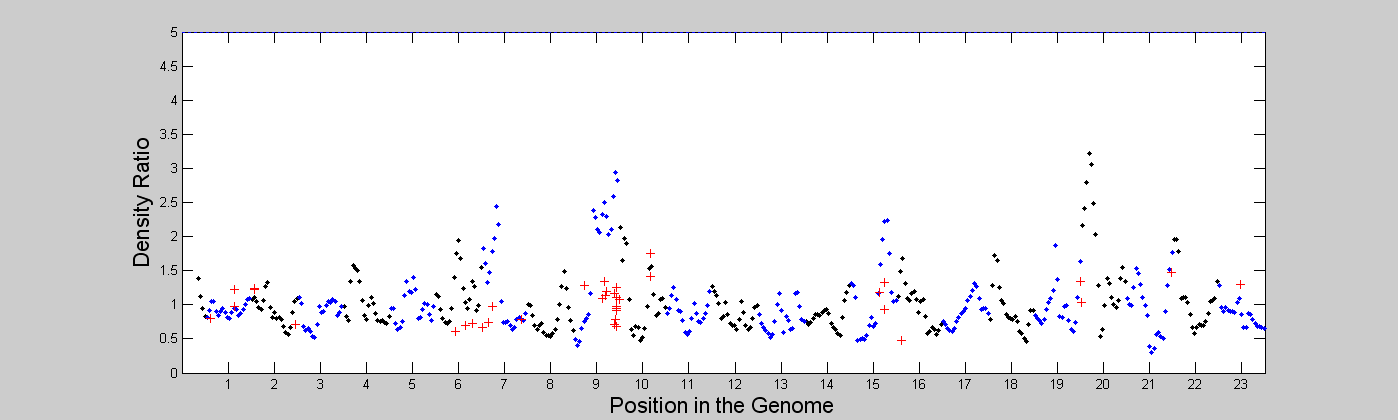

Supplement: Figure S2 — The local enrichment ratios (LER) for chromosomal enrichment of the latent factors. (0.86 MB ZIP) [file pcbi.1000920.s002.zip › fac51.png]

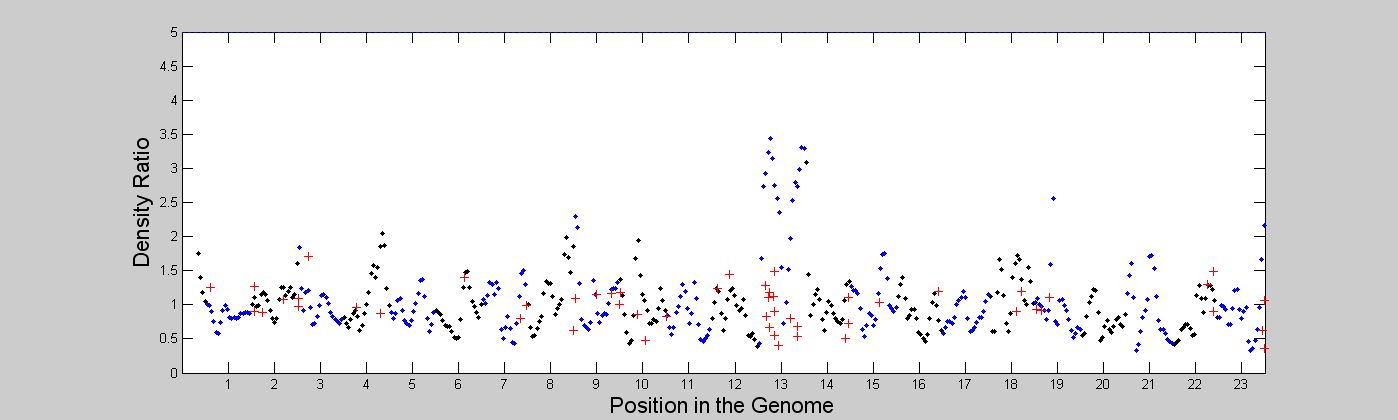

Supplement: Figure S2 — The local enrichment ratios (LER) for chromosomal enrichment of the latent factors. (0.86 MB ZIP) [file pcbi.1000920.s002.zip › fac52.png]
