# Supplementary figures and images for: Latent Factor Analysis to Discover Pathway-Associated Putative Segmental Aneuploidies in Human Cancers (part 2 of 2)
Source: PLoS Comput Biol. 2010 Sep 2;6(9):e1000920. doi: 10.1371/journal.pcbi.1000920 (PMC2932681; doi:10.1371/journal.pcbi.1000920)

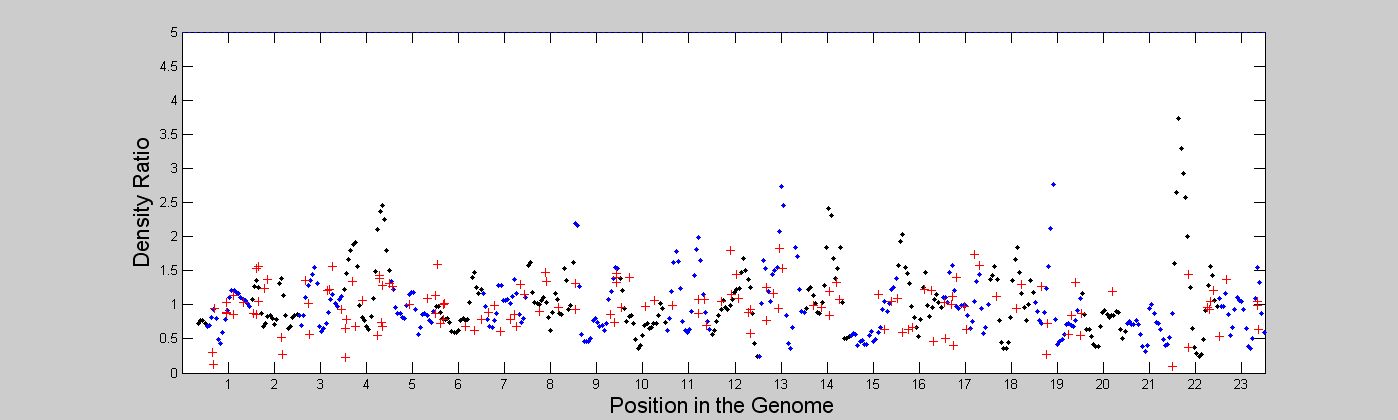

Supplement: Figure S2 — The local enrichment ratios (LER) for chromosomal enrichment of the latent factors. (0.86 MB ZIP) [file pcbi.1000920.s002.zip › fac53.png]

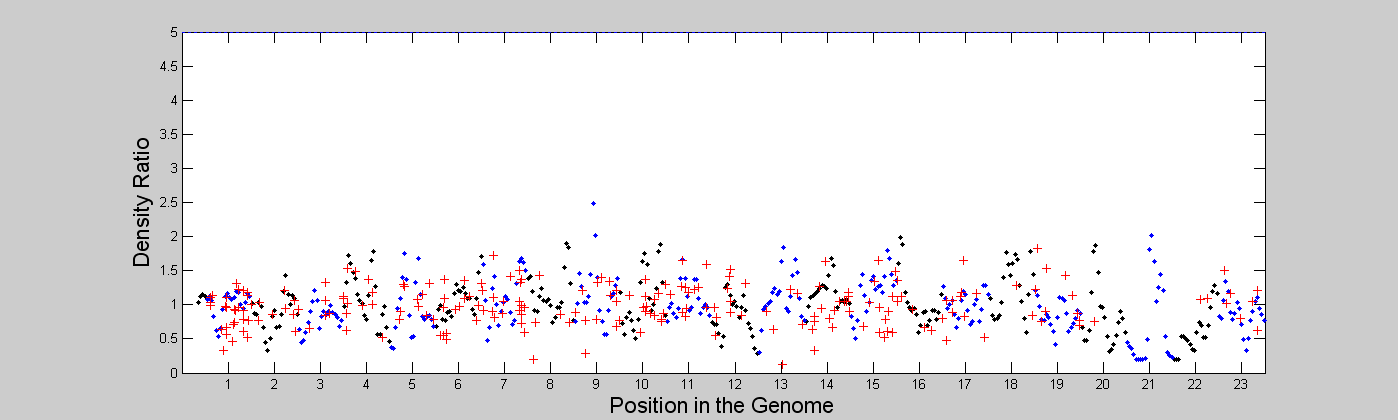

Supplement: Figure S2 — The local enrichment ratios (LER) for chromosomal enrichment of the latent factors. (0.86 MB ZIP) [file pcbi.1000920.s002.zip › fac54.png]

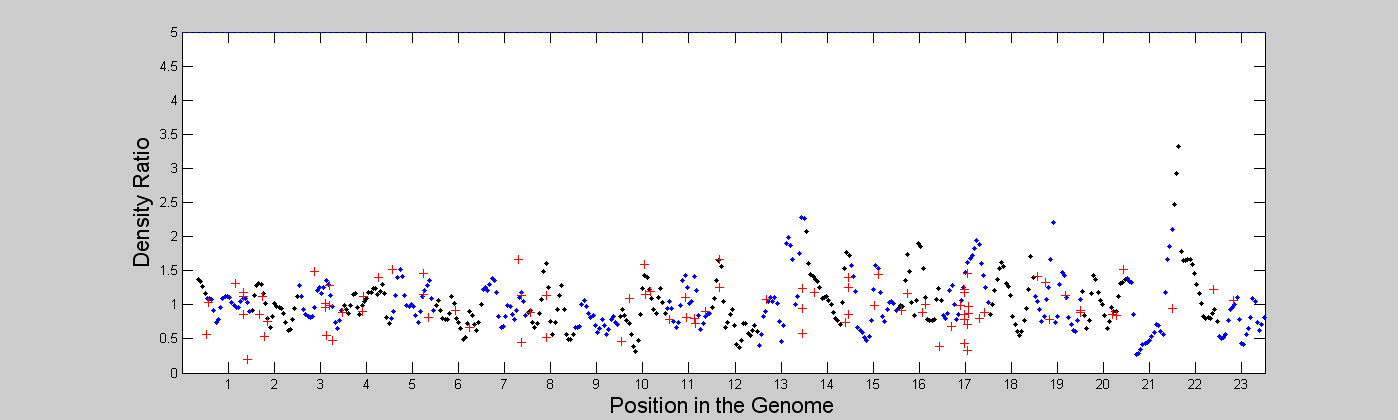

Supplement: Figure S2 — The local enrichment ratios (LER) for chromosomal enrichment of the latent factors. (0.86 MB ZIP) [file pcbi.1000920.s002.zip › fac55.png]

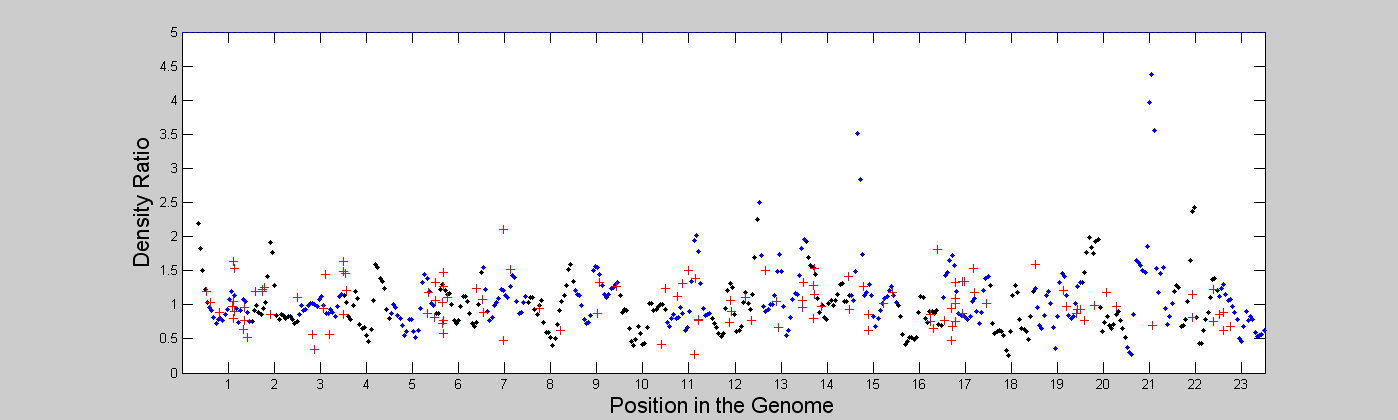

Supplement: Figure S2 — The local enrichment ratios (LER) for chromosomal enrichment of the latent factors. (0.86 MB ZIP) [file pcbi.1000920.s002.zip › fac56.png]

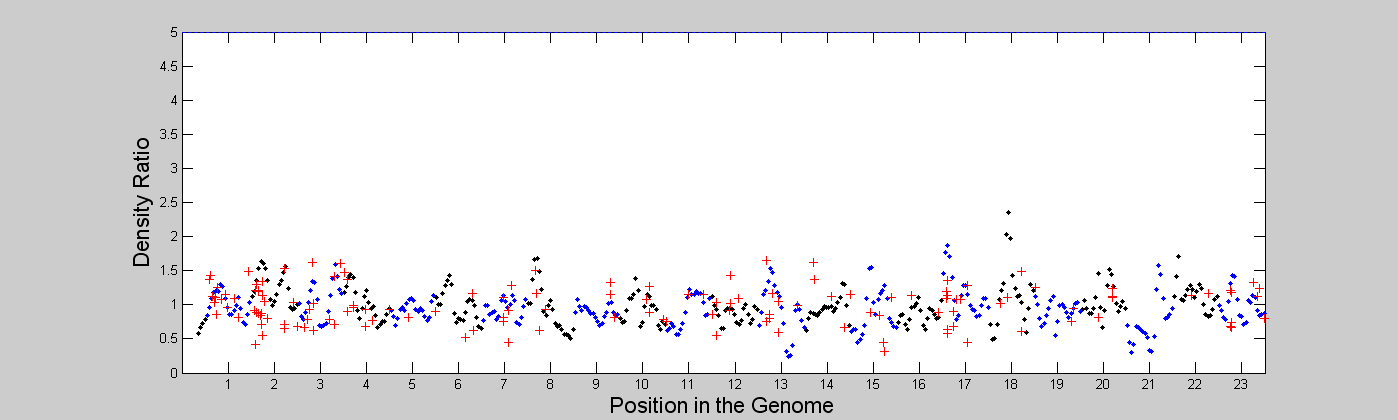

Supplement: Figure S2 — The local enrichment ratios (LER) for chromosomal enrichment of the latent factors. (0.86 MB ZIP) [file pcbi.1000920.s002.zip › fac1.png]

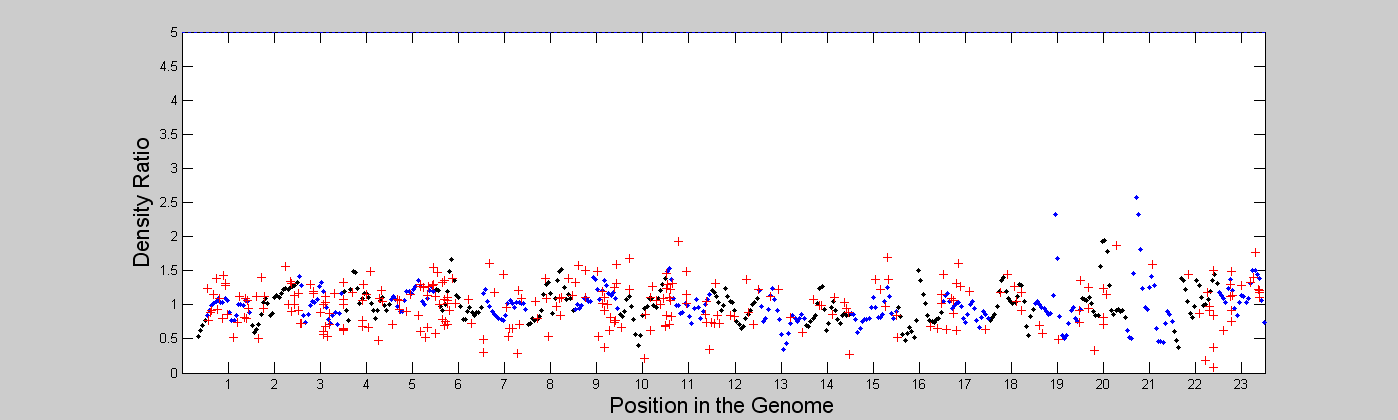

Supplement: Figure S2 — The local enrichment ratios (LER) for chromosomal enrichment of the latent factors. (0.86 MB ZIP) [file pcbi.1000920.s002.zip › fac2.png]

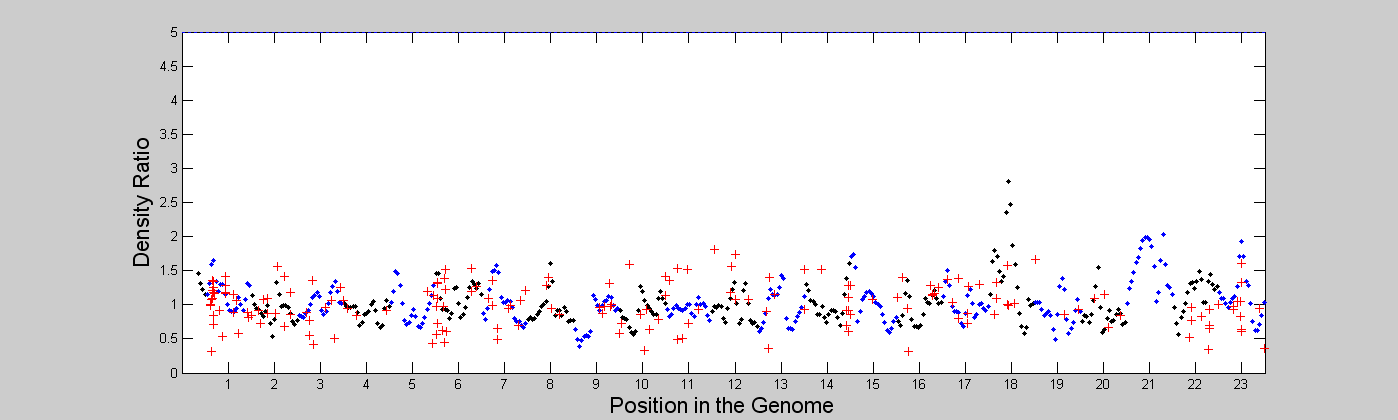

Supplement: Figure S2 — The local enrichment ratios (LER) for chromosomal enrichment of the latent factors. (0.86 MB ZIP) [file pcbi.1000920.s002.zip › fac3.png]

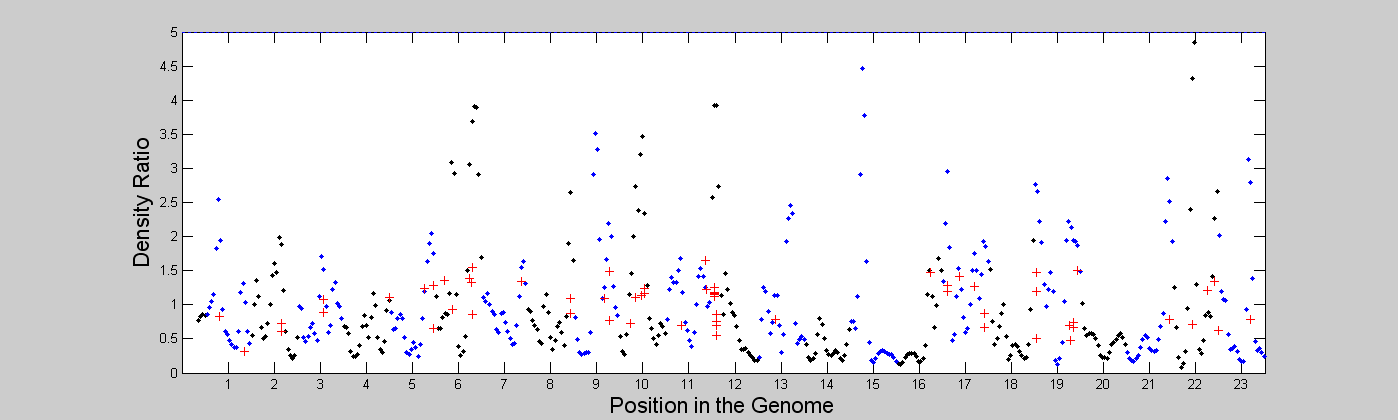

Supplement: Figure S2 — The local enrichment ratios (LER) for chromosomal enrichment of the latent factors. (0.86 MB ZIP) [file pcbi.1000920.s002.zip › fac4.png]

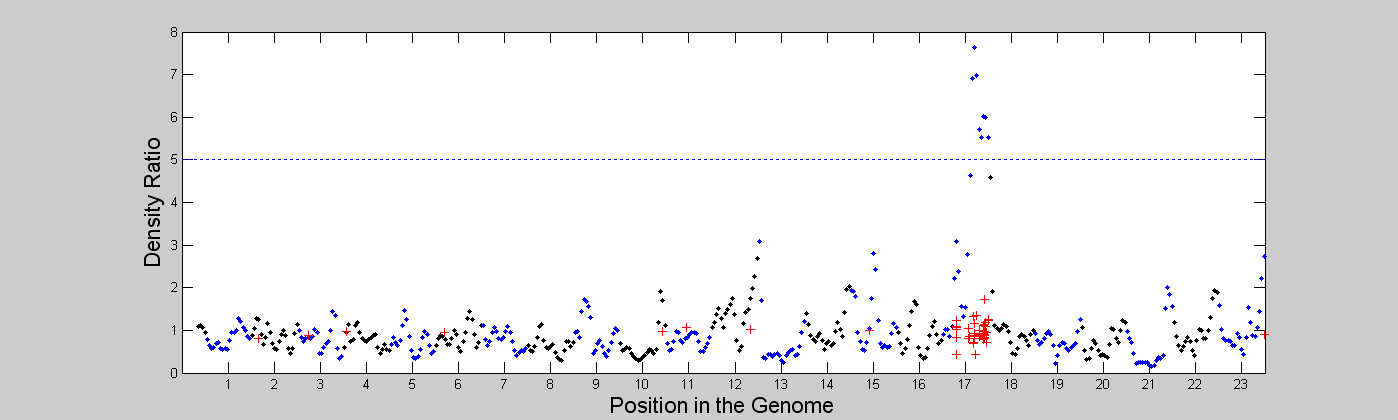

Supplement: Figure S2 — The local enrichment ratios (LER) for chromosomal enrichment of the latent factors. (0.86 MB ZIP) [file pcbi.1000920.s002.zip › fac5.png]

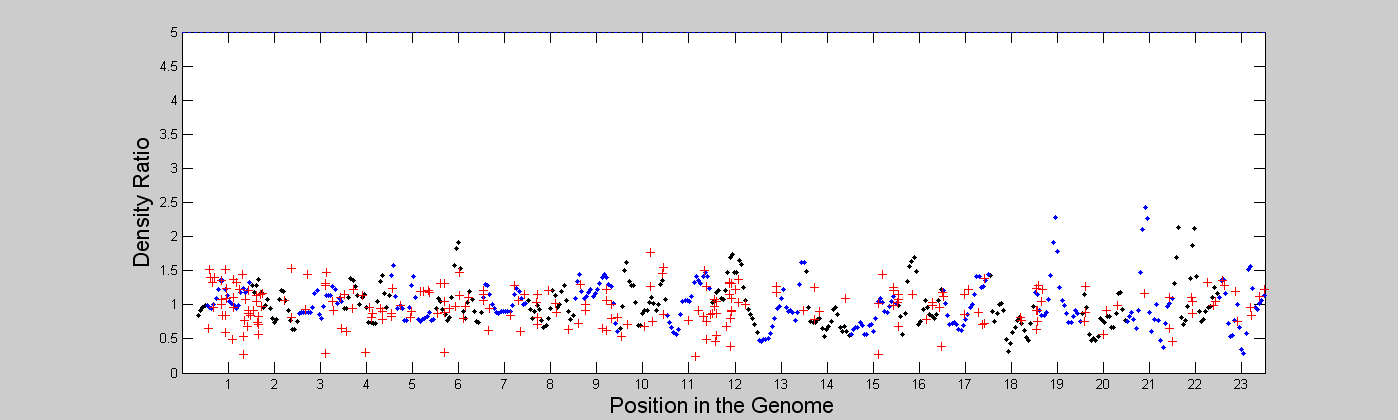

Supplement: Figure S2 — The local enrichment ratios (LER) for chromosomal enrichment of the latent factors. (0.86 MB ZIP) [file pcbi.1000920.s002.zip › fac6.png]

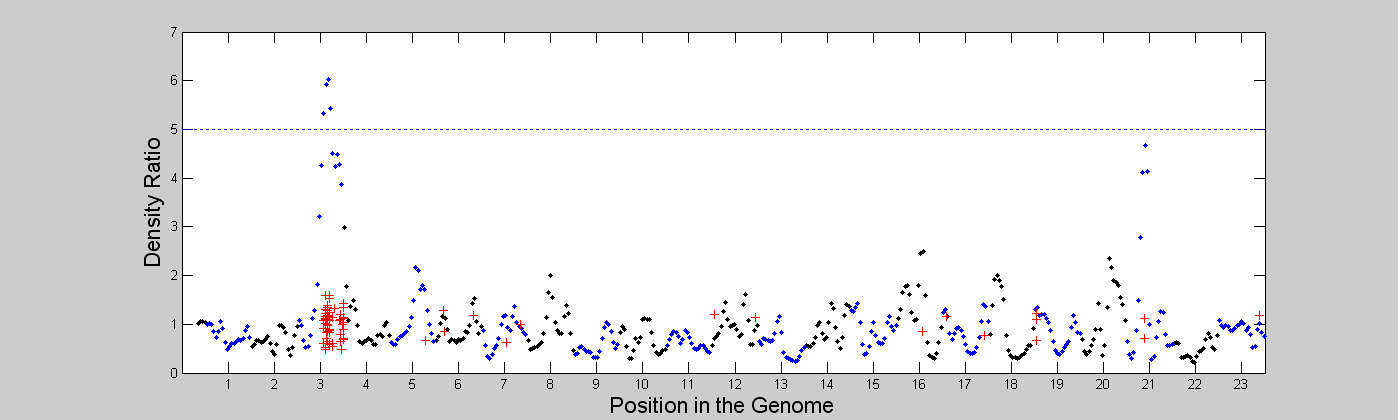

Supplement: Figure S2 — The local enrichment ratios (LER) for chromosomal enrichment of the latent factors. (0.86 MB ZIP) [file pcbi.1000920.s002.zip › fac7.png]

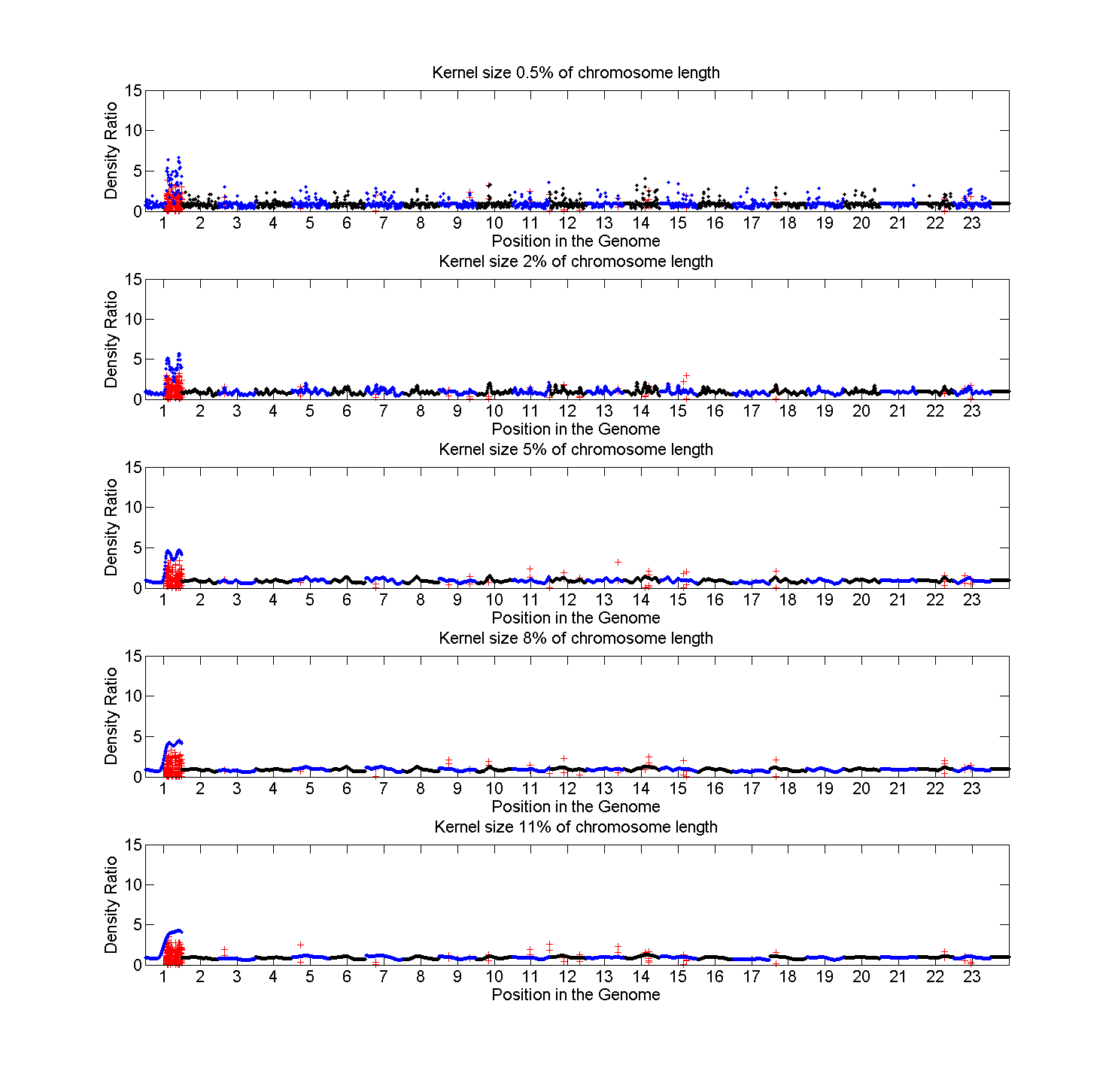

Supplement: Figure S3 — The local enrichment ratios (LER) for chromosomal enrichment of the latent factor 26 using different amount of Kernel widths. (0.25 MB TIF) [file pcbi.1000920.s003.tif]

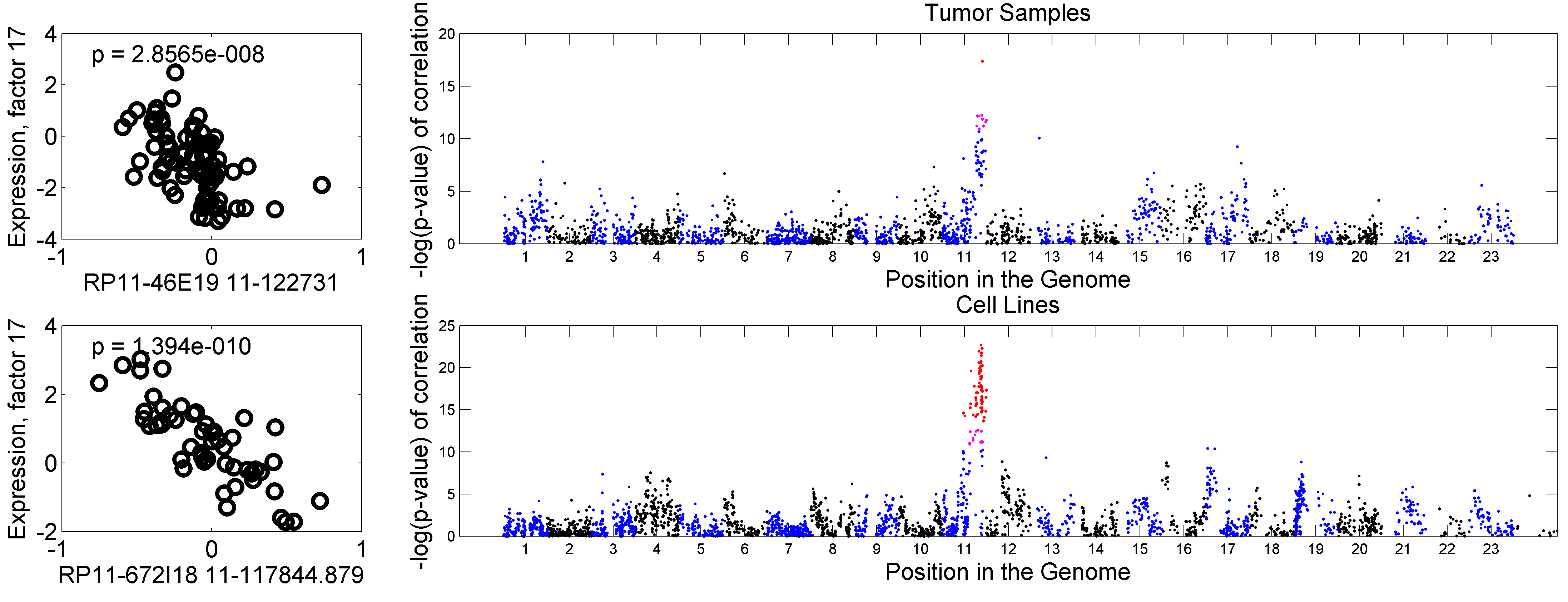

Supplement: Figure S4 — The association of the BAC clones with the expression of CNA-associated factors. (2.15 MB ZIP) [file pcbi.1000920.s004.zip › factor17_CNV.png]

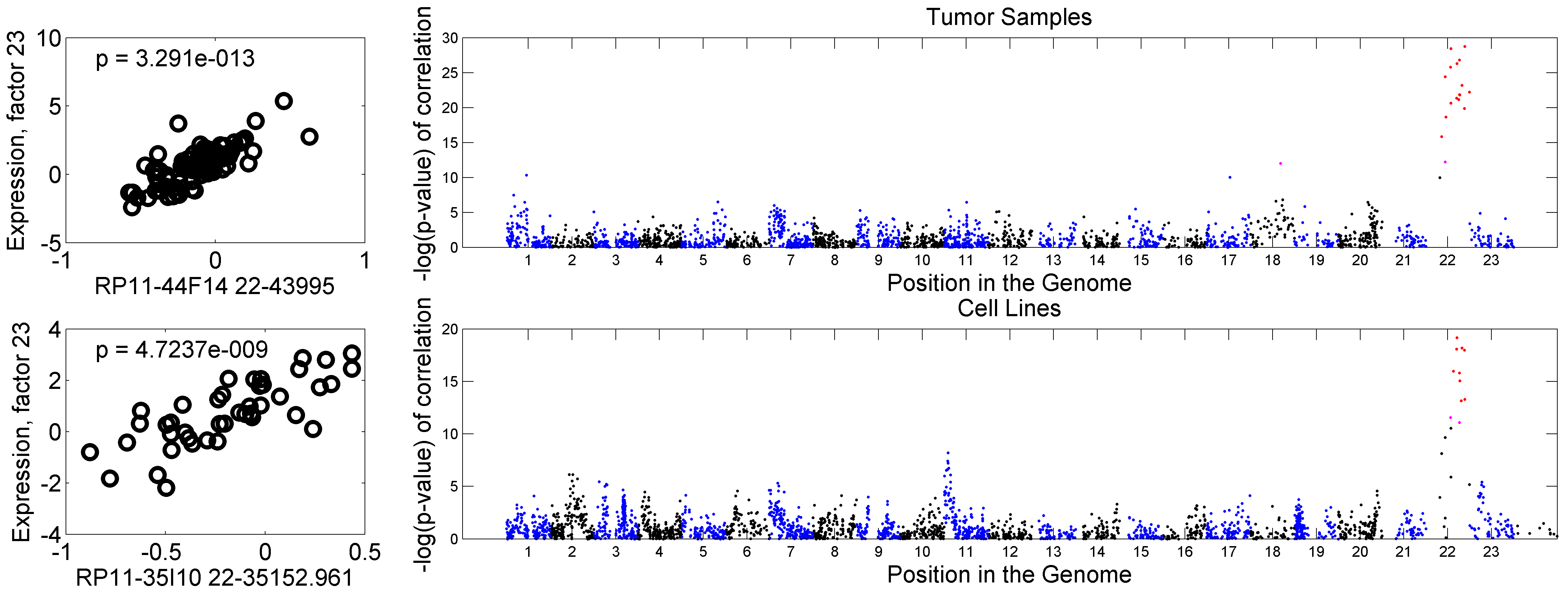

Supplement: Figure S4 — The association of the BAC clones with the expression of CNA-associated factors. (2.15 MB ZIP) [file pcbi.1000920.s004.zip › factor23_CNV.png]

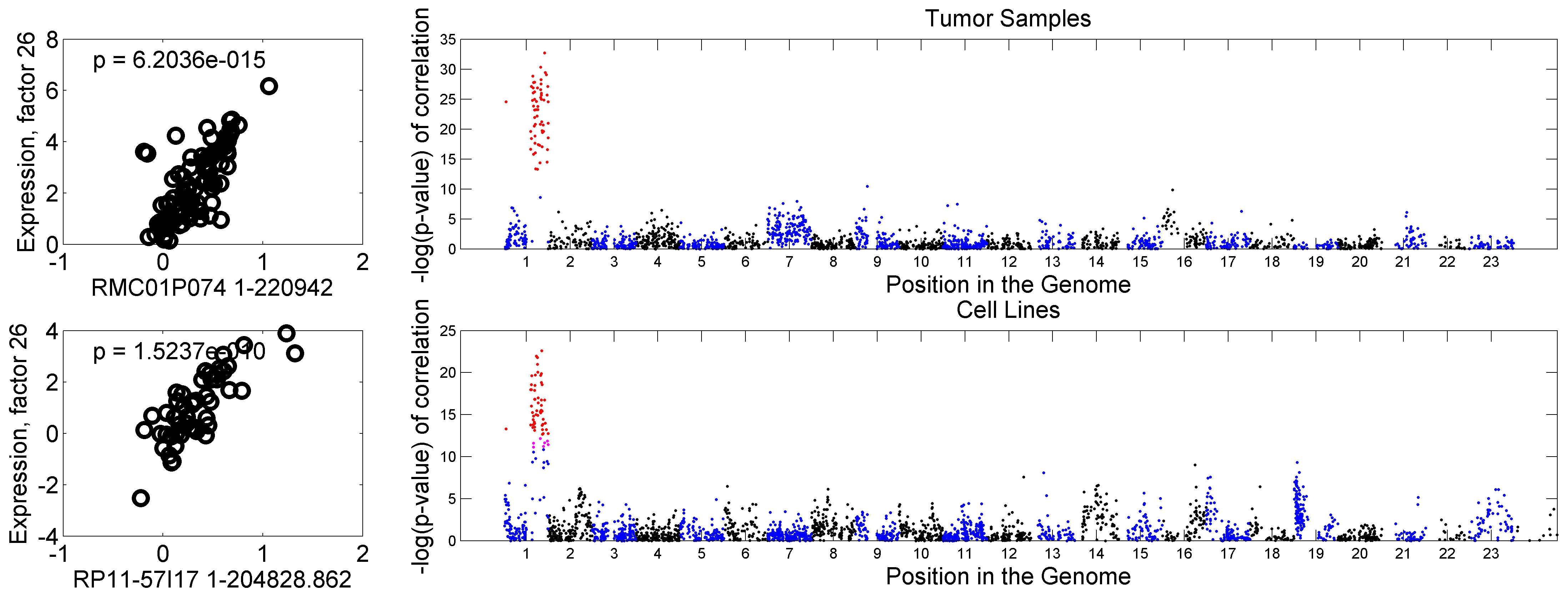

Supplement: Figure S4 — The association of the BAC clones with the expression of CNA-associated factors. (2.15 MB ZIP) [file pcbi.1000920.s004.zip › factor26_CNV.png]

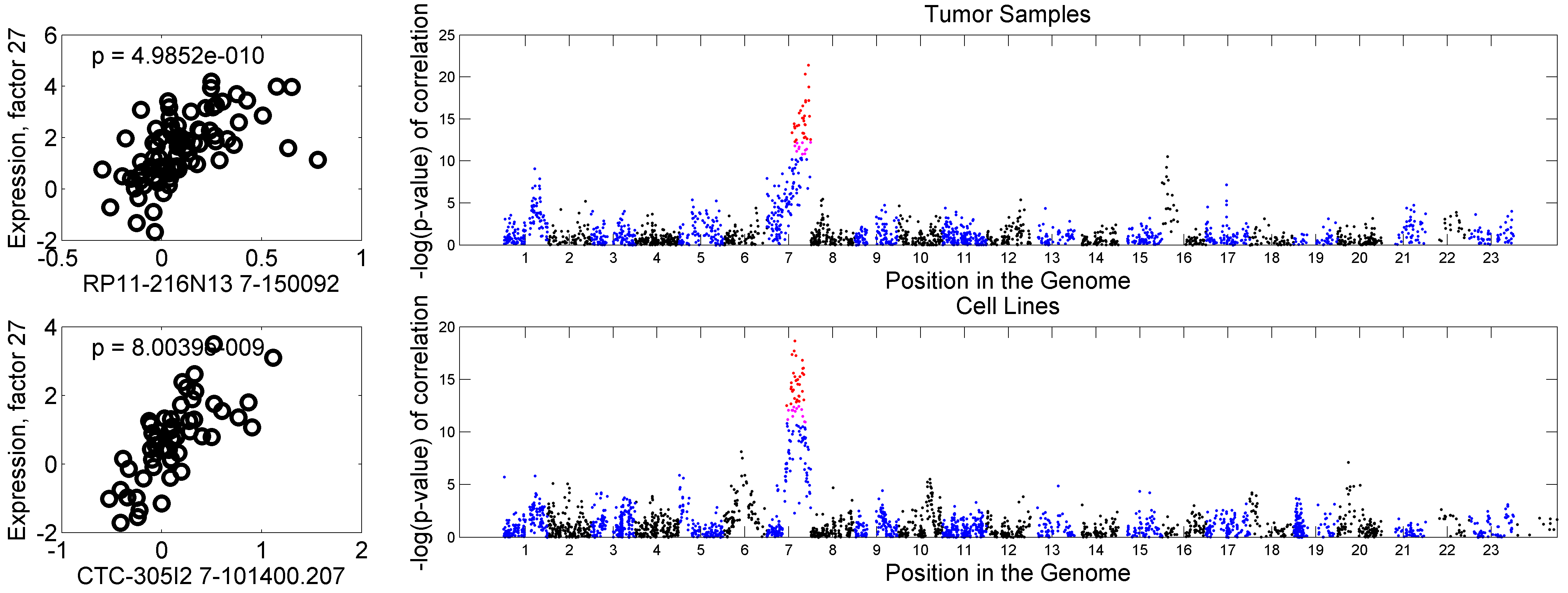

Supplement: Figure S4 — The association of the BAC clones with the expression of CNA-associated factors. (2.15 MB ZIP) [file pcbi.1000920.s004.zip › factor27_CNV.png]

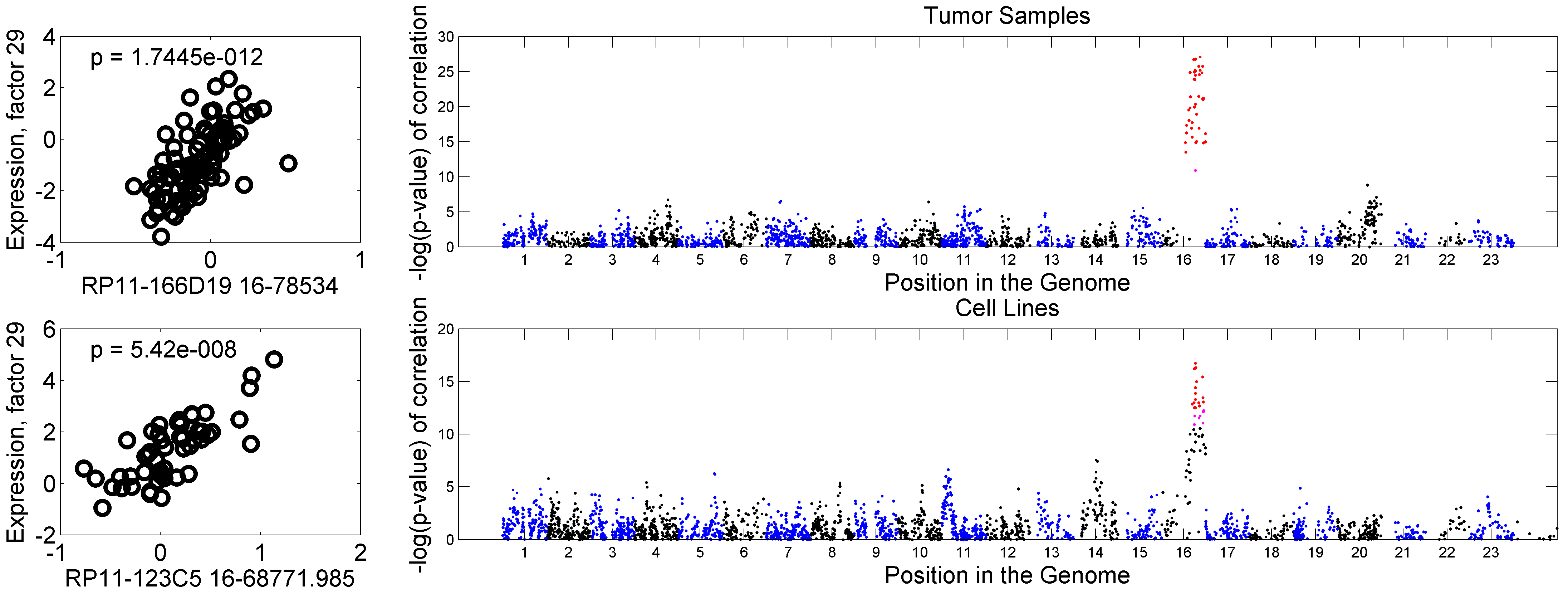

Supplement: Figure S4 — The association of the BAC clones with the expression of CNA-associated factors. (2.15 MB ZIP) [file pcbi.1000920.s004.zip › factor29_CNV.png]

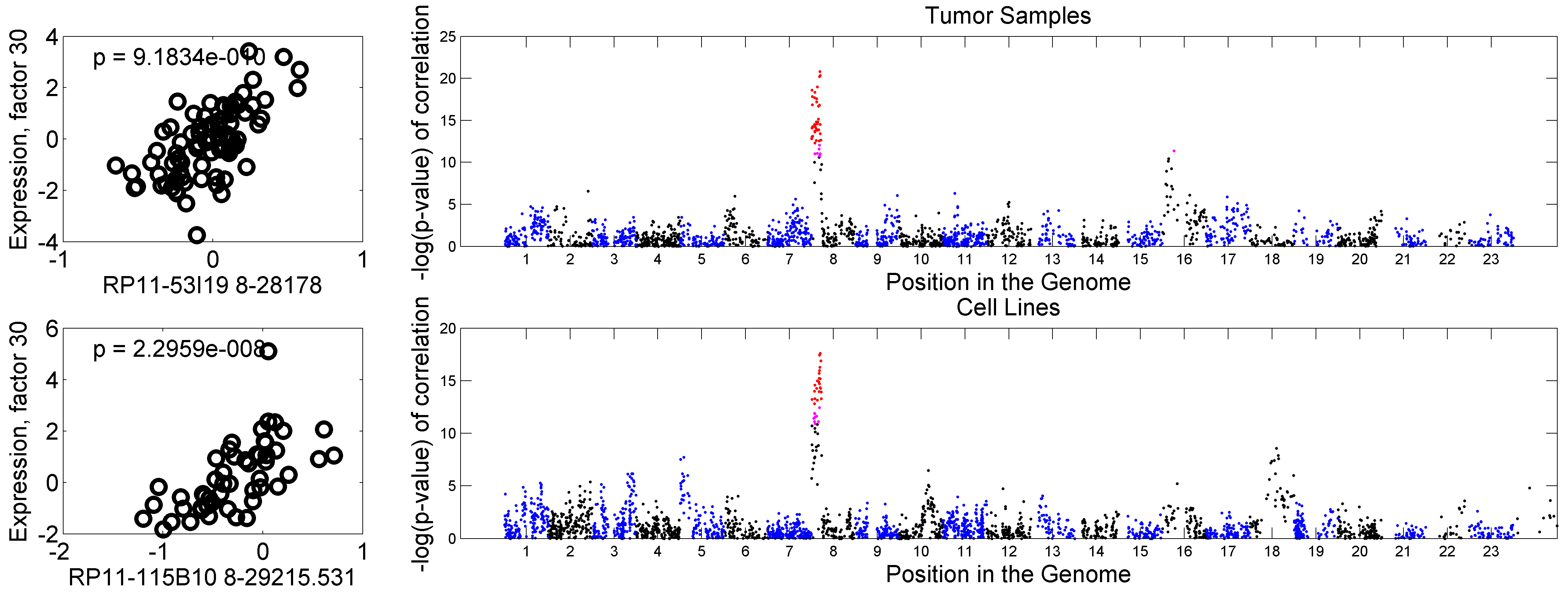

Supplement: Figure S4 — The association of the BAC clones with the expression of CNA-associated factors. (2.15 MB ZIP) [file pcbi.1000920.s004.zip › factor30_CNV.png]

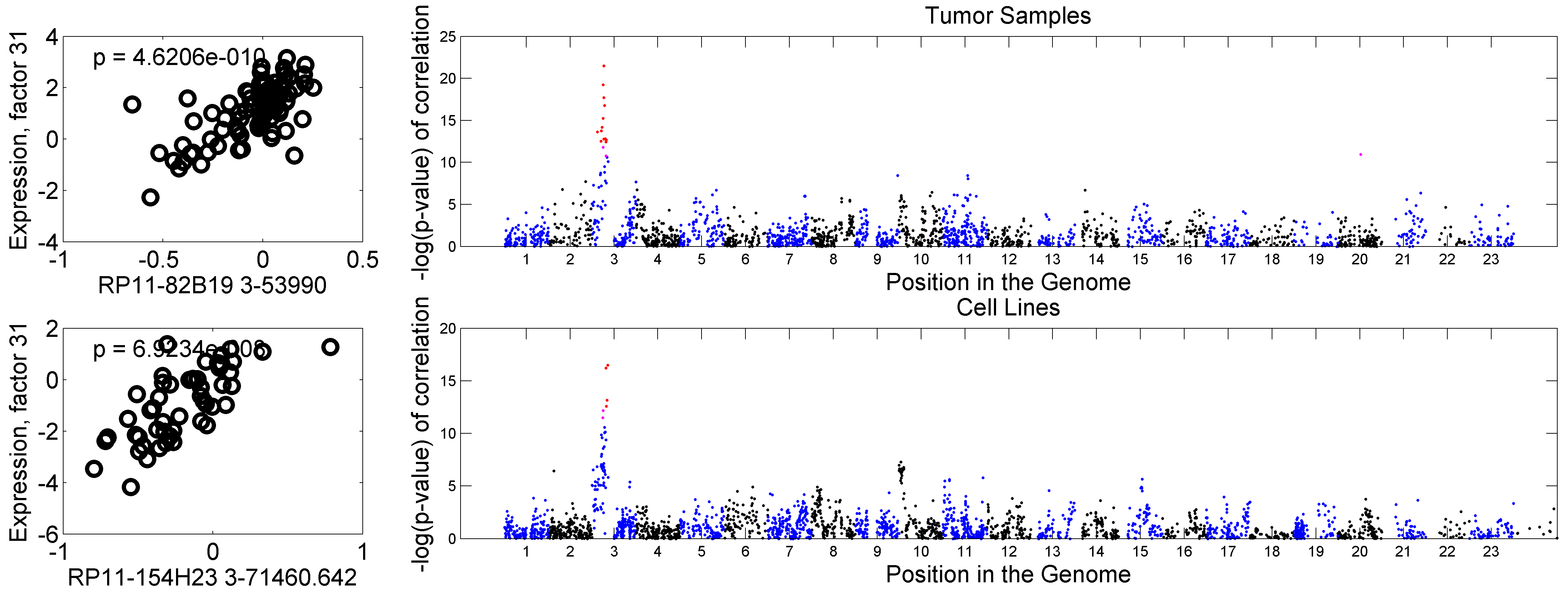

Supplement: Figure S4 — The association of the BAC clones with the expression of CNA-associated factors. (2.15 MB ZIP) [file pcbi.1000920.s004.zip › factor31_CNV.png]

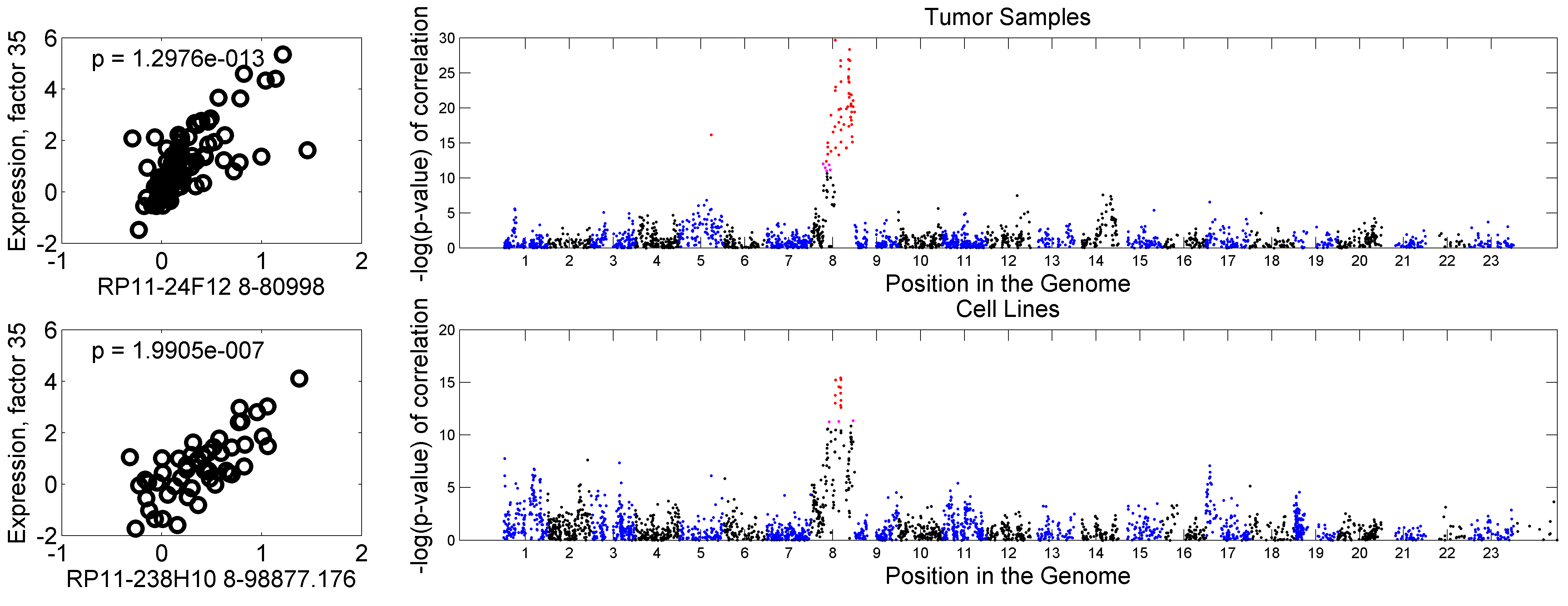

Supplement: Figure S4 — The association of the BAC clones with the expression of CNA-associated factors. (2.15 MB ZIP) [file pcbi.1000920.s004.zip › factor35_CNV.png]

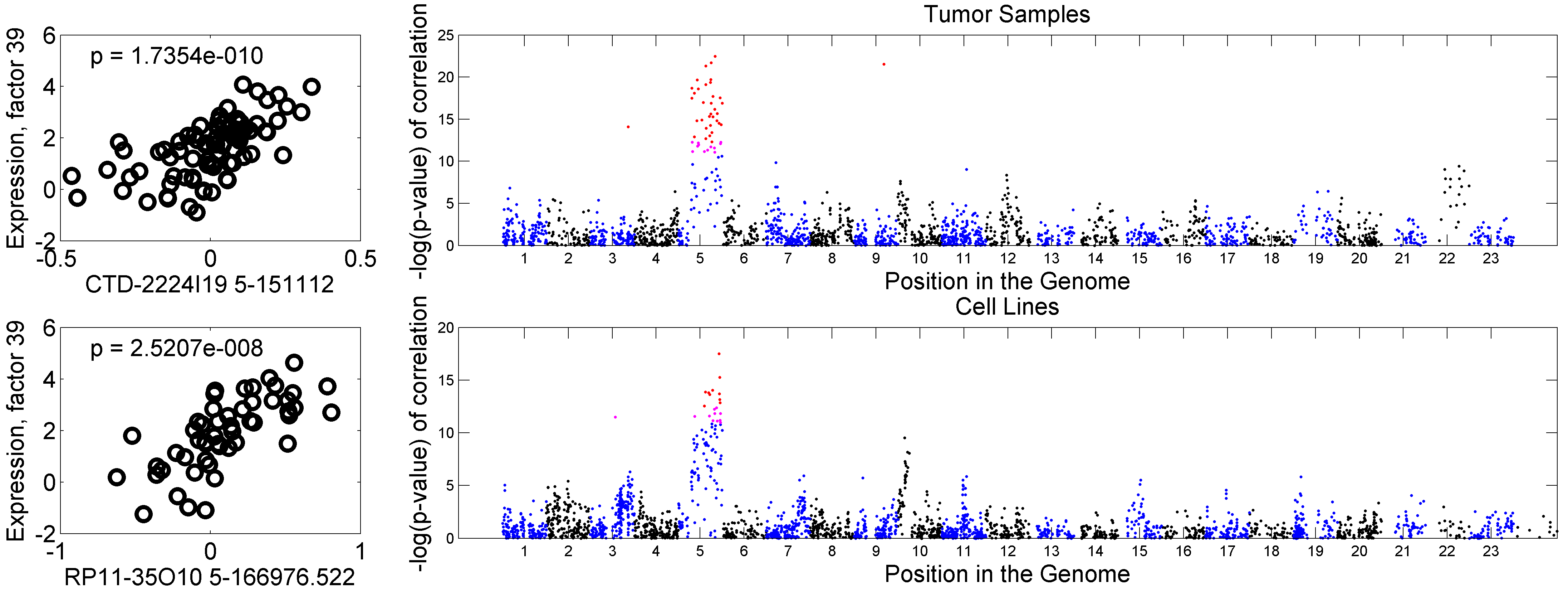

Supplement: Figure S4 — The association of the BAC clones with the expression of CNA-associated factors. (2.15 MB ZIP) [file pcbi.1000920.s004.zip › factor39_CNV.png]

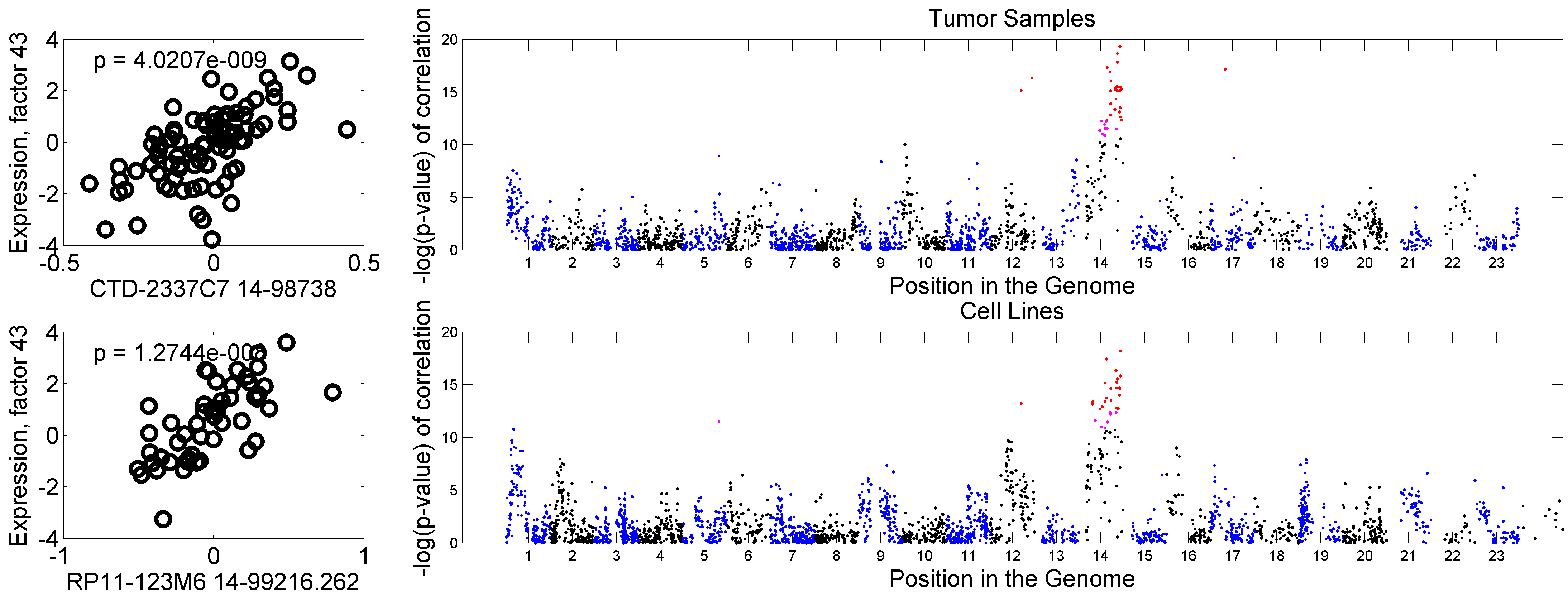

Supplement: Figure S4 — The association of the BAC clones with the expression of CNA-associated factors. (2.15 MB ZIP) [file pcbi.1000920.s004.zip › factor43_CNV.png]

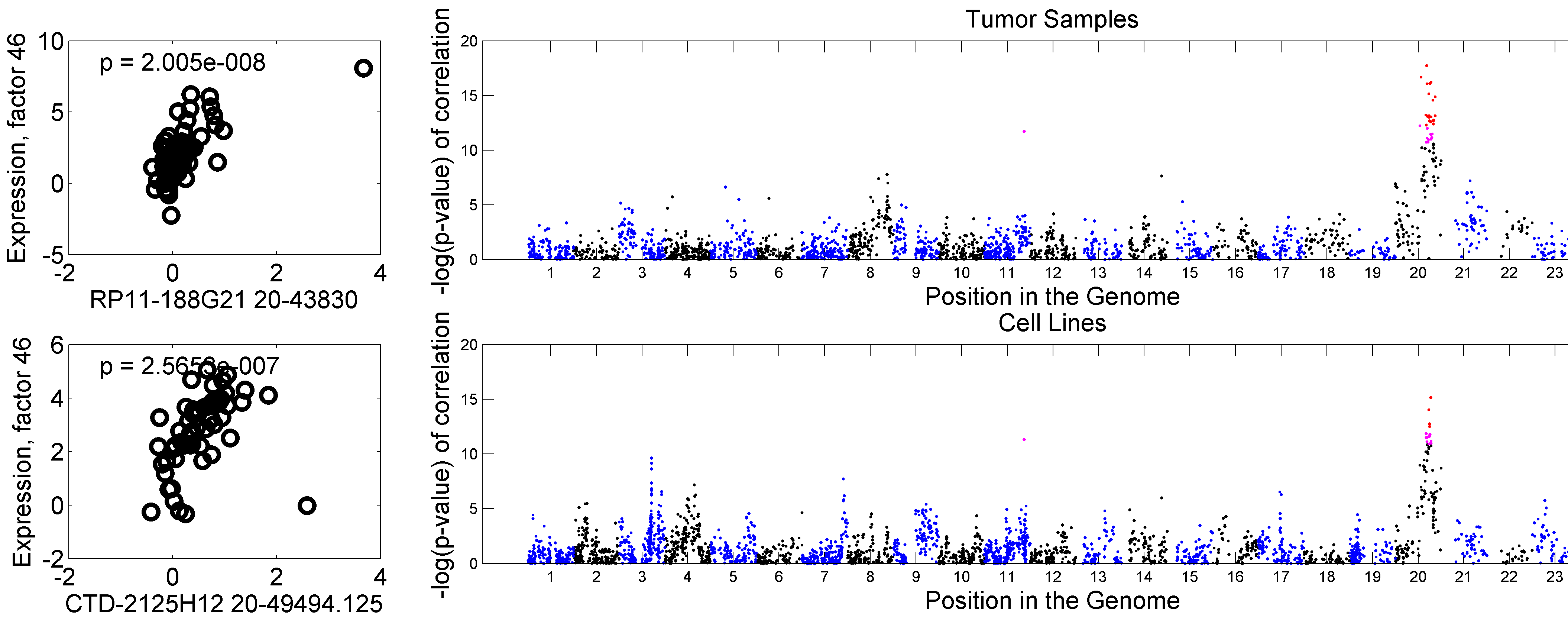

Supplement: Figure S4 — The association of the BAC clones with the expression of CNA-associated factors. (2.15 MB ZIP) [file pcbi.1000920.s004.zip › factor46_CNV.png]

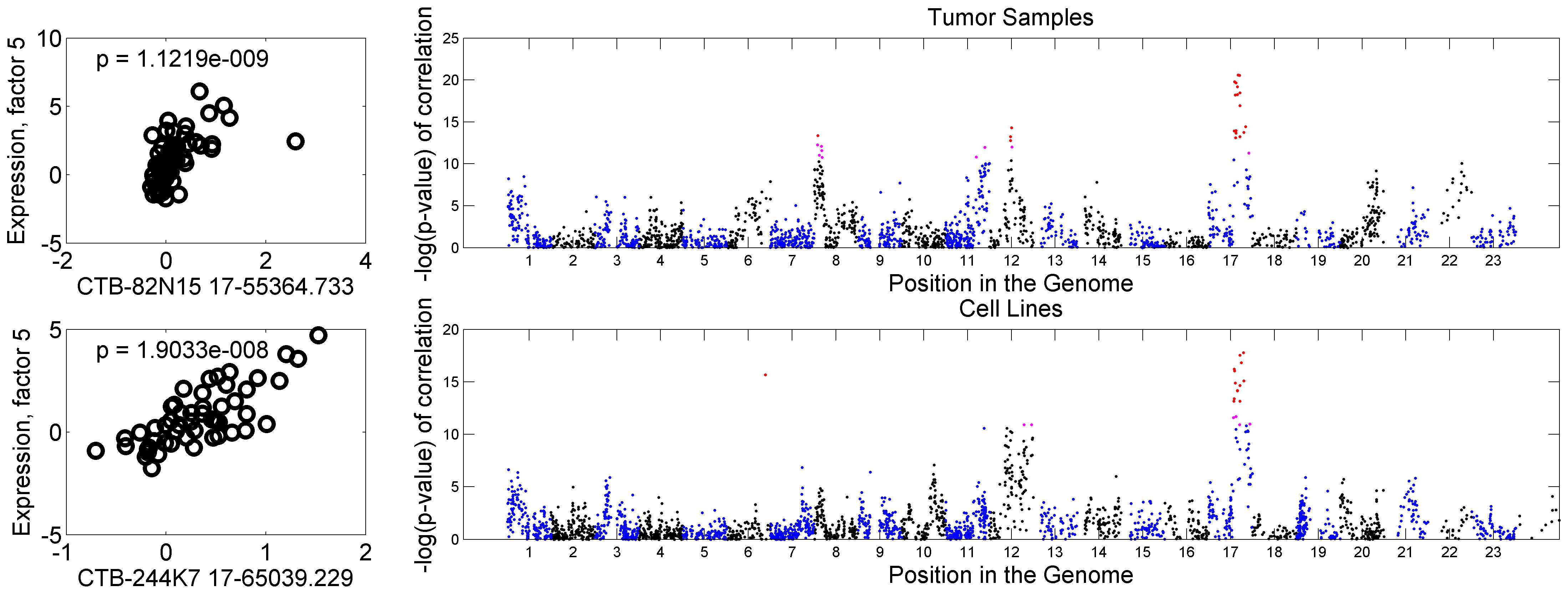

Supplement: Figure S4 — The association of the BAC clones with the expression of CNA-associated factors. (2.15 MB ZIP) [file pcbi.1000920.s004.zip › factor5_CNV.png]

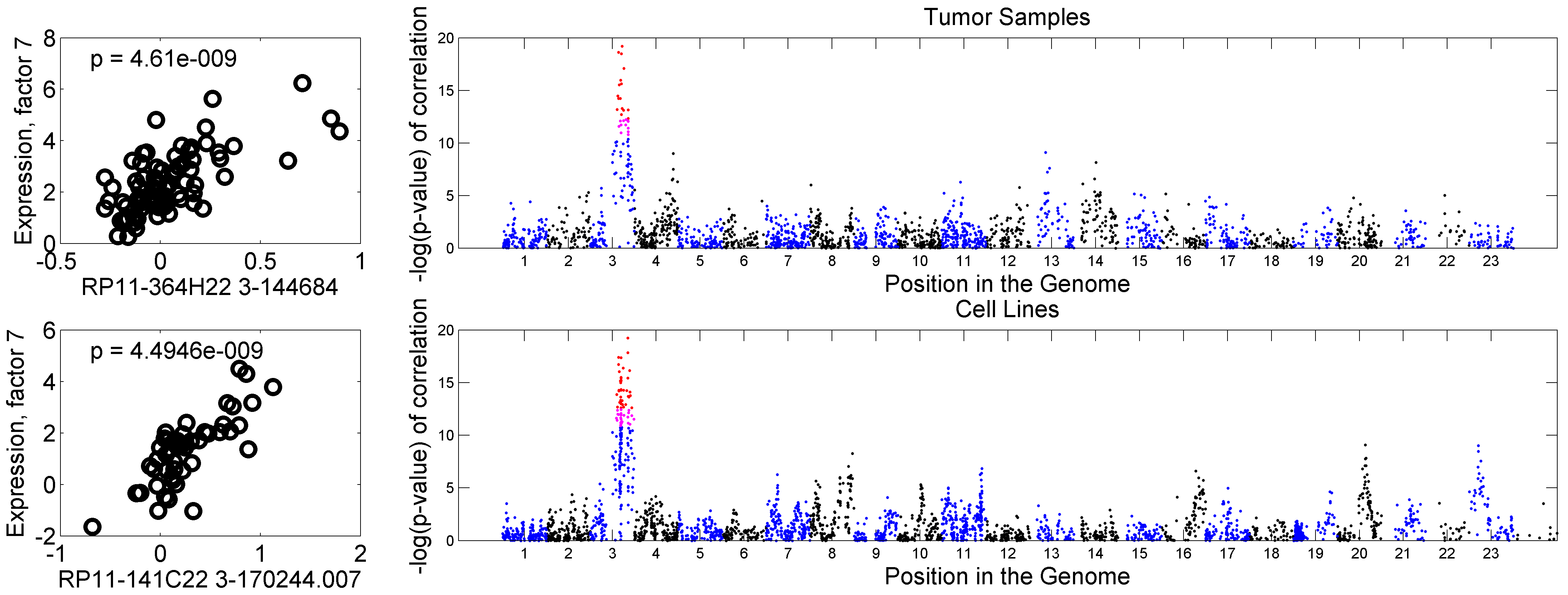

Supplement: Figure S4 — The association of the BAC clones with the expression of CNA-associated factors. (2.15 MB ZIP) [file pcbi.1000920.s004.zip › factor7_CNV.png]

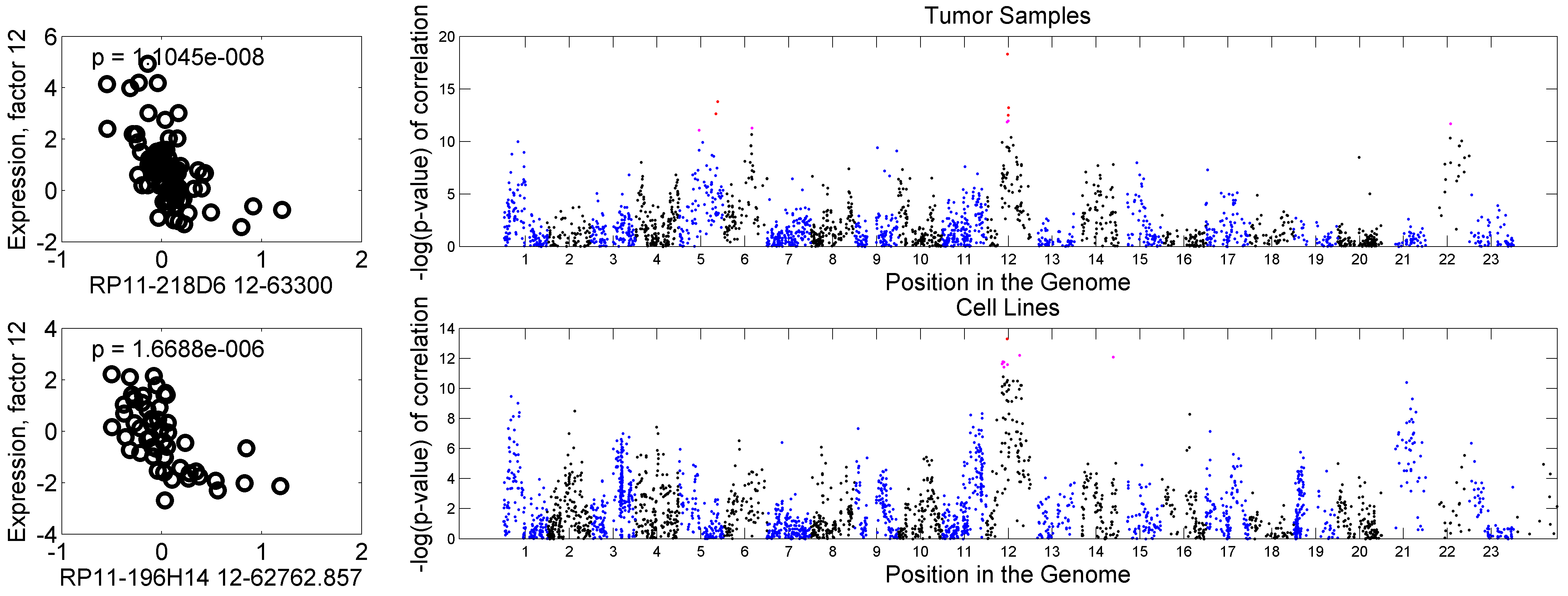

Supplement: Figure S4 — The association of the BAC clones with the expression of CNA-associated factors. (2.15 MB ZIP) [file pcbi.1000920.s004.zip › factor12_CNV.png]

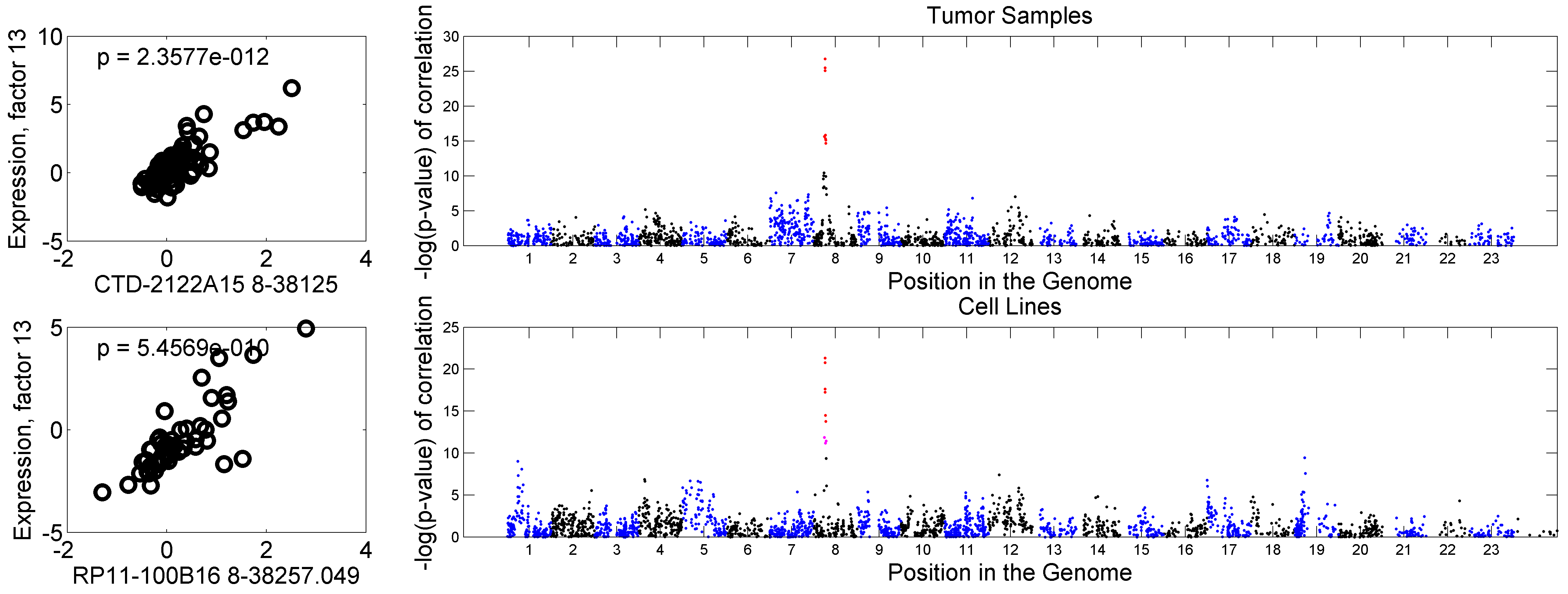

Supplement: Figure S4 — The association of the BAC clones with the expression of CNA-associated factors. (2.15 MB ZIP) [file pcbi.1000920.s004.zip › factor13_CNV.png]

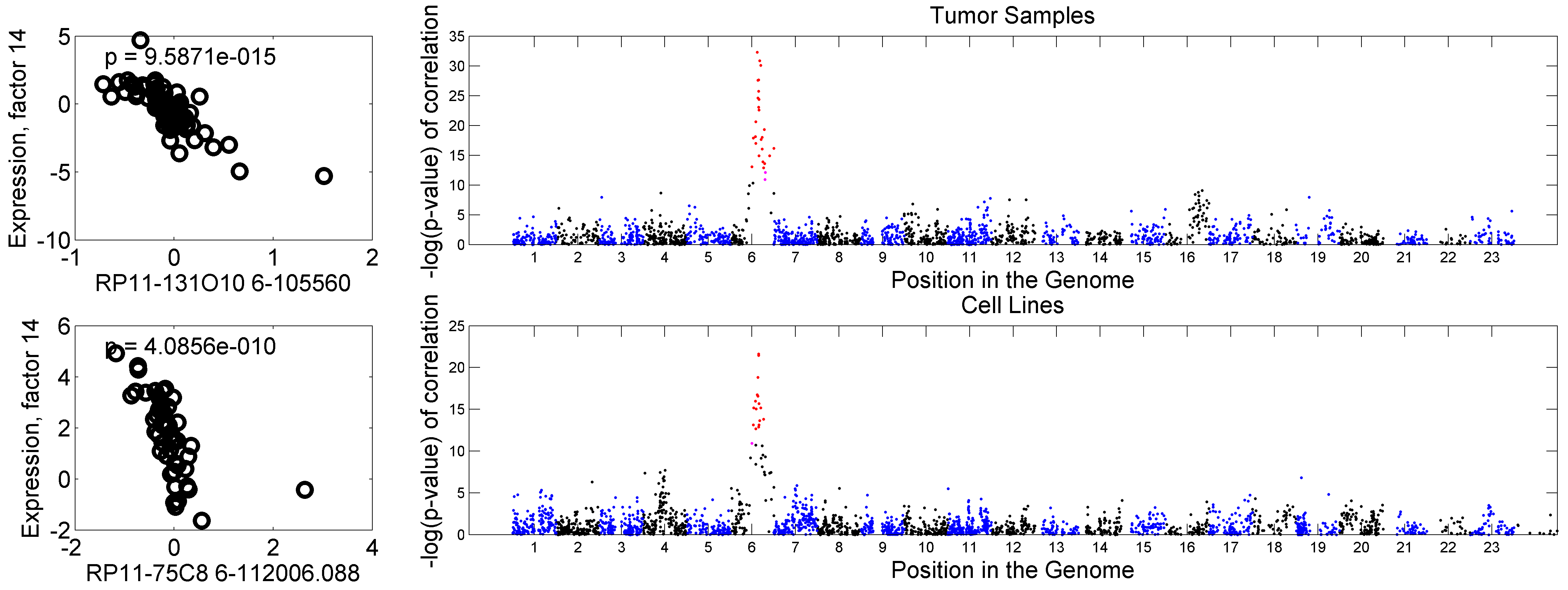

Supplement: Figure S4 — The association of the BAC clones with the expression of CNA-associated factors. (2.15 MB ZIP) [file pcbi.1000920.s004.zip › factor14_CNV.png]

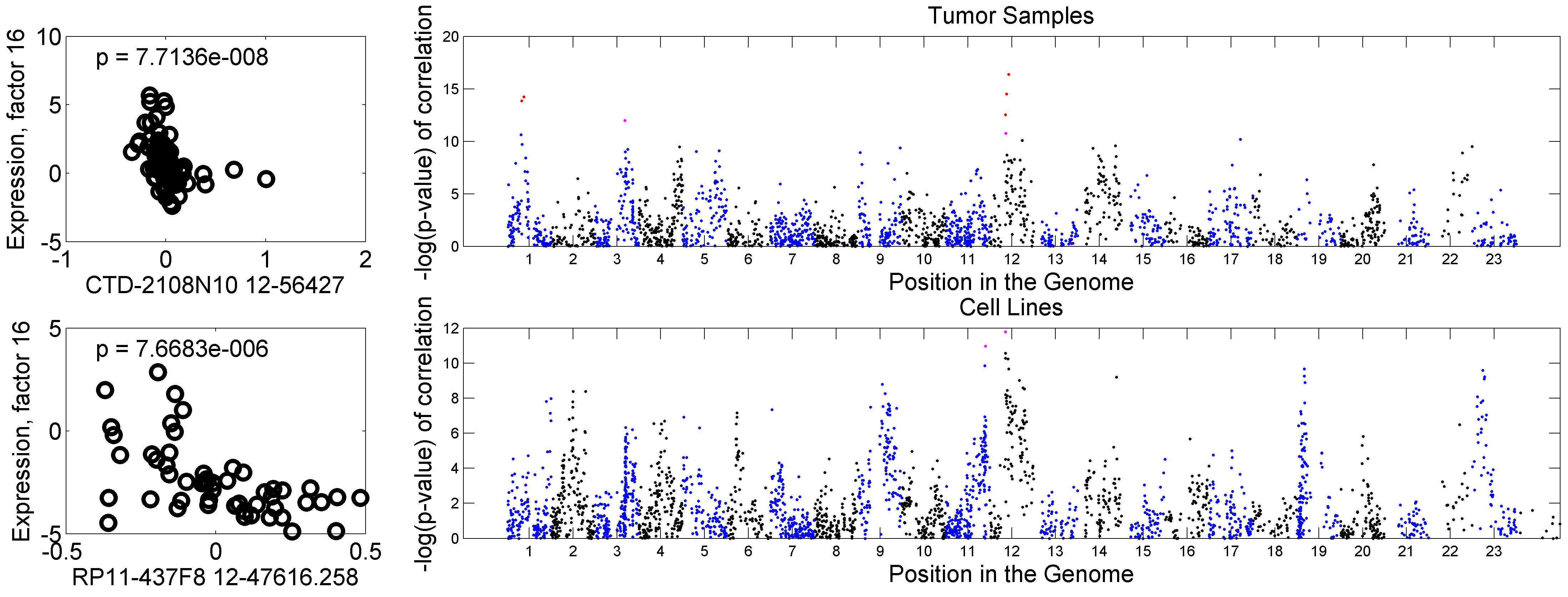

Supplement: Figure S4 — The association of the BAC clones with the expression of CNA-associated factors. (2.15 MB ZIP) [file pcbi.1000920.s004.zip › factor16_CNV.png]

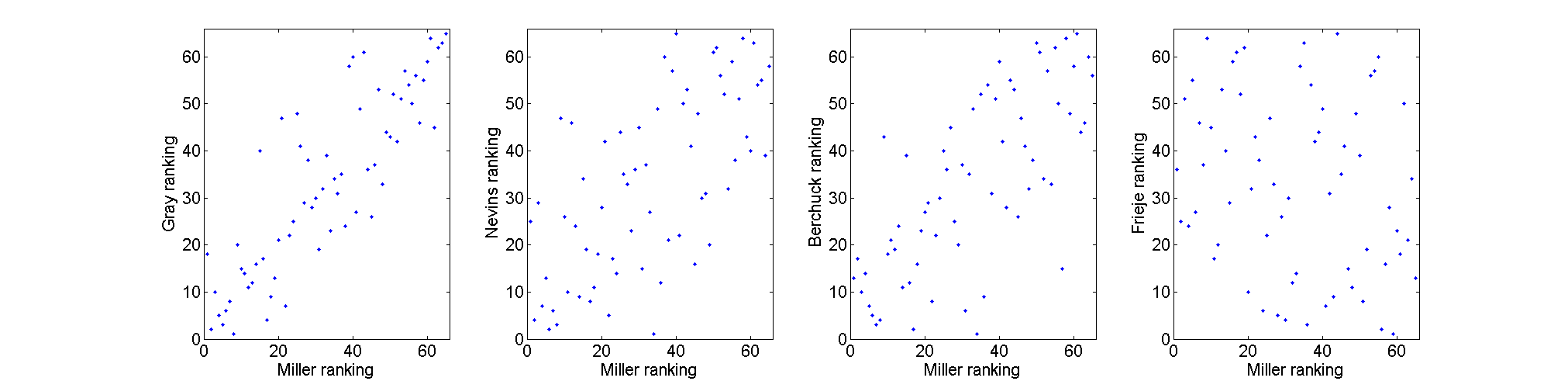

Supplement: Figure S5 — Scatterplots comparing the ranking of genes in factor 26 as computed with each of the 5 different data sets. High levels of correlation indicate a factor that is conserved between the two data sets. (0.09 MB TIF) [file pcbi.1000920.s005.tif]
